# Results of Antiretroviral Treatment Interruption and Intensification in Advanced Multi-Drug Resistant HIV Infection from the OPTIMA Trial

**DOI:** 10.1371/journal.pone.0014764

**Published:** 2011-03-31

**Authors:** Mark Holodniy, Sheldon T. Brown, D. William Cameron, Tassos C. Kyriakides, Brian Angus, Abdel Babiker, Joel Singer, Douglas K. Owens, Aslam Anis, Ruth Goodall, Fleur Hudson, Mirek Piaseczny, John Russo, Martin Schechter, Lawrence Deyton, Janet Darbyshire, for the OPTIMA Team

**Affiliations:** 1 VA Palo Alto Health Care System, Palo Alto, California, United States of America; 2 Department of Medicine, Stanford University, Stanford, California, United States of America; 3 James J. Peters VA Medical Center, Bronx, New York, United States of America; 4 Department of Medicine, Mt. Sinai School of Medicine, New York, New York, United States of America; 5 University of Ottawa at The Ottawa Hospital Research Institute, Ottawa, Ontario, Canada; 6 Canadian HIV Trials Network, St Paul's Hospital, Vancouver, British Columbia, Canada; 7 VA Cooperative Studies Program Coordinating Center, West Haven, Connecticut, United States of America; 8 Nuffield Department of Medicine, The John Radcliffe Hospital, University of Oxford, Oxford, United Kingdom; 9 MRC Clinical Trials Unit, London, United Kingdom; 10 Department of Veterans Affairs, Office of Public Health and Environmental Hazards, Washington, D.C., United States of America; University of California San Francisco, United States of America

## Abstract

**Background:**

Guidance is needed on best medical management for advanced HIV disease with multidrug resistance (MDR) and limited retreatment options. We assessed two novel antiretroviral (ARV) treatment approaches in this setting.

**Methods and Findings:**

We conducted a 2×2 factorial randomized open label controlled trial in patients with a CD4 count ≤300 cells/µl who had ARV treatment (ART) failure requiring retreatment, to two options (a) re-treatment with either standard (≤4 ARVs) or intensive (≥5 ARVs) ART and b) either treatment starting immediately or after a 12-week monitored ART interruption. Primary outcome was time to developing a first AIDS-defining event (ADE) or death from any cause. Analysis was by intention to treat. From 2001 to 2006, 368 patients were randomized. At baseline, mean age was 48 years, 2% were women, median CD4 count was 106/µl, mean viral load was 4.74 log_10_ copies/ml, and 59% had a prior AIDS diagnosis. Median follow-up was 4.0 years in 1249 person-years of observation. There were no statistically significant differences in the primary composite outcome of ADE or death between re-treatment options of standard versus intensive ART (hazard ratio 1.17; CI 0.86–1.59), or between immediate retreatment initiation versus interruption before re-treatment (hazard ratio 0.93; CI 0.68–1.30), or in the rate of non-HIV associated serious adverse events between re-treatment options.

**Conclusions:**

We did not observe clinical benefit or harm assessed by the primary outcome in this largest and longest trial exploring both ART interruption and intensification in advanced MDR HIV infection with poor retreatment options.

**Trial Registration:**

Clinicaltrials.gov NCT00050089

## Introduction

Despite the effectiveness of current antiretroviral (ARV) treatment (ART) [1], [2], past sequential development and availability of ARVs, significant toxicities and partially effective combinations left many persons with multi-drug resistant (MDR) HIV and limited re-treatment options [3], [4], [5], [6]. In developing countries where ART has been more recently introduced there are increasing numbers of people with limited re-treatment options [7]. This is due to treatment-emergent drug resistance and the lack of or limited newer alternative ARVs that are more potent and non-cross-resistant, yet more expensive. MDR HIV is ultimately associated with increased risk of AIDS-associated diseases and death [8], [9].

Optimal medical management remains unclear for MDR HIV with limited re-treatment options. Clinical management strategies include either continuing current or alternative ARVs in an ART regimen of up to four ARVs, intensifying ART with at least five and up to nine ARVs [10], [11], [12] chosen for expected tolerance and activity, or interrupting ART for a period of careful clinical observation [13], [14], while maintaining or providing other relevant prophylaxis and treatment regimens before re-initiation of ART.

Several studies have addressed treatment interruption in patients with MDR HIV and are reviewed elsewhere [13]. While controlled comparisons vary in context of different populations, adequacy of re-treatment, and duration of ART interruption, these studies have shown no consistent or lasting advantage in terms of virological or CD4 count response. One study reported an increase in AIDS-defining events (ADEs) after ART interruption and retreatment, in particular esophageal candidiasis, but this study showed no difference in health-related quality of life (HRQoL) or survival [15], [16]. The use of ART interruption is currently not supported by the US Department of Health and Human Services (DHHS) therapeutic guidelines panel [17], and the appearance of new ARVs such as enfuvirtide, darunavir, etravirine, maraviroc and raltegravir offers very promising re-treatment options for treatment failure of nucleoside and non-nucleoside reverse transcriptase inhibitors (NRTI, NNRTI) and protease inhibitor (PI) ARV regimens [18]. However, for the majority of patients with treatment-emergent MDR HIV infection in those countries with the greatest burden of HIV and resource constraint, where conventional ART has been recently introduced, there are very limited retreatment options.

In addition, health outcomes other than AIDS related disease have emerged as very important indicators of burden and impact of health in advanced HIV disease [19]. These include non-HIV related serious adverse events (SAEs), co-morbidities such as viral hepatitis, cardiovascular and metabolic diseases, and HRQoL measures [20], [21], [22], [23].

Our primary hypothesis was that ART intensification (so called mega-ART) would result in increased clinical benefit in terms of prolonging life and delaying the occurrence of new or recurrent AIDS events compared to continued standard treatment. A 2×2 factorial study design allowed us to test a second hypothesis regarding interruption, along with standard treatment or intensification. At the time of study development and protocol approval, conflicting data existed as to whether there was any benefit of interruption, for any duration; and only uncontrolled data existed as to the possible benefit of intensification. Our initial hypothesis regarding interruption was that it would result in a clinical benefit, and that when combined with intensification, any clinical benefit would be further increased. Our objective therefore was to investigate these clinical management strategies for MDR HIV infection in advanced disease with limited ARV retreatment options.

## Materials and Methods

The protocols for this trial and supporting CONSORT checklist are available as supporting information; see Checklist S1, Protocol S1, and publication of the trial design and methods [24].

### Study Design and Outcomes

OPTIMA was a randomized 2×2 factorial clinical trial conducted in three countries (USA, Canada, and UK) at over 70 clinical centers. The trial was registered at http://clinicaltrials.gov, number NCT00050089. Primary outcome was time to first new or recurrent AIDS-defining illness or death from any cause. Secondary outcome was time to first non-HIV related SAE. Outcomes were reviewed by an Endpoints Review Committee (ERC) blinded to randomization, classified either as ADEs according to the U.S. CDC revised AIDS case definition [25] and pre-defined criteria (OPTIMA protocol, http://www.hivnet.ubc.ca/e/home/optima/protocol/), or as adverse events. Deaths (by ERC) were reviewed for attribution (HIV, ART, neither or uncertain), and all SAEs (by STB and DWC) were reviewed for attribution (AIDS, HIV, ART, non-HIV, or not determined).

### Study Ethics

The protocol was approved by independent Research Ethics Boards at each site. The trial was performed in accordance with the principles of Good Clinical Practice and the Declaration of Helsinki. All volunteers signed written informed consent before any trial related procedure.

### Study Population

HIV-1 infected patients were recruited between June 2001 and June 2006, and followed until study closure on December 31, 2007. They were eligible if the clinician was considering a change of ART because they had twice failed NRTI based ART including an NNRTI or a PI, or had genotypic or phenotypic evidence of three-class (NRTI, NNRTI and PI) ARV resistance, and they were currently receiving ART for at least three months. Other inclusion criteria included a CD4 count ≤300/µl, HIV-1 plasma viremia ≥5000 copies/ml (HIV-1 Amplicor Monitor 1.0 or 1.5, Roche, Branchburg, NJ), or ≥2500 (Versant 3.0, Siemens, Deerfield, IL). Exclusions were age < eighteen years, pregnancy, nursing mothers, AIDS-defining illness within fourteen days of screening, or likelihood of imminent death.

### Study Procedures

Randomization was stratified by CD4 count at screening (above or below 100/µl) and by study site, according to a computer-generated randomization list, in permuted blocks of randomly varying size by the coordinating center to re-treatment with (a) either initial treatment interruption for an intended period of 12 weeks followed by a new ART regimen, or a change of ART regimen without interruption, and (b) change in ART by intensification (to ≥5 ARV) or standard ART (of ≤4 ARV). For patients randomized to ART interruption before re-treatment, the assigned standard or intensified ART option was not communicated to the physician or the patient until the end of the planned interruption. The ARVs in the retreatment regimens were chosen by the treating physician according to treatment history and expected tolerance, and by recent or screening susceptibility testing. Low-dose ritonavir (≤400 mg/day) was not counted as an ARV. Patients were recruited, consented and followed-up in the clinics where they received care for HIV infection and followed-up at 6, 12, 24 and then every 12 weeks after randomization with additional visits at 2 and 6 weeks after treatment interruption and after re-treatment initiation. Blood samples were collected at screening for genotypic resistance testing and virtual phenotype (vP, VircoType, Virco, Mechelen, Belgium) and at screening and follow-up visits for HIV-1 viral load (VL), CD4 and CD8 T lymphocyte count and routine hematological and biochemical blood tests. Follow-up ARV resistance testing was permitted at the discretion of the treating physician. Incremental phenotypic susceptibility score (PSS) from the baseline vP was calculated as previously described [26]. Newly available ARVs including those provided through expanded access protocols and other non-conflicting clinical trials were permitted.

A patient was considered compliant to protocol if after assignment to a treatment interruption it was continued for at least 6 weeks, if they started retreatment within two weeks of their assigned time, and they initiated retreatment with the number of ARVs within the assigned standard or intensive ART option. Periods up to 30 days off ART or off the assigned number of drugs in the standard or intensive ART regimens were allowed. Patients were considered protocol compliant if the reason for change was stated to be high VL, low CD4, severe adverse event or intercurrent illness. Medication compliance was determined at each follow-up visit using an assessment tool adopted from the AIDS Clinical Trials Group adherence questionnaire [27]. We also assessed HRQoL, healthcare utilization and costs, which are described [19], [28] and to be reported elsewhere.

### Study Population and Sample Size Justification

The original study design [29] made the following assumptions: 1) an event rate in the standard ART group at Year 1 of 20%; with a 25% increase annually thereafter; 2) Two-sided Type I error (*alpha*)  = 0.05; 3) Loss to follow-up at 3.5 years of 10%; 4) cross-over from standard to intensive ART of 5% in year 1, increasing 10% every year thereafter; 5) cross-over from intensive to standard ART of 20% in year 1, decreasing by 50% every year thereafter. With 652 outcomes in 1700 subjects, a 22% relative reduction in hazard would be detected with 93% power. During the study sample size was revised based on observed accrual, outcome and crossover rates, with extended accrual and follow-up periods but no change in other assumptions, such that 261 outcomes in 390 patients would provide a study power of 75%. Also, a change was implemented in the UK due to lack of equipoise among treating physicians regarding ART interruption, to permit a choice of only one of the two randomizations.

### Statistical Analysis

Analyses were performed by intention to treat, according to randomly allocated management strategy. Analyses of the primary outcome and survival comparisons were performed using time-to-event methods including Kaplan-Meier plots and the stratified log-rank tests. Analyses also included interaction of treatment assignments, length of follow-up, or calendar time of enrollment on the primary outcome. Treatment differences were estimated by hazard ratios using Cox proportional hazards regression. Other comparisons including outcomes stratified by baseline CD4 count (< or >100 and by quartiles <36, 36–110, 111–196, >196/µl) were made with standard parametric and non-parametric statistical tests. An independent Data and Safety Monitoring Board monitored results on an ongoing basis.

### Role of Funding Source

The study funders (MRC-UK, VACSP, CIHR) reviewed and approved the design and conduct of the trial, with external expert reviewers who were represented on the Trial Steering Committee. The OPTIMA Writing Committee and the OPTIMA team had full access to the statistical report and to the study data by request and the Writing Committee had final responsibility for the submitted manuscript.

## Results

**Figure 1:**
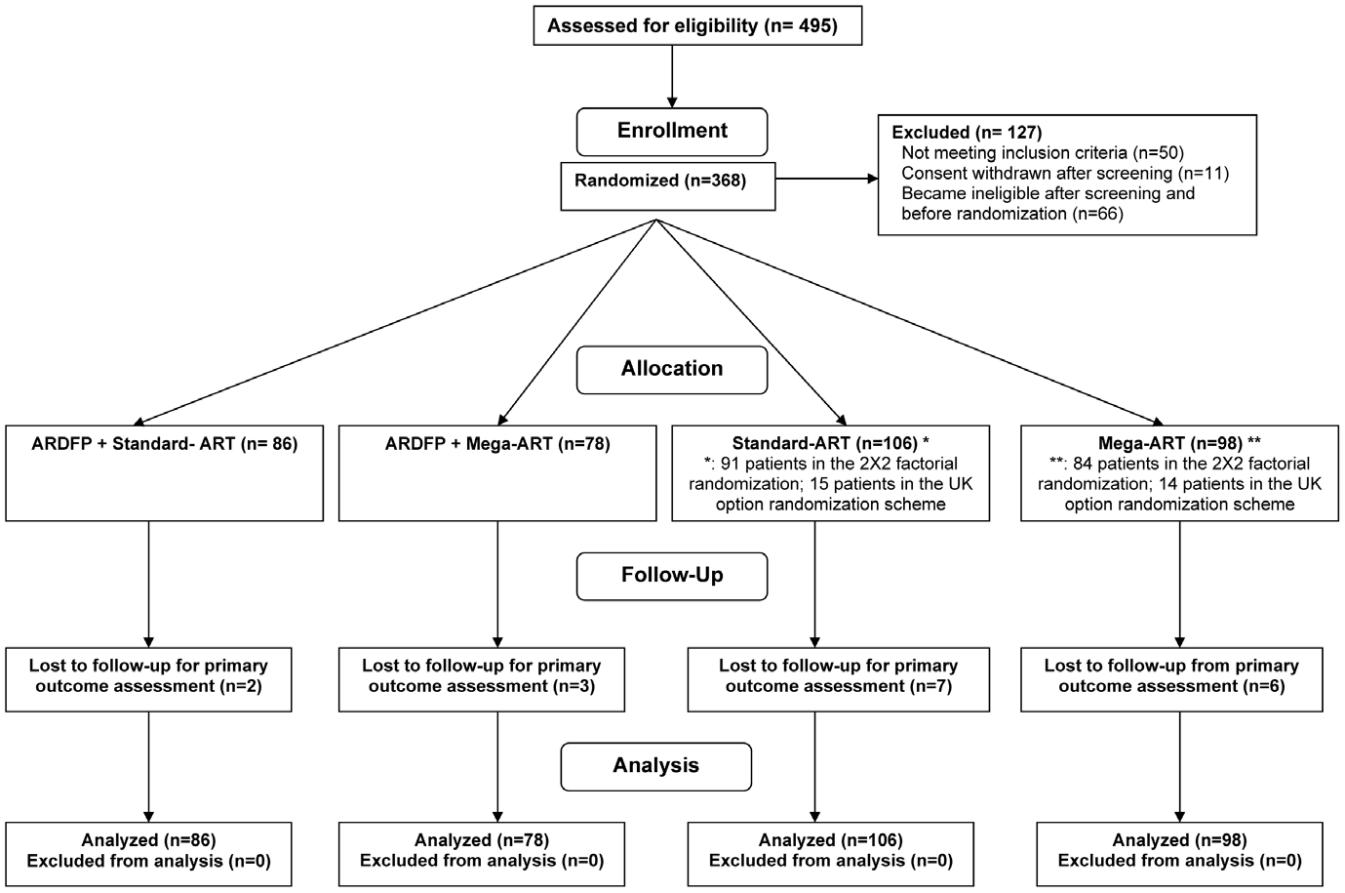
Trial profile and patient flow.

Of the 457 patients screened, 368 patients were enrolled (VA 288, 78%; Canada 41, 11%; and UK 39, 11%), of whom 339 were randomized to the 2×2 factorial and 29 were randomized to standard vs. intensification-ART only (Figure 1). The baseline clinical characteristics of the study population are presented in Table 1. The study population was 98% male with a mean age of 48 (SD 8.5) years; with mean and median CD4 counts of 127 and 106/µl respectively and mean plasma HIV-1 VL of 4.74 log_10_ copies/ml.

**Table 1:** Baseline characteristics by treatment strategy.

	Standard vs. Intensive ART			ART Interruption vs. Continuation		
	Standard ART	Intensive ART	Total	ART Interruption	ART Continuation	Total
**Number of patients Randomized**	192	176	368	164	175	339
**Mean Age in years (SD)**	48.4 *(8.65)*	47.6 *(8.31)*	48.0 *(8.49)*	48.7 *(8.32)*	48.5 *(8.47)*	48.6 *(8.39)*
**Age Categories (%):**						
31–40	36 *(19)*	32 *(18)*	68 *(18)*	27 *(16)*	27 *(15)*	54 *(16)*
41–50	77 *(40)*	79 *(45)*	156 *(42)*	66 *(40)*	76 *(43)*	142 *(42)*
51–60	65 *(34)*	56 *(32)*	121 *(33)*	59 *(36)*	61 *(35)*	120 *(35)*
>60	14 *(7)*	9 *(5)*	23 *(6)*	12 *(7)*	11 *(6)*	23 *(7)*
**Gender (%):**						
Male	189 *(98)*	172 *(98)*	361 *(98)*	160 *(98)*	172 *(98)*	332 *(98)*
Female	3 *(2)*	4 *(2)*	7 *(2)*	4 *(2)*	3 *(2)*	7 *(2)*
**Race (%):**						
White	90 *(47)*	90 *(51)*	180 *(49)*	73 *(45)*	87 *(50)*	160 *(47)*
Black	80 *(42)*	64 *(36)*	144 *(39)*	67 *(41)*	71 *(41)*	138 *(41)*
Asian	2 *(1)*	0 *(0)*	2 *(1)*	0 *(0)*	1 *(1)*	1 *(0)*
Hispanic	15 *(8)*	21 *(12)*	36 *(10)*	21 *(13)*	15 *(9)*	36 *(11)*
Aboriginal	2 *(1)*	0 *(0)*	2 *(1)*	1 *(1)*	1 *(1)*	2 *(1)*
Other	3 *(2)*	1 *(1)*	4 *(1)*	2 *(1)*	0 *(0)*	2 *(1)*
**Mode of Infection (%):**						
Blood	20 *(10)*	15 *(9)*	35 *(10)*	15 *(9)*	19 *(11)*	34 *(10)*
Heterosexual	47 *(24)*	39 *(22)*	86 *(23)*	41 *(25)*	39 *(22)*	80 *(24)*
IDU	27 *(14)*	25 *(14)*	52 *(14)*	24 *(15)*	27 *(15)*	51 *(15)*
MSM	87 *(45)*	88 *(50)*	175 *(48)*	76 *(46)*	79 *(45)*	155 *(46)*
Other	9 *(5)*	9 *(5)*	18 *(5)*	6 *(4)*	11 *(6)*	17 *(5)*
Unknown	2 *(1)*	0 *(0)*	2 *(1)*	2 *(1)*	0 *(0)*	2 *(1)*
**AIDS or prior AIDS at entry (%)**	107 *(56)*	109 *(62)*	216 *(59)*	95 *(58)*	105 *(60)*	200 *(59)*
**Hepatitis B (HBsAg+) (%)**	26 *(14)*	13 *(7)*	39 *(11)*	23 *(14)*	14 *(8)*	37 *(11)*
**Hepatitis C (anti-HCV Ab+) (%)**	46 *(24)*	34 *(19)*	80 *(22)*	39 *(24)*	38 *(22)*	77 *(23)*
**HIV RNA copies/ml ** ***(%)***						
<5k	14 (7)	17 (10)	31 (8)	19 (12)	12 (7)	31 (9)
5–50k	72 (38)	58 (33)	130 (35)	56 (34)	68 (39)	124 (37)
50–100k	35 (18)	28 (16)	63 (17)	32 (20)	27 (15)	59 (17)
>100k	70 (37)	73 (41)	143 (39)	56 (34)	68 (39)	124 (37)
mean log_10_ (SD)	4.74 (*0.62*)	4.74 (*0.75*)	4.74 (*0.68*)	4.67 (*0.71)*	4.75 (*0.66)*	4.71 (*0.68)*
**CD4 cells/µl**						
Mean (SD)	129 (107)	125 (106)	127 (107)	129 (106)	131(109)	130(108)
median	109	102	106	109	111	110
**Virtual Phenotypic Susceptibility Score (PSS) (mean)**	1.3	1.7	1.5	1.4	1.5	1.5

The study population was heavily ARV-experienced: 96% had taken over three NRTI (median 5, interquartile range, IQR, 4–6), 97% at least one NNRTI (median 1, IQR, 1–2), 63% at least three PI (median 3, IQR, 2–5) and 2.5% enfuvirtide. In the retreatment regimens, 171 (99.4%) of 172 standard-ART and 139 (91.5%) of 152 intensification-ART patients were protocol-compliant in the number of ARVs used with a median number of ARVs of 3 in standard and 5 in intensive ART regimens. The mean number of active ARVs as determined by PSS was 1.3 in the standard and 1.7 in the intensive groups (p<0.03), and 1.4 in the interruption and 1.5 in the no interruption groups (P = NS). The median time to protocol non-compliance resulting in a change in retreatment strategy was 187 (IQR 79,-) weeks for standard, versus 59 (IQR 19,155) weeks for intensification-ART (p<0.001, log-rank test). There was no significant difference in time to changing treatment strategy between the ART interruption before retreatment versus immediate ART strategy. For those assigned ART interruption, the median duration of the interruption was 12 weeks (IQR 12–14 weeks). Use of primary and secondary opportunistic infection prophylaxis was high at baseline, with over 80% of patients overall taking anti-PCP, 25% taking other anti-fungal, and 45% taking other antibacterial medications. This level of prophylaxis was maintained during the study (in patients with CD4<200 cells/mm^3^) with 83%, 53%, 59% taking anti-PCP, other anti-fungal, and antibacterial medications at the last follow-up visit respectively.

A total of 165 (44.8%) of 368 subjects experienced a primary outcome. This included 67 deaths without a preceding ADE, and 98 ADE of whom 61 died subsequently. Of the 165 primary outcomes, the most common were death (40.9%), esophageal candidiasis (18.3%), *Pneumocystis jirovecii* pneumonia (PCP) (8.5%), cytomegalovirus disease (4.8%), HIV wasting syndrome (4.3%), *Mycobacterium avium* complex infection (3.7%), Kaposi's sarcoma (2.4%), and cryptococcosis (2.4%). There was no significant difference in the number or type ADE between re-treatment options. The ERC adjudicated 52% of deaths to be HIV-related, 2% due to ART medication, 15% unrelated to HIV or ART, and 31% as unattributable.

**Figure 2:**
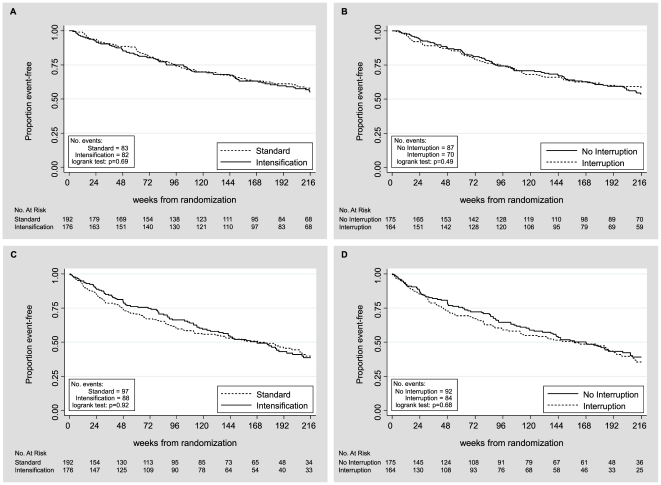
Time to first AIDS event or death by treatment strategy. A) Intensification *versus* standard antiretroviral therapy (ART); B) ART interruption *versus* continuation. Time to first non HIV-related serious adverse event (SAE) by C) intensification *versus* standard ART; D) ART interruption *versus* continuation.

Of 368 randomized to standard versus intensive retreatment, there were respectively 83 versus 82 ADE or death outcomes (p = 0.69, log-rank test; Figure 2A), 67 versus 61 deaths, and 47 versus 51 first ADE (Table 2). Time to the primary outcome of ADE or death did not differ between the two groups (HR 1.17, 95% CI 0.86–1.59; p = 0.33) (Table 2). There were 339 randomized to treatment continuation versus interruption before retreatment, and there were respectively 87 versus 70 ADE or death outcomes (p = 0.49; log-rank Figure 2B), 62 versus 61 deaths, and 58 versus 35 ADE. Time to ADE or death did not differ significantly between the two groups (HR 0.93, 95% CI 0.68–1.3; p = 0.64) (Table 2). ADEs and deaths were lower after treatment interruption (Table 2), but the changes did not reach statistical significance.

**Table 2:** AIDS events and death by treatment strategy.

	Standard ART N (%)	Intensive ART N (%)	Total N (%)	ART Interruption N (%)	ART Continuation N (%)	Total N (%)
Number assessed	192	176	368	164	175	339
**AIDS Events**						
Total number of AIDS events	74	73	147	47	94	141
New	53	52	105	34	69	103
Recurrent	21	21	42	13	25	38
Total number of patients having one or more new or recurrent AIDS events	47 (24.5)	51 (29.0)	98 (26.6)	35 (21.3)	58 (33.1)	93 (27.4)
**Survival**						
Total number of deaths	67	61	128	61	62	123
Definitely/Probably HIV-related	30	36	66	25	38	63
Definitely/Probably ART-related	1	1	2	1	1	2
Uncertain HIV or ART-related	22	18	40	26	13	39
Unlikely HIV or ART-related	14	6	20	9	10	19
Total number of patients having new or recurrent AIDS event or death	83 (43.2)	82 (46.6)	165 (44.8)	70 (42.7)	87 (49.7)	157 (46.3)
Total follow-up time (years)*	697.9	644.9	1342.8	569.4	685.6	1255
Total at risk follow-up time (years)+	614.8	572.8	1187.6	524.3	582.8	1107.1
Primary outcome rate per 100 person years	13.5	14.3	13.9	13.4	14.9	14.2

*Follow-up calculated as time from randomization to last assessment or death.

+Follow-up at risk calculated as time from randomization to first AIDS event or death, or last assessment.

Across the four retreatment approaches of continuation + standard, continuation + intensification, interruption + standard, interruption + intensification in 339 subjects there were respectively 46, 41, 35 and 35 ADE or death outcomes, there were 35, 27, 30 and 31 deaths, and there were 30, 28, 16 and 19 first ADE. There was no significant difference in the time to ADE or death across the four treatment options (p = 0.87, log-rank test). Significantly more patients with baseline CD4 ≤100/µl had a new or recurrent ADE with continuation (38 (46.3%)) compared to with interruption (23 (30.7%), p = 0.04). In the lowest baseline CD4 quartile (< 36/µl) more deaths occurred with intensification than standard ART retreatment (34 (70.8%) *versus* 23 (48.9%), p = 0.03), and more had ADE or death (39 (81.3%) *versus* 26 (55.3%), p = 0.007). Finally, tests of main effect interactions demonstrated no statistical significance in AIDS events or death when treatment assignment, length of follow-up, or calendar time of enrollment were considered (Tables 3, 4, 5, 6).

**Table 3:** First AIDS event or death by treatment strategy: stratified analysis.

Outcome	Management Comparison	Hazard Ratio	95% CI	p-value
AIDS event or death	intensification/standard; interruption/continuation*	1.1650.927	0.856, 1.5850.674, 1.275	0.330.64
Death	intensification/standard; interruption/continuation*	1.1281.420	0.793, 1.6040.986, 2.045	0.500.06
AIDS event	intensification/standard; interruption/continuation*	1.2950.696	0.866, 1.9350.455, 1.065	0.210.09

*: Excludes UK Option patients.

**Table 4:** First AIDS event or death by treatment strategy: unstratified, 2X2 factorial analysis (includes main effects interaction).

Outcome	Management Comparison	Hazard Ratio	95% CI	p-value*
AIDS event or death	intensification/standard; interruption/continuation	0.970.75	0.71, 1.330.53, 1.07	0.27
Death	intensification/standard; interruption/continuation	0.951.14	0.57, 1.560.7, 1.85	0.61
AIDS event	intensification/standard; interruption/continuation	0.990.50	0.66, 1.490.30, 0.84	0.25

Note: excludes UK Option patients.

*: Test for interaction.

**Table 5:** First AIDS event or death by treatment strategy and by follow-up time.

Comparison	Number Events or Death	Hazard Ratio	95% CI	p-value*
Intensification vs. standard ART *(All patients)*				
1^st^ year of follow-up	28 vs. 22	0.943	0.354, 1.360	0.24
2^nd^–7^th^ year of follow-up	54 vs. 61	1.405	0.804, 2.455	
Intensification vs. standard ART *(2*×*2 factorial-excludes UK Option patients)*				
1^st^ year of follow-up	26 vs. 21	0.883	0.606, 1.285	0.21
2^nd^–7^th^ year of follow-up	50 vs. 60	1.372	0.772, 2.439	
ART interruption vs. continuation *(2*×*2 factorial-excludes UK Option patients)*				
1^st^ year of follow-up	24 vs. 23	0.806	0.552, 1.178	0.33
2^nd^–7th year of follow-up	46 vs. 64	1.134	0.640, 2.009	

*Test for heterogeneity.

**Table 6:** First AIDS event or death by treatment strategy and by calendar time.

Comparison	NumberEvents or Death	Hazard Ratio	95% CI	p-value*
Intensification vs. standard ART *(**All patients**)*				
15Jun01–31Dec03	25 vs. 27	0.949	0.550, 1.636	0.76
01Jan04–31Dec06	42 vs. 41	0.997	0.650, 1.529	
01Jan07–31Dec07	15 vs. 14	1.319	0.637, 2.734	
Intensification vs. standard ART *(**2**×**2 factorial-excludes UK Option patients**)*				
15Jun01–31Dec03	25 vs. 26	1.006	0.581, 1.743	0.86
01Jan04–31Dec06	37 vs. 41	0.924	0.593, 1.445	
01Jan07–31Dec07	14 vs. 14	1.174	0.560, 2.462	
ART interruption vs. continuation *(**2**×**2 factorial-excludes UK Option patients)***				
15Jun01–31Dec03	27 vs. 24	1.057	0.610, 1.834	0.18
01Jan04–31Dec06	34 vs. 44	0.923	0.590, 1.448	
01Jan07–31Dec07	9 vs. 19	0.437	0.198, 0.965	

*Test for heterogeneity.

185 of 368 (50%) patients developed 481 non HIV-related SAEs. There was no significant difference in number or time to first non HIV-related SAE between standard vs. intensive ART retreatment (log-rank p = 0.92, HR 1.008, 95%CI 0.75–1.35), or between interruption versus continuation (log rank p = 0.68, HR 1.08, 95%CI 0.8–1.46) (Figures 2C and D).

**Figure 3:**
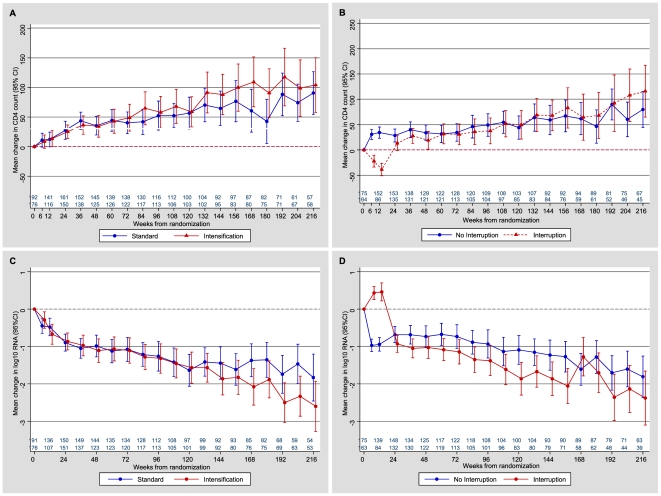
Immunological and virological changes over time by treatment strategy. A) CD4 count change, intensification vs. standard antiretroviral therapy (ART); B) CD4 count change, ART interruption vs. continuation; C) HIV-1 viral load change, intensification vs. standard ART; D) HIV-1 viral load change, ART interruption vs. continuous.

Expected changes in CD4 lymphocyte counts and plasma HIV viral load were observed (Figure 3); viremia measures and CD4 appeared to at least recover over time after interruption and re-treatment. After week 24, there was no significant difference in CD4 count or viremia between groups.

## Discussion

There are randomized control trials in retreatment of treatment-failing or drug-resistant HIV infection with unpromising retreatment options, which evaluate new ARVs [30] or treatment intensification and treatment interruption of varied duration [13], [14], [15], [16]. New ARV drug classes with low cross-resistance are rightly favoured when available, and there is evidence that the more ‘active’ ARVs used the better the outcome. Treatment intensification with familiar drug classes is expensive and seen as desperate, while treatment interruption has not been shown to be beneficial. OPTIMA was challenged by slow accrual due to a small number of patients with MDR-HIV who were willing to accept allocation to retreatment options that could be obtained without joining a trial, by clinical heteropoise - a lack of equipoise which varies in degree and direction - with respect to intensification and interruption, and by new ARV development trials which offered a chance of perceptibly favorable retreatment ARV regimens. This was further demonstrated in the UK, where some treating physicians did not feel it was appropriate to consider both strategies in OPTIMA and so randomization was split to offer patients a choice. In addition, there was competition in the UK for enrolling patients with MDR-HIV in trials of newer agents that were perceived to be likely more potent than the strategies offered in OPTIMA. Nevertheless, OPTIMA is the largest and longest controlled trial of ART management options in MDR-HIV with advanced HIV immune deficiency and poor retreatment options, for both AIDS and non-HIV SAE and HRQoL outcomes. Although randomized management allocation in this trial was not blinded to patients or healthcare providers, the rigorous conduct, the clinical nature of outcomes and completeness of follow-up distinguish this trial from ARV activity trials measuring surrogate markers in smaller and shorter trials.

Although no significant advantage or disadvantage to any of the retreatment options was seen in over 1000 person-years of observation, confidence limits here may include a clinically significant effect that is not reflected in the simple outcome rates. For example, the data are consistent with a possible 14% decrease or 59% increase in death or ADE with treatment intensification; and approximately a 32% decline or 30% increase in death or ADE with treatment interruption. Unlike other reports, there was no excess risk of ADE with ART interruption in patients with the highest expected risk defined by lowest baseline CD4 counts. For example, Lawrence et al. reported on a controlled trial of 16-week treatment interruption, which showed an excess of treatment-responsive esophageal candidiasis, but no difference in HRQoL or in survival [15], [16]. This difference in outcome could be related to a higher proportion of patients in the OPTIMA trial receiving anti-infective prophylaxis (87% for PCP and 53% for candidiasis with a CD4 count <200 per µl). Additional analysis was suggestive of a trend toward increased mortality following resumption of therapy in patients undergoing treatment interruption; while not statistically significant, this trend might provide some clinically important insight in therapeutic modalities.

No other trials of retreatment intensification evaluate clinical outcomes, and there is a valid concern for toxicity, tolerance and cost as well as efficacy of intensified ART. OPTIMA identified no significant overall benefit in health outcomes or harm in excess non-HIV SAEs or drug intolerance. However, maintenance of intensification was much less than a standard ART retreatment option so that no difference in cumulative additive or antagonistic effects from additional medications in MEGA-ART were seen.

In an ART era of new and very promising retreatment options of new class ARVs and combinations, what instruction may be taken from these trials of retreatment management options for HIV treatment failure? In the developing world, the greatest burden of HIV and the greatest healthcare resource constraints co-exist. Conventional ARV roll-outs for the many untreated are still expanding and leave little room to allocate added resources for the inevitable adherence- and intolerance-driven failures of ART and treatment-emergent MDR-HIV. In clinical practice, treatment interruption is a common occurrence. A recently completed trial in Uganda exploring short, intermittent HIV treatment interruption in a healthier population found that 7 days on and 7 days off ARVs resulted in more virologic failures compared to 7 day or 5 day continuous treatment cycles which yielded comparable results [31]. Although the treatment population, strategy and measured outcome from this study is not directly comparable to the MDR patients in OPTIMA, it demonstrates that some forms of ARV interruption strategy may be used as a compromise in the right clinical context.

OPTIMA suggests that in some settings of ART failure with limited prospects for re-treatment, individualization of retreatment for interruption or intensification with conventional ARVs may be considered, particularly with good clinical follow-up and management of opportunistic infection risk. Such decisions should carefully weigh both potential benefits and harms. As trials of this nature in these types of settings are unlikely in the future, a pooling of existing trial datasets may offer the best opportunity to identify principles and particular populations which may gain or lose most in ARV retreatment choices after failure of ART, MDR-HIV and limited retreatment options.

## Supporting Information

Checklist S1 CONSORT Checklist.
(0.16 MB RTF)

Protocol S1 Trial Protocol.
(1.06 MB PDF)
