# Supplementary material for: Results of Antiretroviral Treatment Interruption and Intensification in Advanced Multi-Drug Resistant HIV Infection from the OPTIMA Trial
Source: PLoS One. 2011 Mar 31;6(3):e14764. doi: 10.1371/journal.pone.0014764 (PMC3069000; doi:10.1371/journal.pone.0014764)
Supplement: Checklist S1 — CONSORT Checklist. (0.16 MB RTF) [file pone.0014764.s001.rtf]

 
CONSORT Statement 2001 Checklist  

Items to include when reporting a randomized trial      
 

 
From Moher D, Schulz KF, Altman DG. The CONSORT statement: revised recommendations for improving the quality of reports of parallel-group randomised trials. Lancet 2001; 357(9263): 1191-1194. 
 
The CONSORT Statement 2001 checklist is intended to be accompanied with the explanatory document that facilitates its use. For more information, visit www.consort-statement.org. 
