# Supplementary material for: Results of Antiretroviral Treatment Interruption and Intensification in Advanced Multi-Drug Resistant HIV Infection from the OPTIMA Trial
Source: PLoS One. 2011 Mar 31;6(3):e14764. doi: 10.1371/journal.pone.0014764 (PMC3069000; doi:10.1371/journal.pone.0014764)
Supplement: Protocol S1 — Trial Protocol. (1.06 MB PDF) [file pone.0014764.s002.pdf]

TRI-NATIONAL TRIAL 1: *Opt*ions *I*n *M*anagement with *A*nti-Retrovirals

A Randomized Controlled Trial To Determine The Optimal Management of Patients with HIV Infection for Whom First and Second-Line Highly Active Antiretroviral Therapy Has Failed

Conducted in the
United Kingdom, Canada & the Department of Veterans Affairs Healthcare System (US)

PROTOCOL

March 19, 2003

VERSION 2.0

TABLE OF CONTENTS

		Page
I.	Protocol Changes Chronology	
II.	Executive Summary 4	
III.	Abstract	8
IV.	Background, Rationale and Relevance 10	
V.	Study Objectives	15
VI	Study Outcome Measures	16
VII	Study Design	17
VIII.	Patient Population	21
	a. Inclusion Criteria	
	b. Exclusion Criteria	
IX.	Trial Procedures and Assessments	24
	a. Screening	
	b. Baseline Evaluations	
	c. Randomization	
	d. Follow-up Evaluations	
	e. Assessment of compliance	
	f. Reporting of Death, HIV disease progression and Adverse Events	
	g. Schedule of Evaluations and Forms	
X.	Treatment Regimens	31
	a. Antiretroviral Medications	
	b. Opportunistic Infections (OI) Prophylaxis	
	c. Treatment failure/guidelines	
	d. Maintenance of allocated treatment strategy and modifications	
	e. Patient follow-up	
XI.	Biostatistical Considerations	34
XII.	Study Administration	35
	a. Organizational Structure	
	b. Monitoring Patient Intake/Probation, Termination of Participating Sites	
	c. Monitoring Clinical Site Performance	
	d. Site Visits	
	e. Data Safety Monitoring	
	f. Endpoint Review	
XIII.	Publications	43
	a. Publication Policy	
XIV.	Sub-studies	45

XV. References 48

XVI. Health Economics Component 50

Appendices

1. Sample Informed Consent 77
2. Presumptive and Definitive Criteria for AIDS events in OPTIMA 85
3. NIAID Standardized Toxicity Grading System 88
4. ICH Standards for Serious Adverse Event Reporting 92
5. Antiretroviral Treatment Table:
Dosage, Interactions, Contraindications and Adverse Events 100
6. DHHS OI Prophylaxis guidelines 106
7. Sample Size/Power Calculations/Level of Significance 145
8. Statistical Analysis Plan 152
9. Statistical Reports/Shell Tables 161
-Trial Monitoring
-DSMB Monitoring Tables
10. Study Feasibility 181

VA Specific Appendices

A. Human Rights Considerations 196
1. Declaration of Helsinki
2. Human Rights Committee Minutes
3. Informed Consent Form
B. Budget 231
1. Budget Justification
2. Budget
C. Curricula Vitae 233
D. Case Report Forms/U-Titer Screens 266
E. Specimen Handling Procedures 318
F. Central Laboratories 321
1. MAVERIC
2. VIRCO

I. PROTOCOL CHANGES CHRONOLOGY

Revised Final Protocol v2.0 (March 19, 2003) with amendment and corrections to Version 1.1

- **Numbering of Sections revised upwards by one**
- **Executive Summary added as Section II**
- **Trial Sample size revised and Accrual period extended**

After evaluating the recently released results from two randomized studies (GIGHAART and the MDR-HIV study) and reviewing the accumulated data in OPTIMA, it was determined that the original sample size estimate was based on too conservative assumptions about the primary event (progression to a new AIDS defining event or death), treatment crossover and loss to follow-up rates.

Hence the sample size was revised, yet maintaining the conservative nature in the assumptions. Sample size re-estimation was done based on accumulated data from 142 patients enrolled in OPTIMA (by the end of October 2002). The following table provides a summary of the revised assumptions used for sample size revision.

ORIGINAL ASSUMPTIONS	REVISED ASSUMPTIONS
- Standard-ART cumulative event rate at Year 1 is **13%** with a 25% annual increase thereafter until the end of the study<br>- Crossover (Mega to Standard) is **20%** in Year 1; decreases 50% every year thereafter<br>- Hazard reduction of 30% with full adherence (unadjusted for losses and crossovers; adjusted hazard reduction 22.7%)<br>- Loss to follow-up at **3.5** years is **10%**<br>- Drop-in (Standard to Mega) is **5%** Year 1; increases 10% every year thereafter<br>- Alpha-level of 0.05<br>- **2.5** year accrual; 1 year minimum follow-up<br>- Power of 80%<br><br>Sample size: **1700**<br>Number of events: **450**	- Standard-ART cumulative event rate at Year 1 is **23%** with a 25% annual increase thereafter until the end of the study<br>- Crossover (Mega to Standard) is **5%** in Year 1; decreases 50% every year thereafter<br>- Hazard reduction of 30% with full adherence (unadjusted for losses and crossovers; adjusted hazard reductions are shown in table 3)<br>- Loss to follow-up at **5.5** years is **5%**<br>- Drop-in (Standard to Mega) is **1%** in Year 1; increases 10% every year thereafter<br>- Alpha-level of 0.05<br>- **4.5** year accrual; 1 year minimum follow-up<br>- Power of 80%<br><br>Sample size: **504**<br>Number of events: **292**

- **Section III: Abstract**

  As a result of the sample size revision, the average follow-up and the number of patients to be randomized have been changed. Also, the number of participating sites has been edited to reflect the current status in the three countries.

- **Section IV: Background and Rationale**

  This section has been edited to include recent knowledge on therapeutic strategies in HIV/AIDS disease management, as it pertains to the kind of patients enrolled in the OPTIMA study.

- **Section VII: Study Design**

  This section has been revised to reflect the changes in accrual and follow-up time and the revised sample size.

- **Section VIII: Patient Population**

  The number of participating sites in the three countries has been revised to reflect current status of the trial; the rate of accrual has also been revised to agree with the revised sample size target.

- **Section XI: Biostatistical Considerations**

  With the revised sample size and the extension of the accrual period, the event rates in the study have been changed accordingly. Details of these changes are provided in Appendix 7.

- **Section XII: Study Administration**

  Under the description of the Data and Safety Monitoring Board (DSMB): the monitoring process at the interim looks has been revised and approved by the DSMB.

  Under the plans for Monitoring Patient Intake/Probation, Termination of Participating Sites: the target accrual rate has been revised for VA participating sites to reflect the new sample size

Under Endpoint Review: The composition and procedures of the Endpoint Review Committee have been revised to reflect the operating procedures of this committee within the trial.

- **Section XVI: Health Economics Component**

  This section has been revised to account for the revision of the sample size and the extension of the accrual period.

- **Appendix 1: Sample Informed Consent Form**

  This form has been revised to reflect the change in the sample size and the duration of follow-up.

- **Appendix 2: Presumptive and Definitive Criteria for AIDS Events in OPTIMA**

  This table has been revised to reflect the updated list of AIDS events and the criteria used by the Endpoints Review Committee to adjudicate them. A total of eight (8) new constitutional diseases and the criteria for their diagnoses have been added; one constitutional disease has been deleted from the table.

- **Appendix 7: Sample Size/Power Calculations/Levels of Significance**

  This appendix has been revised to include the parameters that were used in the sample size re-estimation: (a) recent data from other studies; (b) event, cross-over and loss-to-follow-up rates based on data from the OPTIMA study as of October 15,

2002. A table comparing the original assumptions and the revised assumptions is provided. A new sample size calculation table is provided.

- **Appendix 8: Statistical Analysis Plan**

  This appendix has been revised to reflect the analysis plan submitted to the Data and Safety Monitoring Board (DSMB) at its December 2002 meeting.

- **Appendix 10: Study Feasibility**

  This section has been revised to reflect the new, revised sample size for the trial. New feasibility assessments are provided in light of the revised target sample size. Also, the table of participating sites has been updated to reflect the current status of the trial.

-

II. EXECUTIVE SUMMARY

The OPTIMA trial addresses scientific questions that are significant and relevant to patients with HIV infection and the clinicians who treat them. OPTIMA, the first Tri-National collaboration, has been implemented by an international collaboration of 60 sites (25 – VA, 20 – Canada and 15 – U.K). It has been recruiting patients since June 1, 2001. At the end of October 31, 2002, 142 patients have been enrolled from 60 sites (12 sites are not open for enrollment yet) in the three countries: 116 US-VA, 19 in Canada and 7 in U.K; the total follow-up is 46 patient- years.

This management trial will investigate two therapeutic principles in anti-HIV treatment. The first is whether to temporarily interrupt anti-HIV treatment. The second is whether to increase the number of antiretroviral drugs in salvage therapy. Both are still unanswered clinical management questions of great importance. These questions have not be answered by any other trials, most of which focus on non-clinical outcomes. Three recently completed trials have reinforced the relevance of OPTIMA, by showing that mega-anti-retroviral (mega-ART) salvage therapy is tolerable, and that treatment interruption and re-treatment can favorably impact surrogate response markers.[1-3] Whether the balance of these responses and toxicities to treatment interruption and intensified drug therapy translate into significant clinical benefit is unknown.

There are no foreseeable new anti-HIV drug treatments that are safe, effective, and would not invoke treatment-emergent drug resistance in a way that would supplant the therapeutic principles tested in OPTIMA. OPTIMA will have lasting relevance to anti-HIV treatment, where there continue to be treatment failures with multi-drug resistant (MDR) HIV infection.

Treatment failures due to drug resistance were the rule before HAART was developed and put into wide use. Unfortunately, despite the significantly positive impact of HAART, today's anti-HIV treatments still fail at a predictable rate due to the limits of tolerance, side effects, variable adherence to these complicated regimens and development of resistance to these medications. As well, there is an increasing emergence of transmitted drug resistance in primary HIV infections, which will more than likely further limit efficacy even of initial therapies. Thus, OPTIMA's target patient population persists and will grow. In order to address this, clinicians continue to attempt to improve outcomes of HIV therapy among those patients for whom currently recommended treatment regimens have failed by either intensifying therapy (by adding more drugs to existing treatment regimens) and/or by offering treatment interruptions to decrease toxicities and improve adherence and possibly viral responses when treatment is restarted. Opinions about these treatment strategies are diverse, but strongly held. However, the effectiveness of treatment interruption and/or intensification in salvage therapy has not been proven in a controlled clinical endpoint study. Thus, OPTIMA is poised to define important principles in the management of anti-HIV treatment failures.

OPTIMA continues to have important relevance to practicing clinicians and patients with HIV. The documented increase in levels of drug-resistant HIV and the scientific importance of OPTIMA is growing. Despite the relevance of this study and multiple efforts to improve accrual, OPTIMA has been unable to achieve the accrual targets established for the original sample size of the trial (n=1700).

The primary hypothesized causes for the observed accrual problems are:

1. *The length of time required to obtain IRB approval for local sites to begin active recruitment*.
2. *A smaller than anticipated number of patients were eligible for the study because of the restrictive eligibility criteria.*
3. *Site specific issues (e.g., lack of clinician and/or patient equipoise; Mega-ART funding)*

The first issue has been nearly resolved. Presently, 57 of a total of 67 sites in the three countries are approved and functioning with the current Protocol 1.1: 23 of 25 in the US-VA, 20 of 22 in Canada and 15 of 20 in the U.K.

The second factor was partially addressed by revising the entry criteria (OPTIMA Protocol v1.1, March 19, 2002) as follows: CD4 and viral load criteria have been simplified and expanded to include patients with CD4 less than 300 cells/mm^3 and viral load greater than 5000 copies/ml.

Certain site-specific issues have emerged during the conduct of the trial. In general, lack of equipoise can constitute a significant barrier to recruitment in some trials. This may be amplified in a 2x2 factorial trial when there are two major comparisons underway. With respect to OPTIMA, it is clear that in some settings, investigators and/or potential volunteers already have firm opinions about the relative values of the interventions under study. There is certainly a belief in many quarters that treatment interruptions are not helpful, and this belief may be more strongly held now than when the study was being planned. Unfortunately, this appears to be based on both poorly controlled, observational data and not on reliable evidence. Similarly, there is lack of equipoise about the relative

value of standard vs. mega-ART, and this may be present in either direction. In some settings, mega-ART has virtually become standard of care, while in others, mega-ART is not supported by clinicians and/or paying agencies. The Trial Management Committee's understanding that these clinician and patient beliefs are based on insufficient reliable scientific evidence was supported by the Trial Steering Committee.

Lack of equipoise has its greatest effect on accrual in trials when the interventions under study such as mega-ART and drug-free periods can be provided to patients at their direct request, rather than through a randomization process. While the foregoing explains, in part, difficulties with accrual we have experienced thus far, it also underscores how important it is for the study to continue. When therapeutic strategies vault into widespread use based on inadequate or inconclusive data, this is anathema to evidence-based practice. Despite the difficulties that widespread use imposes on trials, it becomes even more imperative to subject the interventions to rigorous scientific evaluation.

The original sample size was calculated based on assumptions that have proven to be very conservative, particularly event and cross-over rates. The actual event rate seen in OPTIMA is similar to that in a comparable trial, the CPCRA MDR-HIV study in patients with similar baseline CD4 count. Thus, the revised sample size of 504 is realistic, likely to achieve the scientific goals of the study and, with an extension of the accrual period, is achievable at the current rate of accrual and at some cost savings.

These proposed changes and the rationale for them have been endorsed by the Trial Steering Committee (TSC) and the Data Safety and Monitoring Board (DSMB) as appropriate, based on sound scientific rationale and correct management of this study.

A realistic estimate of the accrual rate is 9-10 patients per month. During the last 4

months, accrual from the three countries has averaged 11 patients per month.

After evaluating the recently released results from two randomized studies (GIGHAART and the MDR-HIV study)[1,2] and reviewing the accumulated data in OPTIMA, it is apparent that the original sample size estimate was based on too conservative assumptions about the primary event (progression to a new AIDS defining event or death), treatment crossover and loss to follow-up rates. Sample size estimates are largely driven by the assumed primary event rate (the smaller the event rate, the larger is the required sample size), the expected treatment difference in the primary event rate, the treatment crossover rate and (to a lesser extent) the loss to follow-up rate. In light of new data the sample size was revised. Monitoring sample size assumptions is a standard procedure and an integral part of trial management, so that if accumulating data from the trial suggest substantial deviations from the original assumptions, sample size is revised (very often upwards). However, it is important that such revisions are not based on the observed treatment difference in the primary outcome. The TSC made their decision blinded to treatment differences in OPTIMA, hence no modification to the original expected treatment effect is made.

Revised Sample Size and Impact on Health Economics:

The health economics component of this trial will not be adversely affected by these changes. To the extent that estimates of cost-effectiveness will be based on modeling the event rates and disease progression observed during the trial to extrapolate survival over the patients’ life time, we will now have a longer observation period upon

which to model future events. With fewer patients in the study, the precision of the cost and outcome measurements will be affected. However, the trial was not initially powered according to cost and quality of life measures but rather according to clinical events. The uncertainty associated with cost-effectiveness results will be handled using methodology that is different from what is typically adapted for efficacy estimates emanating from randomized clinical trials. Specifically, we will adopt a Bayesian approach by reporting the probability that one treatment is the most cost-effective given the data rather than using classical hypothesis tests. We will implement this by estimating cost-effectiveness acceptability curves that show the probability that each management option is the most cost-effective conditional on the value the decision-maker places on additional health outcomes. We feel that this methodology will adequately handle the reduction in sample size by showing the implications of the reduced precision in cost and outcome estimates for the decision uncertainty faced by decision makers.

In summary, after careful consideration of the proposed sample size reduction and potential changes in protocol design, we feel that the economic analysis will still provide the policy decision maker with vital information upon which to base their funding decisions.

The revised sample size is still based on conservative assumptions, given data from similar studies and more importantly, current data from OPTIMA. We have assumed a lower primary event rate than currently observed in the study to allow for possible reduction in this rate in the future. If the current rates are maintained during the study, the required 292

primary events could be achieved over a shorter trial time. These sample size assumptions will continue to be monitored by the TMC, TSC and the DSMB.

A graphical presentation of the projected revised target sample size (Figure 2) clearly indicates that the projected accrual performance is realistic and achievable given the study's accrual performance to-date.

Figure 2

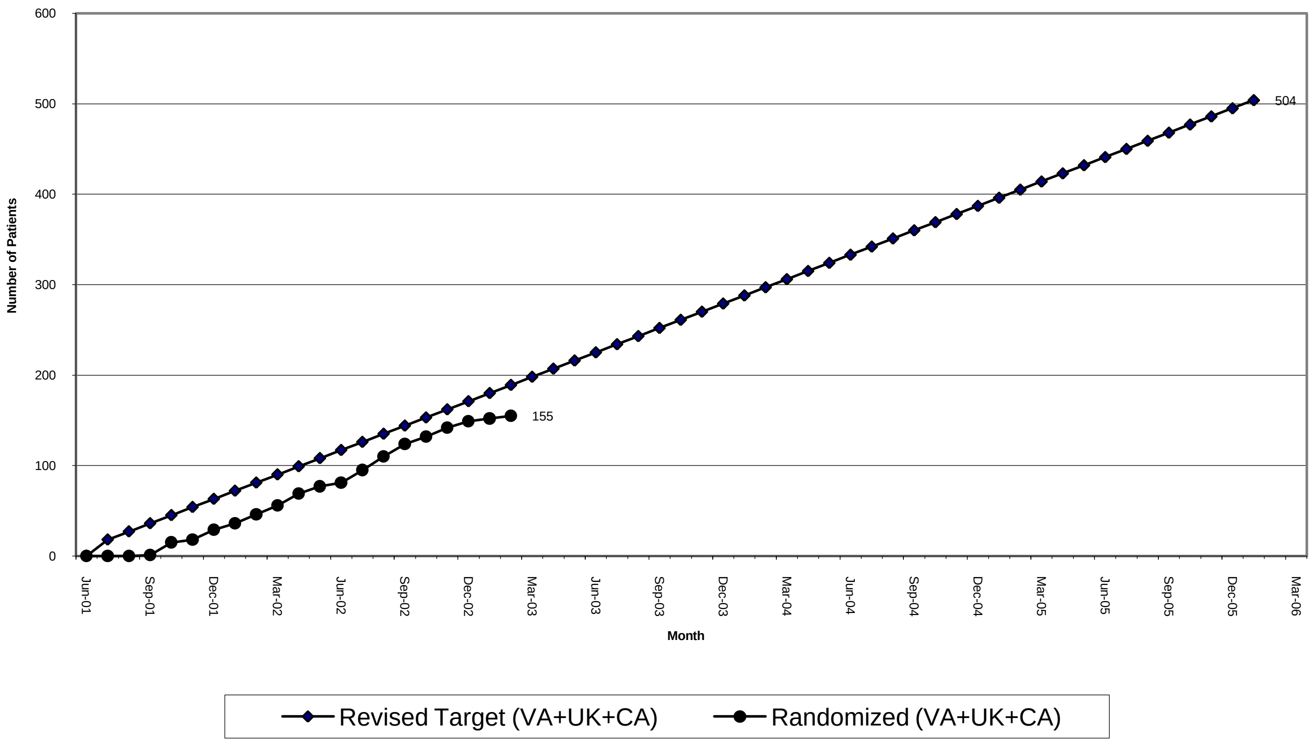

III. ABSTRACT

The introduction of highly active anti-retroviral therapy (HAART) has led to decreases in deaths and AIDS-defining opportunistic infections and cancers in HIV-infected patients. Unfortunately the benefits of HAART are not sustained in all patients and the available treatment options are consequently reduced, leading to a clinical dilemma in selecting anti-HIV treatments in patients for whom HAART has failed.

The purpose of the **OPT**ions **I**n **M**anagement with **A**nti-retrovirals (**OPTIMA**) tri-national (USA, UK and Canada) trial is to compare the effect of different management strategies on both clinical outcomes (survival, time to AIDS defining event, time to serious adverse event), virologic and immunologic response, and other healthcare outcomes (quality of life measures, resource utilization, cost-effectiveness) during an average of 3.5 years follow-up of patients with advanced HIV disease, in whom conventional anti-retroviral therapeutic (ART) regimens, including all three, currently available classes of anti-HIV drugs have failed. This trial will help delineate the most effective therapeutic strategy in the management of such patients.

The strategies to be compared in this 2X2 open randomized study are:

1) *Mega-ART* (5 or more anti-HIV drugs) versus *Standard-ART* (up to 4 anti-HIV drugs)
2) A *3-month Antiretroviral Drug-Free Period* (*ARDFP*) versus *No ARDFP*

The OPTIMA trial is the first large-scale, multicenter, randomized controlled trial to compare the relative efficacy of these different therapeutic strategies. The use of multiple

settings in different 'therapeutic cultures' will allow for generalizability of the findings and provide evidence that will facilitate management of HIV disease in this group.

A total of 504 patients will be randomized over a period of 4.5 years at 65 medical centers in the three countries. Patients will be followed at regular intervals during the 5.5 year study, until the last patient randomized has been followed for 1 year. The study is designed (taking into consideration drop-in, drop-out and loss to follow-up) to detect a 30% reduction in the relative hazard (under full compliance) between *mega* and *standard-ART* (2-sided α=0.05).

The trial funding will be tripartite: the Department of Veterans Affairs (VA) Cooperative Studies Program will provide funding for the 25 VA sites. The UK Medical Research Council (MRC) and the Canadian Institutes for Health Research (CIHR) will provide funding for 20 and 20 clinical sites respectively in their country.

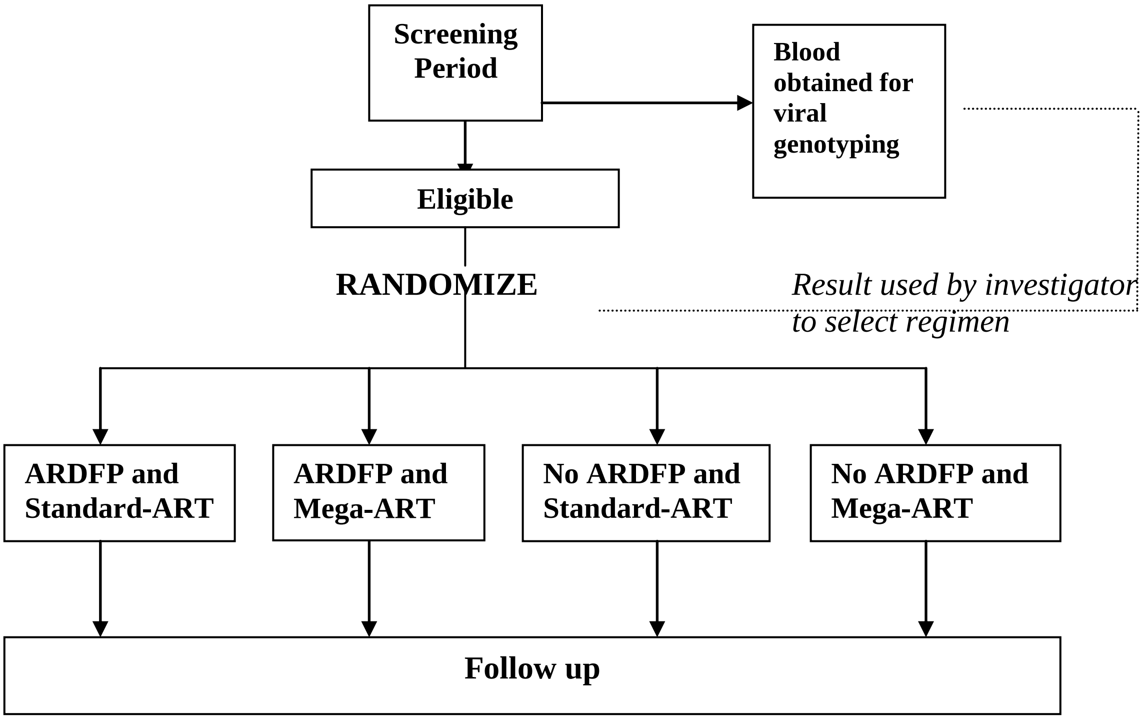

IV. Background, Rationale and Relevance

Triple drug anti-retroviral therapeutic regimens, commonly referred to as highly-active antiretroviral therapy (HAART), have resulted in a substantial decrease in the incidence of death and AIDS-defining opportunistic infections and cancers in patients infected with the human immunodeficiency virus (HIV). [4,5,6] The duration of response to these anti-retroviral regimens is often limited by the emergence of viral resistance or the development of toxicity, and there is increasing evidence from clinical practice that triple therapy fails a significant proportion of patients. A Swiss cohort of 2232 patients whose viral load became undetectable within 12 months of initiation of HAART demonstrated virologic failure rates of 35.7 to 40.1% within two years.[7]

Furthermore, subsequent treatment options are narrowed by the problem of drug resistance, which is complicated by the existence of cross-resistance within each of the three classes of anti-retroviral drugs.[8,9] An effective virologic and immunologic response may be elicited by changing as many drugs of the combination as possible, particularly if a new class of drug is used, and if the virologic breakthrough is of lower degree or shorter duration. In practice, unfortunately, the response to switching therapy to 3 or 4 new drugs after failure on the first treatment, is often transient. Once a prolonged and significant virologic breakthrough occurs on two different HAART regimens that have included all three classes of drugs, few treatment options are available with confidence, and the optimal management of such patients remains unclear.

Failure of successive regimens becomes increasingly likely due to mutational cross-resistance between drugs of the same class or decreased adherence caused by intolerance or

increased complexity of the remaining effective regimens. The introduction of newer medications may offer a respite but, with current knowledge, is unlikely to offer a solution to these trends. The clinical dilemma posed in selecting treatments for patients facing this situation is the focus of the OPTIMA trial and these treatment strategies will also be evaluated from a societal perspective with cost-effectiveness analyses.

Three general approaches have been tried in patients for whom first and second line HAART regimens have failed. First, the patient's current drug regimen or an analogous regimen (*standard-ART, comprised of up to 4 drugs*) is continued in the hope that it will have some continued therapeutic effect, through residual anti-HIV activity, and perhaps through maintenance of drug-resistant HIV of attenuated virulence. Second, a large number of anti-retroviral drugs selected based mainly on tolerability (*mega-ART, of 5 to 9 drugs*) including those on which the patient has failed in the past, is given in the hope that their combined anti-HIV activity will lead to maximal virologic suppression [10]**,** and immune recovery with acceptable tolerance. [11]Third, drug therapy is stopped temporarily (known variously as *'drug-free period', 'drug-holiday', 'structured treatment interruption'*) in order to reduce the symptoms of toxicity so that the new regimen may be better tolerated and in the hope that more sensitive viruses will re-emerge without continued drug pressure, which may allow superior anti-HIV activity and clinical efficacy of subsequent treatment. The latter phenomenon is biologically complex, and controversial. Information from case-series is gradually emerging,[12] but many questions remain unanswered.[13]

The probability of viral response to mega-ART, defined as a decline in viral load to below 500 copies/mL, increased with the length of ARDFP in one study. [14] It is hypothesized that, in the absence of pressure from drugs, most of the virus population

returns to wild-type, and is thus sensitive to drugs re-introduced after the ARDFP. Enhanced response to mega-ART has been observed in patients with at least 2 preceding months of ARDFP in spite of not unexpected falls in CD4 counts during the ARDFP. In addition, it is possible that a temporary ARDFP may provide the patient with an improved quality of life without seriously affecting long-term survival, or possibly even improving survival even with a standard-ART regimen because the patients are better able to tolerate the new therapy. The concept of treatment interruptions has been supported by NIH therapeutic guidelines which state “For patients with no rational alternative options who have virologic failure with return of viral load to baseline (pre-treatment levels) and declining CD4 T cell count, there should be consideration for discontinuation of anti-retroviral therapy”. [15] The optimum duration of an ARDFP , however, has not been determined.

The first question addressed by this trial is whether there is a difference in effectiveness between mega-ART and standard-ART regimens. The use of a large number of anti-retroviral drugs, referred to as 'mega-ART', is an experimental treatment strategy that has met at least some success, as defined by virologic response.[16] Essentially, the strategy is to treat with as many anti-retroviral drugs as possible (defined as 5 or more) and maintain them for as long as possible. In the largest case series to date, investigators reported on 106 heavily pre-treated patients with a median of 43 months of prior anti-retroviral therapy and a median plasma viral load (pVL) as measured by HIV RNA of 62,000 copies/mL.[10] As initially presented, patients received anywhere from 5 to 9 drugs as part of their mega-ART therapy and were followed for a median of 15 months. An on-treatment analysis showed that 48% of 88 subjects had pVL below 400 copies during weeks 47-57. On an intent-to-treat analysis, 40% of patients had pVL values <400 copies/mL between weeks 47 and 57 of

follow-up. Among those subjects who achieved suppression below 400 copies/mL on 2 consecutive measurements, the probability of remaining suppressed at 40 weeks of follow-up was 59%. Lower pVL and being non-nucleoside reverse transcriptase inhibitor (NNRTI)-naïve at initiation of mega-ART were predictors of a favorable virologic response. Baseline susceptibility to lamivudine, stavudine, didanosine, and saquinavir were also strongly associated with a favorable virologic response. In this series, 26 (25%) of patients experienced severe laboratory abnormalities or subjective adverse drug effects, of whom 6 discontinued treatment. Toxicity was considered frequent, but generally manageable, by the investigators.[10] Although these virologic data lead to some optimism about the value of this approach, there are inadequate long-term data to indicate whether the virologic response is translated into gains in survival or decreases in AIDS-related complications. There are no randomized controlled trials of mega-ART versus standard-ART and there are no studies evaluating the long-term clinical outcomes comparing these treatment strategies.

The second question addressed by the OPTIMA trial is that of the clinical utility of an anti-retroviral drug-free period (ARDFP). An anti-retroviral drug-free period as proposed in a person whose treatment has failed is different from a structured treatment interruption, i.e. cessation of anti-HIV therapy in the face of successful virologic suppression. For this trial, an ARDFP is a relatively brief period (i.e. 3 months) prior to reintroduction of treatment. When treatment is re-introduced, it is hoped that its benefits may exceed those that would have been achieved without an ARDFP. Declines in CD4 count and increases in pVL during the ARDFP are anticipated, but they are expected to be transient and reversible, without major long-term adverse consequences. All patients will receive optimal prophylaxis against opportunistic infections.

The potential value of an ARDFP, in the presence of multi-drug resistance, is not only the freedom from drug-related toxicity and improved quality of life, but also the possibility that the efficacy of a subsequent HAART regimen may be improved relative to such a regimen initiated without an interruption. At least one well-documented case evaluation demonstrates proof of principle for the concept but is it generalizable? [17] What is the optimal length of time for the ARDFP?

Further support for the concept of renewed treatment effectiveness following treatment interruption comes from a number of observational cohort studies.[14,18,19] There were 326 patients evaluated in the CHORUS cohort with advanced HIV who were failing treatment on their third or later multi-drug ARV regimen (median of 7 prior regimens) and who experienced treatment interruptions lasting a median of 108 days. Of these patients, 45% recovered 90% of their pre-interruption CD4 counts and had undetectable viral loads at five months after resuming HAART. This success rate was higher than would be expected after failing sequential multi-drug regimens.[19] Long-term follow-up of the cohort described by Deeks, et. al. revealed sustained viral load responses following treatment interruptions in patients who began regimens containing just one drug to which they were fully susceptible. This occurred despite evidence for persisting reservoirs of multi-drug resistant virus that was present before drug interruption.[20]

Evaluation of other cohorts has led some investigators to opposite conclusions. [21] Retrospective evaluation of the non-randomized longitudinal EuroSIDA cohort suggests an accelerated progression akin to the natural history of progressive HIV-associated immune deficiency disease for failing patients having increasing durations of treatment interruptions. Clearly, disease and death rates in patients who discontinue medical treatments (both anti-

HIV and anti-OI) are higher than in those patients who do not. This has been represented as relevant to outcomes of treatment interruption in future patients who wish to subsequently resume medical health maintenance.[22] However, treatment interruptions in this cohort were ad hoc and not linked to a strategy for resuming treatment designed to obtain favorable therapeutic outcomes.

Recent prospective studies have provided some support for this hypothesis. The first evaluated surrogate marker responses at 6 months from treatment interruption (or not), in 46 patients with mean CD4 T lymphocyte count between 300 and 400 cells/mcL. Plasma viremia increased 0.9 log10 from baseline in a 3-month treatment interruption, and CD4 count dropped 131 cells/mcL. Drug resistance mutations (by PCR of plasma viremia) decreased from 10.8 to 3.8 in (mean) number. On subsequent 5-drug rescue therapy, at 6 months follow-up, treatment interruption showed a greater mean increase in CD4 count (144 vs 80 cells/mcL), and greater proportion with suppression of viremia (47% vs 36% below 80 copies/mL). Clinical complications were not observed, and differences in these surrogate marker responses were not statistically significant.[3]

In the GIGHAART study, seventy patients with very low CD4 counts (20 to 30 cells/mcL) were randomized to a 2-month treatment interruption, and all received salvage therapy with 6-8 anti-HIV drugs (including hydroxyurea initially). Surrogate outcomes of plasma viremia and CD4 count were evaluated at 3 months from salvage treatment, and for as long as 6 months. Plasma viremia rose, and CD4 counts fell off treatment, but subsequent responses favored treatment interruption: By intent-to-treat analysis suppression of plasma viremia was achieved in 15% without, and 38% with treatment interruption at 3 months ($p \sim 0.05$). This difference subsequently declined by 6 months follow-up, but CD4 count

response was greater after treatment interruption, at +51 v +7 cells/mcL from baseline. Multi-drug treatment was tolerably maintained, with 2 /70 declining study treatment after randomization, and 5 /68 subsequently reducing treatment to fewer than 6 drugs. During the treatment interruption, 3 /34 in each group had any clinical adverse event reported. Clinical adverse events (including lactic acidosis, opportunistic infections, and 1 lymphoma death) occurred in 6 /34 v 10 /34, with more in the treatment interruption group. The primary endpoint for this trial was change in plasma viremia from baseline. Accrual was stopped for ethical reasons following a significant response in viral load that favored the treatment interruption arm. This study was not designed to determine the effect of treatment interruptions on major clinical outcomes.

The third randomized study was is CPCRA 064 (MDR-HIV) study, well summarized in the CPCRA/DAIDS memo of July 5th.[2] This prospective, randomized controlled study looked at a 4-month treatment interruption in 270 patients with an average baseline CD4 count 180 cells /mcL on clinical outcomes. Accrual had been slow. Outcomes were less frequent than predicted. Deaths were 8 in each group, and progressive disease was seen in 17 (treatment interruption) v. 5 (no treatment interruption). The most common was candidiasis (8 /22 events). Progression of disease (as defined in the CPCRA Data Collection Handbook) or death was observed in 34 patients, 22 treatment-interruption, and 12 without interruption (HR 2.6, 95%CI 1.2 – 5.5, p = 0.01). Events occurred both on (9 /34) and after (25 /34) treatment interruption. This study was closed based on futility of demonstrating a benefit to treatment interruption given accrual and outcome rates.

Each of these studies differs from OPTIMA in some elements of patient profile, intervention and outcomes. Surrogate marker responses may improve after a short treatment

interruption, given effective salvage therapy, but these may not be sustained. Tailored multi-drug salvage therapy seems safe and tolerable. Longer treatment interruption and fewer salvage options may require great attention to OI prophylaxis, due to the natural history of HIV immune disease. OPTIMA management has notified investigators of the disposal of the CPCRA MDR-HIV study, and emphasized the importance of OI prophylaxis during OPTIMA's 3-month treatment interruption.

Two developments would undermine the continued relevance of OPTIMA; the clinical condition of treatment failure could be solved or other research could definitively answer the questions posed by the study. The study might not be needed if a widely available cure for HIV were developed, although this is not anticipated in the foreseeable future.

A more realistic alternative would be if treatments were developed that had a low likelihood of failure, or if sufficient numbers of tolerable treatment alternatives were available so that effective options could not become exhausted. Indeed, new treatment regimens are becoming available that are easier to adhere to because of fewer side effects, lower pill burden, and more convenient dosage scheduling. New classes of drugs are being introduced whose effectiveness is less likely to be compromised by cross-resistance to regimens that have previously failed. [23, 24]

There has been a significant decrease in the number of patients eligible for OPTIMA that is partly due to the availability of new agents, particularly Kaletra and Tenofovir. However, the problem of salvage treatment for patients with multi-drug resistant infection is unlikely to become trivial despite these welcome trends. All anti-retroviral regimens are known to lead to resistance if not rigorously adhered to or if used as part of an inadequately

constructed combination. Significantly, increasing numbers of patients more recently randomized to OPTIMA have been failed by ART regimens including these new agents **(useful here to have #'s of prior kaletra, tenofovir subjects)**. Additionally, there is growing evidence for transmission of multi-drug resistant virus so that resistance testing is increasingly recommended for recent infection.[25] Also, the effort to increase treatment in impoverished countries is likely to amplify the problem of multi-drug resistance because of the inability to sustain and monitor fully effective therapies. These emerging trends in drug resistance are likely to increase with time so that the underlying rationale for OPTIMA, which seeks to find the best approach to salvage, will continue to be of relevance to an increasing number of people.

The rationale for OPTIMA would also be compromised if other studies convincingly answer the questions posed, which might make continuation of the randomization unethical. The GIGHAART study provides important support for the controversial concept that a treatment interruption can enhance response to subsequent anti-viral treatment. Its design, however, evaluates the limited surrogate outcome of virological response over a short period and does not adequately address the more meaningful impact of treatment interruptions on clinical endpoints. The MDR study was terminated for futility and in part also because of undesirable declines in CD4 lymphocytes and a trend toward increased HIV associated clinical events in the treatment interruption arm. Unfortunately, the majority of these clinical events appear to have been avoidable with assiduous prophylaxis. Neither of these trials has persuasively answered the clinical question of the effect of a drug free period on subsequent treatment in this population, and no randomized trials compare the risks and benefits of Standard-ART vs Mega-ART. Thus, the failure of other studies to answer the questions

posed by OPTIMA, coupled with evidence supporting one of the more controversial hypotheses, enhances the ongoing relevance of the trial.

V. Study Objectives

The study will compare different strategies for the management of patients with HIV infection for whom first and second line HAART has failed.

The OPTIMA study aims to evaluate (a) the effect of mega-ART compared to standard-ART and (b) the effect of an intended 3-month ARDFP compared to no ARDFP in the management of patients for whom previous HAART therapy has failed. The impact of mega-ART and ARDFP on cost and Quality Adjusted Life Years will be determined; lifetime costs and outcomes will be modeled to determine whether these strategies are cost-effective.

VI. Study Outcome Measures

The primary and secondary endpoints are:

Primary Endpoint: 1. Time to a new or recurrent AIDS event or Death

Secondary Endpoint: 1. Time to development of a new non-HIV related serious adverse event

Other outcomes that will be assessed are:

1. Quality of Life
2. Incidence of grade 3 or 4 clinical or laboratory adverse events
3. Changes in CD4 counts, viral load and resistance
4. Process measures including hematologic profiles, electrolytes, renal function, liver function, pancreatic function, and lipid levels)

VII. Study Design

The trial is a 2x2 open randomized study of patients with advanced HIV disease in whom ART, including all three classes of anti-HIV drugs, have failed. Randomization will allocate patients (a) either to a *ARDFP* of at least 3 month duration or to '*No ARDFP'* and (b) to either a '*mega-ART*" regimen (5 or more drugs) or to a '*Standard-ART'* regimen (up to 4 drugs). Patients will be followed until the last participant enrolled has completed 12 months of follow-up (a median of 3.5 years).

The primary hypothesis is that *mega-ART* is superior to *standard-ART* in terms of prolonging life and delaying the occurrence of new or recurrent AIDS events.

This is a management trial comparing different strategies for salvage therapy. The first comparison will be between the strategy that incorporates the use of at least 5 anti-HIV drugs (*mega-ART*) (e.g. 3 reverse transcriptase inhibitors, 1 non-nucleoside reverse transcriptase inhibitor and 2 protease inhibitors used in therapeutic doses) and a conventional strategy of up to 4 anti-HIV drugs (*Standard-ART*). For the purposes of this trial, dual protease combinations involving ritonavir for pharmacokinetic reasons (in doses of 100-200 mg bid) will be counted as one drug. Hydroxyurea is not regarded as an anti-retroviral drug for this study, but may be used at the investigator's discretion. The choice of anti-HIV drugs used during this trial will be at the discretion of the clinician and will be chosen according to the patient's previous drug history, history of drug intolerance and drug susceptibility assessment (determined by genotypic resistance testing).

The second comparison is between an intended '*3-month ARDFP'* and '*No ARDFP*'. The *ARDFP* is intended to be a minimum of 3 months in duration. There may be some patients who remain stable and feel well during the *ARDFP* and who may wish to extend the *ARDFP* duration. This will be permitted at the clinician's discretion as long as the 2 and 6-week safety assessments after the re-initiation of ART are performed.

Genotypic viral resistance testing will be performed on all subjects during the screening process, prior to randomization. The results will be recorded on a data form provided by the VIRCO laboratory and forwarded to the treating physician or center at the time of treatment initiation. A copy of this form will also be sent to the coordinating center in each country.

A central randomization list will be prepared for the whole trial with variable block size used for individual large sites or groups of smaller sites within each country and

stratified by screening CD4 cell count. However, each country will be responsible for its own randomization processes. Patients will be randomized by telephone randomization or fax through the Canadian HIV Trials Network Data Center, the VA Cooperative Studies Program Coordinating Center or the UK-MRC Clinical Trials Unit.

Patients will be randomized to either *mega-ART* or *standard-ART* and will start treatment either immediately or following a *3-month ARDFP*. For patients randomized to *ARDFP*, knowledge of their eventual drug allocation could influence the initial phase of the study. Consequently, for this group of patients, assignment to *standard*- or *mega-ART* will not be communicated by the data center until the time of drug reintroduction.

The variety of drugs, which might be used in the trial, is too large to allow for the use of a blinded treatment methodology. Objective end-points, especially suitable for open studies, will be used to measure the efficacy of outcomes. The primary end-point is the incidence of a new or recurrent AIDS-defining illness or death, and will be adjudicated by a blinded Endpoint Review Committee.

This is a clinical management trial comparing strategies involving *ARDFPs* and numerous drugs; as such, this trial is not intended to test the efficacy of individual drugs (none of which alone are likely to have considerable effects). In this context, the overriding need is for individualization rather than consistency of re-treatment regimens. Factors that influence and guide regimen decisions include treatment history, drug intolerance, available choices and their expected toxicities, and measures of HIV susceptibility. Although standardization of the process of selecting treatment regimens would be ideal across the trial, this will not be possible even though each clinician will likely be consistent in their

selection of Mega or Standard-ART regimens. Changes in treatment strategy will not be recommended prior to patients reaching a trial end-point.

This is an open randomized study using drugs that are available in routine care or through compassionate use programs or other open-label access to new HIV drugs. There is obviously a potential risk of poor compliance to the allocated strategy. For instance, patients allocated to ARDFP may resume drug therapy earlier than 3 months because of concern over disease progression and the 6-week visit for ARDFP patients could allow earlier re-initiation of ART in patients who experience a precipitous decline in CD4 counts. Similarly, patients on mega-ART may stop treatment because of presumed drug toxicity. Patient compliance and adherence to anti-HIV drug therapy is a universal problem in therapy and trials. It may be relatively more operative in this trial than in general, since the study population will be selected based on treatment failure. This is often due to past non-adherence to therapies, whether through non-compliance, or drug intolerance, or other factors that may still be present in individual cases. The best way to deal with adherence in good clinical practice is by planning and counseling, and either identifying prompts or eliminating obstacles to adherence. The same standard of good clinical practice will be followed for all study volunteers, regardless of allocation. The DSMB will closely monitor adherence to allocated regimens by treatment groups and advise the Steering Committee as necessary. Since there is no consensus (or guidelines) about the best treatment strategies for such patients, clinicians should not have great difficulty supporting patients to remain on their allocated treatment strategy.

VIII. Patient Population

The intent of the trial is to include patients, male and female, who have advanced HIV disease and in whom regimens that have included all three classes of drugs have failed.

Sixty-five enrolling sites (25 VA, 20 UK and 20 Canada) will participate. The overall anticipated accrual rate is 9-10 patients per month.

a. Inclusion Criteria:

Patients will be eligible for enrollment in OPTIMA if they have:

1) Ability to provide Informed Consent

2) Age of 18 years or more

3) Serologic or virologic diagnosis of HIV infection

4) Had failure* of at least two different multi-drug regimens that included drugs of all 3 classes that the patient can tolerate

Or

laboratory evidence of resistance** to drugs in each of the **3** classes

5) Had at least 3 months of current ART and is still on treatment (unless a new failure*, defined as (c) below)

6) Two most recent results (which can include screening) on current ART of :

CD4+ T-cell count ≤ 300 cells/mm^3 or ≤ 15%,

and plasma viral load ≥ 5,000 copies/ml (by Roche Amplicor, v1.0) or

≥ 2,500 copies/ml (by bDNA: Bayer v3.0/Chiron v3.0, or PCR: Roche Amplicor Monitor/COBAS v1.5)

*Failure (since availability of viral load tests) is defined as:

(a) failure to suppress plasma viral load after 24 weeks of therapy, or

(b) a rebound of at least 0.5 log_{10} in plasma viral load from nadir, or

(c) a less than 1.0 log_{10} drop in plasma viral load after at least 4 weeks continuous treatment with a current new multi-drug regimen

OR

(in the therapeutic era before viral load testing was available) failure is defined as: CD4 decline >50% from peak treatment response, or below pretreatment level, or clinical progression of HIV disease.

** Resistance (this could be from screening susceptibility test) is defined either as:

(a) genotypic (defined as the presence of primary mutations associated with resistance to at least 2 drugs in each class), OR

(b) phenotypic evidence of 3-class resistance

b. Exclusion Criteria:

1) Pregnancy, breast-feeding or planned pregnancy
2) Likelihood of poor protocol follow-up or if *Mega-ART* is not feasible (due to significant intolerance of many ART drugs)
3) Serious, uncontrolled major opportunistic infection (OI) within 14 days of screening
4) Likelihood of early death due to non HIV-disease

IX. Trial Procedures and Assessments

a. Screening

The following evaluations/procedures will be performed at the time of screening. Results from tests done within 30 days will be acceptable for screening.

1. Obtaining Informed Consent (see **Appendix 1**)
2. Demographic information
3. Plasma HIV RNA
4. CD4 and CD8 cell count and percentages (%)
5. Urine pregnancy test for women of childbearing potential
6. Blood collection for resistance testing and plasma storage

b. Baseline Evaluations

The following evaluations will be obtained for patients who, after screening, are considered to be eligible for entry into the study:

1. Review of medical conditions since screening
2. Medical history that includes the following:
    - any significant concurrent medical conditions
    - previous AIDS-defining events and current signs and symptoms of HIV disease
    - previous and current ART therapy
    - details of OI prophylaxis or maintenance prescribed for trial
3. Physical examination (including height, weight, blood pressure, umbilical waist measurement)
4. Hemoglobin, total white blood cell count, differential and platelet count
5. Biochemistry, consisting of creatinine, glucose, total bilirubin, alkaline phosphatase, AST and/or ALT, amylase and/or lipase, sodium, potassium, cholesterol (total, HDL and LDL), and triglycerides (fasting where possible)
6. Nadir (lowest) CD4 count before antiretroviral therapy (if available)
7. CD4 and CD8 cell counts and percentages (%)
8. Plasma HIV Viral load
9. 'On-study' ART; details of new ART regimens prescribed for the study
10. Hepatitis B and C status, Toxoplasma serology and, if available, Syphilis and Tuberculosis status

10. Plasma for storage

11. Peripheral Blood Mononuclear Cells (PBMCs) for storage (if available at site)

12. MOS-HIV, Health Utilities Index and EuroQoL (all patients), and U-Titer Program (US patients only)

c. Randomization

The site coordinator will review eligibility and complete the baseline evaluations and procedures. Then he/she will telephone or fax the coordinating center to obtain the randomization allocation. The assignment will then be confirmed by letter or fax.

d. Follow-up evaluations

After randomization (Time 0) **all** patients will have visits at weeks 6, 12, 24 and then every 12 weeks.

At 2 and 6 weeks after beginning ART in the study **all** patients will have 'safety' visits; the timing (since randomization) of these visits may vary from patient to patient depending on the length of the ARDFP period which is intended to be 3 months. The treating physician, in consultation with the patient, may consider prolonging or shortening the ARDFP as deemed clinically appropriate. All other visits throughout follow-up will be based on the time of randomization.

The following will be recorded on each visit or as specified in the table of trial assessments (Table A). The evaluations will be accepted if done within the specified window as shown in the table.

1. Medical history since last reported assessment including signs and symptoms of HIV disease, weight and umbilical waist measurement
2. Adverse events and their severity and considered relationship to the study drug
3. Hematology and Biochemistry as at baseline
4. CD4 and CD8 cell counts and percentages (%)
5. Plasma HIV RNA
6. Plasma and Peripheral Blood Mononuclear Cells (PBMCs) for storage
7. Results of drug Resistance testing (if done)
8. Changes of ART, OI and other concomitant medication
9. MOS-HIV, Health Utilities Index and EuroQoL (all patients) and U-Titer (U.S patients only) (at 6 weeks and 12 weeks, then every 12 weeks)

weekly visit schedule for a patient allocated to No ARDFP**

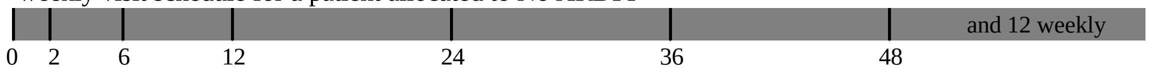

intended weekly visit schedule for a patient allocated to a 3 month ARDFP**

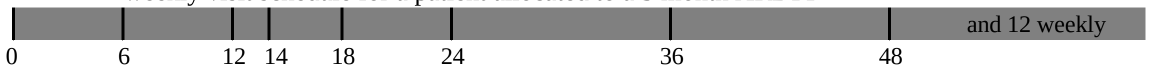

**ARDFP is antiretroviral drug free period

All patients will be seen by their primary physician and by the site coordinator at the pre-specified intervals. There will be a window for completing follow-up visits: +/- 1 week for WEEK 2 and 14 visits; +/- 2 weeks for the WEEK 6, 12 and 18 visits; and +/- 4 weeks for all other visits. In the event of inability to complete a scheduled clinic visit due to hospitalization, intercurrent illness, or logistical constraints, the patient or family will be contacted by phone. When necessary, the patient's primary care physician will be contacted

to provide necessary clinical information. The coordinating center in each country will provide each site with (a) reporting of missing forms, and, (b) an accounting of those forms that contain incomplete information on a regular basis.

Vital status and date of death of patients will be verified by regular follow-up or by using national HIV databases. AIDS-defining events will be assessed clinically and adjudicated using well-established evaluation guidelines (**Appendix 2**) by an independent Endpoints Review Committee that will be blinded to the patients' treatment allocation.

Site coordinators will follow each randomized patient at their study center; missed follow-up visits will be noted.

e. Assessment of Compliance

Responses to standardized questions will be reported at each follow-up visit to assess patient compliance with ART.

f. Reporting of Death, HIV Disease Progression, Adverse Events

If the patient has died, experienced any new or recurrent AIDS-defining illness or experienced an adverse event (serious, Grade 3 or 4, or one leading to a modification of ART; see **Appendix 3**) since the last visit, the investigator/study coordinator will complete specific case report forms.

Serious Adverse Events (SAE):

According to the ICH Harmonized Tripartite Guidelines for Clinical Safety Data Management: Definitions and Standards for Expedited Reporting (1994), (**Appendix 4**) a Serious Adverse Event (SAE) in the context of the OPTIMA trial will be defined as 'any untoward medical occurrence that:

1. results in death (where the adverse event occurs 'on study' while on ART or within 30 days of receiving ART)
2. is life-threatening (patient was actually at risk of death at the time of the event)
3. requires unplanned inpatient hospitalization or prolongation of existing hospitalization
4. results in persistent or significant disability/incapacity or
   is a congenital anomaly/ birth defect
5. any other important medical events considered serious by the investigator (e.g. intensive treatment in an emergency room or at home for allergic bronchospasm; blood dyscrasia or convulsions that do not result in hospitalization; or development of drug dependency or drug abuse)

All serious adverse events must be recorded on the Serious Adverse Event Form. All serious and unexpected adverse events must be reported promptly to the Data Coordinating Center. The Trial Management Committee will review overall event rates on a 3-monthly basis.

Data on SAEs will be reported, if required, to ethics committees or regulatory authorities (treatment assignment blinded) and will be tabulated and presented to the Data

Safety Monitoring Board (DSMB) (treatment assignment blinded or unblinded as per DSMB decision).

g. Schedule of Evaluations and Forms

Table A:

Case Report Form (CRF)	TIME OF CRF COMPLETION							
	Screening	Entry/ Baseline	Weeks after Randomization			Weeks after ART Initiation		At time of Event
			6 ± 2 *	12± 2	q12 ± 4	2 ± 1	6 ± 2 *	
**Informed Consent**	X							
**Screening** (***Urinary pregnancy test, viral load, CD4, CD8 and blood collected for resistance testing***)	X							
**Randomization Checklist**		X						
**Baseline History and Physical Examination**		X						
**ART Treatment History**		X						
**Laboratory Evaluations** (*viral load, CD4, CD8 and blood collected for storage*)		X	X	X	X	X	X	
**MOS-HIV**		X	X	X	X			
**Health Utilities Index**		X	X	X	X			
**EuroQoL**		X	X	X	X			
**U-Titer** (US-VA ONLY)**		X	X	X	X			
**First ART Regimen On-study**		X						
**OI and Concomitant Medications at Baseline**		X						

**Follow-up**			X	X	X		X	X		
**HIV Adherence Treatment Scale**			X	X	X		X	X		
**ART Medication Change**										X
**OI & Concomitant Medications Change**										X
**AIDS Event/Death**										X
**Adverse Events (Grade 3 or 4)**										X
**Serious Adverse Events**										X

** For patients randomized to No ARDFP, this is the same visit on the same date and all evaluations and forms should be complete as indicated under the "Weeks after Randomization" column.*

*** Computer application*

**** If the duration of the ARDFP is not 12 weeks and the 'End of ARDFP' visit does not coincide with the window for a regularly-scheduled visit, the Laboratory Evaluations and the follow-up assessment are performed.*

X. Treatment Regimens

a. Anti-retroviral Medications

All available antiretroviral medications can be used for treatment of patients in this study. **Appendix 5** summarizes dosages, drug interactions, contraindications and adverse events associated with each drug.

This is a clinical management trial comparing strategies involving ARDFPs and drug combinations, rather than testing the efficacy of individual drugs and therefore the overriding need is for individualization rather than consistency of treatment regimens. Factors used to select drug combinations for both mega-ART and standard-ART include treatment history, drug intolerance, available choices and their expected toxicities (**Appendix 5**), and measures of HIV susceptibility. While consistency or standardization of the process for selecting treatment regimens (standard-ART or mega-ART) would be

desirable, the complexities are such that no simple algorithm or guidelines can cover all circumstances nor is there universal agreement as to what approach should be taken. Baseline genotypic testing is incorporated in the study and individualization of the regimen is left to the treating physician.

The rapid process of new drug development challenges HIV treatment studies. However, it is unlikely in the face of multi-class drug resistance and cross-resistance within drug classes, that new drugs within existing classes (NRTI, PI, and NNRTI) will offer enough anti-retroviral activity to overcome existing virological and immunological failure.

Further, potential new classes of drugs include integrase inhibitors, which may be three years away from broad availability are not likely to be any more effective in rescue than currently available drugs that have failed. Likewise, T20, an injectable oligopeptide fusion inhibitor can produce over a ten-fold reduction in plasma viremia as monotherapy,[12] but resistance rapidly emerges. In addition, immune modulators and therapeutic vaccines may emerge as new treatment options. Nevertheless, if new classes of drugs become available during the course of this study, likely through compassionate access, their benefit will critically depend on the efficacy of concurrent treatment with existing agents.

In patients with multiple drug resistance, the optimum strategy determined by this study is likely to be the best strategy to be used with the new drug. In order to enhance the validity of this trial, the inclusion of new agents into the standard and mega-ART regimens as the agents become available will be encouraged.

b. Opportunistic Infections (OI) Prophylaxis

All patients should receive OI prophylaxis according to established guidelines (**Appendix 6**) . Declines in CD4 counts may appear at any time in patients in the trial, but can be anticipated in patients on an ARDFP, and therefore, appropriate action should be taken.

c. Treatment Failure/ Guidelines

For the purposes of this study, drug failure will be defined in accordance with current guidelines.

d. Maintenance of Allocated Treatment Strategy and Modification

If possible, investigators are urged to keep patients on their assigned strategy in the absence of a new or recurrent AIDS-defining event, or medical contraindications to their assigned therapy. However, if warranted, the study medications should be continued or discontinued according to established guidelines. Since patients are assigned to a strategy, individual drugs may be withdrawn and/or substituted without necessarily changing the allocated strategy.

e. Patient Follow-up

The aim is to follow every patient until death or study closure. However, investigators may withdraw patients from their assigned treatment strategy in the event of intercurrent illness, adverse events, withdrawal of consent, and other compelling clinical

conditions. Unnecessary withdrawal of patients from their assigned treatment strategy should be avoided.

Whenever possible patients should continue to be followed according to the study procedures even if they have withdrawn from allocated strategies.

XI Biostatistical Considerations

The sample size estimated for this study is based upon a number of assumptions about the expected rate of outcomes [on the 'standard-ART' arm for comparison with 'mega-ART' arm, and 'No anti-retroviral drug-free period' (No ARDFP) arm for comparison with 'anti-retroviral drug-free period' (ARDFP) arm], crossovers between groups, and losses to follow-up. Computations were made for a number of different scenarios detailed in **Appendix 7 (REVISE Appendix)**. Ultimately, the sample size was set to detect a difference between standard-ART and mega-ART of 63.2% versus 52.9% in the proportion of patients who, by the end of the study, would have had an event with a power of 80% and a two-tailed alpha of 0.05. The expected proportions factor in the expected crossover and

loss to follow-up rates. The study has the same power to detect the same difference between No ARDFP and ARDFP arms.

The primary comparisons in the OPTIMA Trial are between Standard-ART and Mega-ART, and ARDFP and No ARDFP. The analysis plan for the trial is described in **Appendix 8**.

In each country, data will be sent to the coordinating center, which will then perform data checks and generate data edits. Verified data from each country will be sent to the Canadian coordinating center on a regular basis so that the data from all three countries can be merged. Analysis tables will be generated both for trial monitoring purposes by the Trial Steering and Trial Management Committees, as well as for the Data Safety Monitoring Board (see **Appendix 9**).

XII. Study Administration

The management of the trial will be shared between the three countries. Each country will be responsible for the day-to-day management of the trial within its borders. This includes handling randomization, orienting and monitoring clinical sites, ensuring quality and timeliness of data and dealing with site-specific issues. Within each country, the trial will be managed by the Canadian HIV Trials Network, the MRC-UK Clinical Trials Unit and the VA Cooperative Studies Program Coordinating Center (CSPCC-West Haven).

a. Organizational Structure

The organizational structure and the functions of the various committees and groups of the OPTIMA Trial are summarized below. OPTIMA committees and groups will meet prior to the beginning of patient intake and as required.

i. **<u>Trial Steering Committee</u>**

The Trial Steering Committee (TSC) will provide general oversight of the trial conduct, accrual, retention, quality, data systems and management in accordance with the protocol and in light of reports or recommendations from any of the trial committees. The trial committees are: the Data Safety and Monitoring Board, the Endpoint Review Committee and the Trial Management Committee.

At regular intervals the TSC will also review and consider relevant information or developments in the field of HIV therapy and in legal, statutory, or regulatory requirements.

The TSC will maintain regular contact with the 3 national trial organizations and will provide a forum for the exchange of information about the trial. The TSC will meet twice a year, with additional meetings and teleconferences arranged when necessary. All decisions will be made by a simple majority vote. In the event of a no majority vote, the Chairperson for the meeting will have a deciding vote.

<u>Membership of the TSC:</u>

- Independent Chair
- Three joint Co-Chairs from participating countries
- Independent TSC Members

  HIV Clinician, Health Economist, Virologist
- Community Members, 1 from each country

- One OPTIMA Clinical PI from each country on a rotating (or locally decided) basis
- Representatives of the National Trial Organizations
- One representative of each Trial Management Committee Subgroup (see below) on a rotating basis or as agreed by the subgroup

ii. **Trial Management Committee**

A Trial Management Committee (TMC) will oversee the day-to-day management of the trial, resolving questions about eligibility, enrollment, randomization, regimen determination, length of drug free periods, grading and disposition of toxicities and adverse events, and determination of endpoint questions that need resolution by the ERC. Leadership will consist of the 5 Clinical PIs, chairing in rotation. Each subgroup will be represented on the TMC with at least one member from each country. The TMC will operate by consensus, not by majority vote.

TMC Membership:

- **Clinical Subgroup** (*OPTIMA PIs*)
- **Health Economics Subgroup** (*OPTIMA Health Economists*)
- **Statistics and Data Management Subgroup** (*OPTIMA Biostatisticians and Information Technology Expert*)

  The Statistics and Data Management Subgroup will advise on protocol development, determine database structure, and develop data management and data capture procedures. The Canadian statistics/data management team will perform the 3-country data merging functions.

- **Sub studies Subgroup** ***(Representatives of each of the following 3 groups: OPTIMA Clinicians, OPTIMA Biostatisticians, OPTIMA Health Economists, OPTIMA Field Investigators; and an ad-hoc member)***

  The Sub studies Subgroup will determine the mechanisms for sub study concepts to be reviewed and approved, and will make recommendations to the TSC for prioritization of sub studies.

- **Public Relations and Publications Subgroup** *(Representatives of each of the other subgroups plus a co-chair)*

  The Public Relations and Publications Subgroup will manage and coordinate publicity, presentations and preparation of reports. All reports, abstracts, or other public disclosures on the trial prepared by clinical investigators, principal investigators, or any of the 3 national trial organizations will be assigned or reviewed by the PRP group, which will report to the TSC.

- **Endpoint Review Committee**

  The Endpoint Review Committee (ERC) will determine the validity of potential endpoints that fail to meet standard criteria, as defined by the protocol. The TMC will forward such potential endpoints to the ERC for review. Two members, who are not from the country where the potential endpoint occurred, will review and make the determination on such an endpoint.

  <u>ERC Membership:</u>

  - 1 Independent Chairperson
  - 1 Independent HIV clinician

- Clinical Subgroup of the TMC

iii. **Data and Safety Monitoring Board**

The Data and Safety Monitoring Board (DSMB) will monitor all aspects of the trial. In strict confidence, it will review data on enrollment, adherence, activity, and toxicity by treatment allocation, meeting in open and closed sessions every 6 months. For the primary endpoint analysis the Haybittle-Peto method for monitoring will be used with a nominal p-value of 0.001 for each interim look and p-value of 0.05 for the final analysis.

The DSMB members will be completely independent of the trial and will advise the TSC if any interim results or information from other sources indicate that the trial should be modified or discontinued.

The Trial Biostatisticians will provide information and statistical reports for meetings of the DSMB and will attend meetings to present reports and answer any queries the DSMB may have. Representatives of the TSC will also attend the open introductory sessions of meetings when appropriate. The DSMB will receive minutes of each meeting of the TSC.

DSMB Membership:

- Statisticians
- Clinicians
- Clinical Trialists

iv. **Secretariat**

A Secretariat will be appointed which will reside outside of the various committee structures. The Secretariat will relate to all of the committees and will serve as the main coordinator of trial committee activities, including planning of meetings and teleconferences, development of meeting and teleconference agenda and minutes, and maintenance of committee memberships. The functions of the Secretariat may be shared between 2 or 3 countries, as needed.

The UK will perform Secretariat functions for the DSMB and Trial Steering Committees; the US will perform Secretariat functions for the Trial Management Committee and Endpoints Review Committee.

Note: For the VA only. The Human Rights Committee at the Coordinating Center will review the study annually to ensure proper protection of patients' rights and safety. A report about the progress of the study and ethical issues relevant to these bodies will be presented at regular intervals. In the interim, these bodies may be asked to convene if there is any serious adverse event requiring its attention. Members of these bodies could attend each meeting of the DSMB and will site-visit enrolling sites annually to determine that the patients' rights and safety are being properly protected.

b. Monitoring Patient Intake/Probation, Termination of Participating Sites

The TMC and TSC will monitor the intake rate and operational aspects of this study. Participating medical centers will continue to recruit in the study only if adequate patient intake is maintained. Actions leading to discontinuation of patient enrollment at a center will only be taken by the TSC with the concurrence of the DSMB and the leadership of the three participating agencies.

If recruitment is not proceeding at an appropriate rate, the TSC will scrutinize the reasons. Based on this information this committee may choose, with the approval of the Data and Safety Monitoring Board (and Director/Chief of agency-for VA only), to stop further patient enrollment at existing centers, add additional centers, or make modifications to the inclusion/exclusion criteria. In the VA, participating sites (except for small centers where accrual rate is anticipated to be slower) that do not enroll at least 1 patient in 3 months will be given an opportunity to improve within a reasonable period. If there is no improvement in accrual after the probation period, the site may be subject to reduced funding or possible termination as a study site.

c. Monitoring Clinical Site Performance

Strict adherence to the protocol will be expected of every participating center and monitored by the TMC, the TSC and the DSMB. Documentation of protocol breaches will be required and any site with repeated major protocol violations may be considered for termination. If a participating site investigator feels that adherence to the protocol will in any way be detrimental to a particular subject's health or well-being, the interest of the patient must take precedence. By agreeing to participate in this study, the clinical site delegates responsibility for global monitoring of the ongoing study to the TSC, the DSMB and the Coordinating Center (and the Human Rights Committee for VA). However, local committees and subcommittees involved in research at the enrolling medical centers may require the participating investigators to submit annual reports concerning the status of the study at the medical center for local monitoring purposes.

d. Site Visits

Site visits will not be performed on a pre-determined and regular basis. However, guidelines for site monitoring exist for each country and are described in the Good Clinical Practice section of the Trial Operations Manual.

e. Data Safety Monitoring

The OPTIMA Trial will be overseen by an independent DSMB, which in addition to seeing that the trial is being conducted in the manner proposed, will be responsible for overseeing the ethical conduct of the study.

At each of its meetings during the study period, the DSMB will review the recruitment rate and assess the difference between the actual and the projected rate, as well as the impact of these assessments on overall trial size. If accrual is inadequate, inclusion and exclusion criteria may be scrutinized and revised if appropriate. An assessment of whether the trial should be continued will be made followed by a recommendation, as appropriate. Serious adverse events will be reported to the DSMB.

Since this is a 2X2 factorial design, there is a possibility that one or both of the treatment comparisons could be stopped early for efficacy and/or safety reasons. If one treatment comparison is stopped early, continuation or termination of the other comparison would be a consideration.

At each meeting of the DSMB, the accumulating information on endpoints will be reviewed. At its first meeting, the DSMB will decide on the method they will use to adjust for the interim look at the primary endpoints (after all the patients have been followed for six months).

f. Endpoint Review

The Endpoint Review Committee (ERC) will meet on a regular basis to adjudicate endpoints, blinded to treatment allocation. A Table of Presumptive and Definitive Criteria for AIDS events provided in **Appendix 2** will assist in adjudication of endpoints.

The ERC will determine the validity of potential endpoints that fail to meet standard criteria, as defined by the protocol. The Trial Management Committee will forward such potential endpoints to the ERC for review. The ERC consists of 7 individuals (an independent chair, an independent member and the five OPTIMA clinical PIs). All seven members review all events (exception: events that occur at the clinical PI's participating center are reviewed by the other 6 members only) and the final arbitration is done by majority vote. ERC Independent Chair is final arbiter in case of a irresolvable disagreement among ERC members.

XIII. Publications

a. Publication Policy

The TMC will develop guidelines for the preparation of papers (abstracts) for presentation at national and international meetings, as well as the preparation of manuscripts for peer-reviewed publication. Any publication or presentation during the active phase of the study must have prior approval of the TMC and TSC.

All publications are to be approved by the TMC and TSC before submission for publication. The TMC will develop guidelines to protect patient confidentiality, to prevent unwarranted release of study information, and to prevent conflict of interest. The TMC and TSC will resolve problems of authorship and maintain the quality of publications. All publications will acknowledge appropriate funding sources.

The data derived from this clinical trial are considered the property of the Tri-National Clinical Trials Research Initiative (jointly owned by VA/UK MRC/CIHR) not the property of the individual participating investigator or health care facility where the data were generated.

The presentation or publication of any data collected by the participating investigators on patients entered into the OPTIMA study is under the direct control of the study's TMC and TSC. This is true whether the publication or presentation is concerned with the results of principal undertaking or is associated with the study in some other way. Although individual participating investigators will not have any inherent right to perform analyses or interpretations or to make public presentations or seek publication of any of the data other than under the auspices of and with the approval of the TMC and TSC, they will be encouraged to develop substudies or propose analyses subject to the approval by the TSC and TMC.

Outcome data by treatment group will not be revealed to the participating investigators until the data collection phase of the study has been completed. This policy safeguards against possible biases affecting the data collection. The DSMB (and the Human Rights Committee in the VA) will be monitoring the outcome results and may recommend

that the study be stopped for safety reasons or if a definitive answer is reached earlier than the scheduled end of the study.

XIV. SUB-STUDIES

Ancillary Studies (or "sub-studies") may divert resources from or interfere with the conduct of the primary study. However, since this study may provide a unique opportunity to examine other questions of scientific interest, investigators will be encouraged to undertake substudies and sub-analyses with the approval of the TMC and TSC.

The following guidelines are proposed for reviewing studies related to the OPTIMA Trial.

1. Any study specifically involving study patients will be considered an ancillary study, even if it is limited to one site. Ancillary studies will normally involve collection of additional data. Many "ancillary" analyses of currently collected data are already planned as part of the primary study.

2. Requests to perform ancillary studies will be accepted only from Participating Investigators at any of the designated study sites. Requests should be submitted initially as a letter of intent addressed to the TMC. The letter should specify goal and general design of the proposed research, the proposed number of subjects and study sites, and an estimate of the funding, if any, that will be required.

3. Letters may be submitted at any time, but the additional workload from a proposed sub-study should be carefully considered in order to ensure that recruitment in the primary study is not hindered.

4. Letters of intent will be reviewed by the TMC. Although the scientific merit of the study will be considered, the primary purpose of this initial review is to establish that the proposal in no way conflicts with the conduct of the primary study. Recruitment success and the overall performance of the proponent's site (and any proposed collaborating sites) will be one of the factors considered in this review. Sites that are struggling to meet recruitment goals for the primary study may be considered poor candidates for ancillary studies.

5. If the proposal is acceptable, the proponents will be asked to submit a formal study protocol (including a human consent form) and a budget. This proposal should be 4-6 pages in length, including references, and be in a format appropriate for submission to a research committee.

6. The protocol will be reviewed by the TMC and possibly by one or two additional reviewers with expertise in the area of interest. The purpose of this review is to determine the scientific merit of the study and to determine if the proposed ancillary study conflicts with the goals and/or conduct of the primary study. Depending on the nature of the ancillary study, it may require approval by the DSMB, (and HRC in the VA), local IRB and the three funding agencies. Based on the required reviews, proposals will either be approved or disapproved.

7. Any analysis which needs unblinding of treatment groups can ONLY be undertaken and published before results of the trial are reported if the TMC, TSC and DSMB agree.

8. Locating funding for approved studies is the responsibility of the proponents.

Any publications (including abstracts) resulting from ancillary studies must conform to publication policies of the three involved agencies, as specifically outlined for OPTIMA. This includes statistical review by the three Coordinating Center and adherence to authorship policies. The proponent of the ancillary study will normally serve as principal author of any resulting manuscripts.

XV. References

1. Katlama C, Dominguez S, Duvivier C, et al. GIGHAART Study Benefits of Treatment Interruption (TI) in Patients with Multiple Therapy Failures, CD4 cells <200 /mm3 and HIV RNA >50 000 cp/ml (GIGHAART ANRS 097). XIV International AIDS Conference, July 7-12, 2002, Barcelona, Spain

2. CPCRA 064 MDR-HIV Study. July 5, 2002, Memo to participating clinicians from trial management and sponsor (DAIDS) detailing reasons for closure of study due to futility.

3. Ruiz L, et al. Virological and immunological benefit of a salvage therapy that includes Kaletra plus fortovase preceded or not by antiretroviral therapy interruption in advanced HIV-infected patients (6-month follow-up). 9th Conference on Retroviruses and Opportunistic Infections, Seattle, Washington, USA, 2002. Abstract 422-W.

4. Hogg RS, Yip B, Kully C, Craib KJ, et al. Improved survival among HIV-infected individuals after initiation of triple-drug anti-retroviral regimens. CMAJ. 1999;160(5):659-65.

5. Palella FJ, Delaney KM, Moorman AC, et al. Declining morbidity and mortality among patients with advanced human immunodeficiency virus infection. N Engl J Med. 1998;338:853-60.

6. Mocroft A,Vella S, Benfield TL, et al. Changing patterns of mortality across Europe in patients infected with HIV-1.Lancet. 1998;352:1725-30.

7. Ledergerber B, Egger M, Opravil M, et. al. Clinical progression and virological failure on highly active antiretroviral therapy in HIV-1 patients: a prospective cohort study. Lancet 1999;353:863-68.

8. Skowron G, Holodniy M, Wesley M, et al. Nucleoside analogue associated mutations correlate with viral load response to lamivudine (3TC) in 150 nucleoside analogue-experienced patients. Fourth International Congress on Drug Therapy in HIV Infections, Glasgow, Scotland, Abstract P4. AIDS 1998;12(Suppl 4):S21.

9. Race E, Dam E, Obry V, et al. Analysis of HIV phenotypic cross-resistance to protease inhibitors in patients failing on combination therapies. 6th Conference on Retroviruses and Opportunistic Infections; January 31-February 4, 1999; Chicago, Ill., USA. Abstract 119.

10. Montaner JSG, Harrigan RP, Jahnke N. et al. Multiple drug rescue therapy for HIV-infected individuals with prior virologic failure to multiple regimens. AIDS 2001; 15: 61-69

11. Deeks SG and Martin JN. Reassessing the goal of antiretroviral therapy in the heavily pre-treated HIV-infected patient. (Editorial Comment) AIDS 2001; 14:117-119.

12. Deeks SG, Wrin T, Leigler T, et al. Virologic and Immunologic consequences of discontinuing combination anti-retroviral drug therapy in HIV-infected patients with detectable vireamia. NEJM 2001; 344 (7):472.

13. Frenkel LM and Mullins JI. Should patients with Drug-Resistant HIV-1 Continue to Receive Antiretroviral Therapy? (Editorial) NEJM 2001;344(7):520.

14. Youle M, Mocroft A, Johnsson M, et al. Surrogate marker responses to multidrug combinations comprising hydroxyurea, efavirenz, double protease inhibitor (PI) and nucleoside analogues in PI failures. Program and abstracts of the Second International Workshop on Salvage Therapy for HIV Infection; May 19-21, 1999; Toronto, Ontario, Canada. Abstract 18.

15. Report of NIH Panel to Define Principles of Therapy of HIV Infection. MMWR Recommendations and Reports 1998, 47, No. RR-5 Apr. 24.

16. Staszewski S, Gute P, Carlebach A, et al. Virological and immunological response to mega-HAART salvage therapy after failure of multiple anti-retroviral regimens]. Abstracts of the Fourth International Congress on Drug Therapy for HIV Infection; May 19-21, 1999; Toronto, Ontario, Canada. Abstract p94.

17. Walter H, Low P, Harrer T, et al. No evidence for persistence of multi-drug resistant viral strains after a 7-month treatment interruption in an HIV-1 infected individual. J Acquir Immune Defic Syndr 2002; 31(2):137-146).

18. Miller V, Rottmann C, Hertogs K, et al: Mega-HAART, resistance and STIs. Program and abstracts of the Second International Workshop on Salvage Therapy for HIV Infection; May 19-21, 1999; Toronto, Ontario, Canada. Abstract 30.

19. Braun JF, Fusco JS, Justice AC, et al for the CHORUS Project, Predictors of virologic and immunologic success post treatment interruption (TI) in patients with chronic HIV infection. 41st Interscience Conference on Antimicrobial Agents and Chemotherapy (ICAAC) Chicago, IL, USA, September 22-25, 2001

20. Deeks SG, Grant RM, Wrin T, et al. Persistence of drug-resistant HIV-1 after a structured treatment interruption and its impact on treatment response. AIDS 2003; 17(3):361-70

21. Miller V, Phillips AN, Clotet B, et al. Association of virus load, CD4 cell count, and treatment with clinical progression in human immunodeficiency virus-infected patients with very low CD4 cell counts. J Infect Dis 2002; 186:189-197

22. Lundgren JD, Vella S, Paddam L, et al. Interruption/Stopping Antiretroviral Therapy and the Risk of Clinical Disease: Results from the EuroSIDA Study. 9th Conference on Retroviruses and Opportunistic Infections, February 24-28, 2002, Seattle Washington, USA. Abstract #48.

23. Sanne I, Piliero P, Squires K, et al AI424-007 Clinical Trial Group. Results of a Phase 2 Clinical Trial at 48 Weeks (AI424-007): A Dose-Ranging, Safety, and Efficacy Comparative Trial of Atazanavir at Three Doses in Combination with Didanosine and Stavudine in Antiretroviral-Naive Subjects. J Acquir Immune Defic Syndr 2003;32(1):18-29.

24. Jiang S, Zhao Q, Debnath. Peptide and non-peptide HIV fusion inhibitors. Curr Pharm Des 2002;8(8):563-80

25. Ristig MB, Arens MQ, Kennedy M, et al. Increasing prevalence of resistance mutations in antiretroviral-naive individuals with established HIV-1 infection from 1996-2001 in St. Louis. HIV Clin Trials 2002; 3:155-160

XVI. Health Economics Component

a. Health Economics Evaluation

The health economics analysis will evaluate the cost effectiveness of the following strategies for managing the care of patients for whom previous standard-ART therapy has failed:

- Mega-ART (compared to standard-ART)
- An antiretroviral drug free period (ARDFP) followed by standard ART or Mega-ART (compared to No ARDFP)

These strategies will be evaluated using standard methods for cost-effectiveness analysis. The strategies must be evaluated relative to each other, to rule out any therapy that is dominated by another (e.g., strategies that are more costly and less effective than available alternatives). For strategies that are not dominated, an incremental cost-effectiveness ratio will be determined. Stochastic analysis will be undertaken using cost-effectiveness acceptability curves to express the uncertainty around cost-effectiveness based on mean costs and outcomes.[1,2,3]

b. Methods

The cost-effectiveness of the interventions will be estimated using the patient's life span as the time horizon for the analysis. Standard methods will be used.[4,5] The societal perspective will be adopted so that all costs, including patient-incurred costs, as well as the direct costs of health care costs will be considered. The cost of each resource will be estimated using unit costs and practices appropriate to each country.

Outcomes will be expressed in Quality-Adjusted Life Years (QALYs). In the primary analysis, quality adjustments will be based on periodic measures of utilities using the Health Utilities Index (HUI) and the EQ-5D, standardized multi-attribute utility instruments that will be used in all countries. We will also assess utilities of a random sample of U.S. participants will using a computer-based interview that uses time-tradeoff and standard gamble approaches to measuring utility. Decision analytic modeling will extrapolate from trial-based results to project lifetime health care cost and QALYs for each treatment group.

Sites in all three countries will gather a common set of utilization and outcomes data. A single model will be developed to find trial participants' lifetime cost and outcomes. These common data sources will be used to create a separate economic analysis for each country. Each analysis will reflect the country's guidelines for accepted practices for performing for cost-effectiveness studies, sources of costs for pharmaceuticals and health services, factors affecting lifetime survival, and discount rate. In addition to country specific analysis, we will employ statistical methods to investigate the extent to which cost-effectiveness differs between countries[6].

Costs will be expressed in current year dollars. In the U.S., costs will be adjusted by the Consumer Price Index or other appropriate indexes for all urban consumers. In the U.K.,

the Health Service Cost Index, published by the NHS Executive, Leeds, U.K. will be used to adjust for costs of health care services and a range of other indices, which would cover other items (e.g., wages). In Canada, appropriate indexes will be used to adjust for costs of health care and other services. Cost and outcomes will be discounted. For the country-specific study, each country will use its own standard discount rate. For the joint study, we will present cost-effectiveness results using a range of alternative rates.

c. Sample Size Considerations*:*

The proposed pharmacoeconomic component of this trial can easily accommodate changes in sample size and duration of follow-up . To the extent that estimates of cost-effectiveness will be based on modeling the event rates and disease progression observed during the trial to extrapolate survival over the patients' life time, increased follow-up time will provide a longer observation period upon which to model future events. With fewer patients in the study, the precision of the cost and outcome measurements will be affected. However, the trial is not powered according to cost and quality of life measures but rather according to clinical events. The uncertainty associated with cost-effectiveness results will be handled using methodology that is different from what is typically adapted for efficacy estimates emanating from randomized clinical trials. Specifically, we will adopt a Bayesian approach by reporting the probability that one treatment is the most cost-effective given the data rather than using classical hypothesis tests. We will implement this by estimating cost-effectiveness acceptability curves that show the probability that each management option is the most cost-effective conditional on the value the decision-maker places on additional health outcomes. We feel that this methodology will adequately handle a reduction in

sample size by showing the implications of the reduced precision in cost and outcome estimates for the decision uncertainty faced by decision makers.

d. Estimating Indirect Health-Care costs

Cost-effectiveness analysis that adopts the perspective of society requires consideration of costs borne by patients. These costs include the cost of securing medical care, such as travel expense and lost time from work or leisure activities.

The baseline survey form will obtain information on the distance the participant ordinarily travels to receive care. Travel expenses will be estimated as the product of distance traveled and the per-mile travel expense for private automobiles. In the U.S., we will use the rate allowed as a tax-deduction by the U.S. Internal Revenue Service. In the U.K., we will use the rate published by the U.K. Automobile Association. In Canada, the standard cost per kilometer will be used for estimating patients' travel expenses.

We will also estimate the time spent in obtaining health care, using a count of visits and days of hospital stay. This time will be valued using mean national wage rates adjusted by age and gender. For retirees, unemployed, and disabled, we will select an appropriate measure of the value of time to reflect their average opportunity cost.

Information about each patient's caregiver, such as their usual activities, will be collected at the baseline interview. The number of days caregivers have been taken away from their usual occupation due to patients' illness between two follow-up visits will be collected. The average market payment rate for home health aides performing chore services will be used as the unit cost for caregiver.

e. Estimating Direct Health-Care Costs

Key health care resource measures will be obtained—hospital care, medication, outpatient visits, and long-term care. This set of core resource use data will be collected in all countries. Additional database available in each country may be used for validation. For each hospital stay, we will determine the length of stay and the number of days of stay spent in intensive care. For each medication taken by patients, we will determine the medication name, the total daily dose, and the number of days it was taken. For outpatient visits, we will identify the type of practitioner visited, and whether the visit involved a therapeutic procedure. For long-term and rehabilitation care in an institution, we will determine the length of stay. Table B provides an overview of the three-country costing methods.

Table B. Utilization and Cost Measuring Method

Resource Category	Source of Utilization Data for All Countries	Source of Costs		
		**Canada**	**U.K.**	**U.S.**
Hospital inpatient care	Trial case-report form for length of stay, diagnosis.	St. Paul's Hospital Cost Model.	U.K. NHS Hospital returns (The Chartered Institute of Public Finance and Accountancy (CIPFA), 1999)<br>Specific costing studies in U.K. centers.	National cost estimate based on analysis of CDR, PTF, and Medicare relative values for VA services.<br>Medicare Reimbursement Rates for non-VA services.
Outpatient physician services	Trial case-report form	Provincial fee schedules	U.K. NHS Hospital returns (The Chartered Institute of Public Finance and Accountancy (CIPFA), 1999)<br>Specific costing studies in U.K. centers.	National cost estimate based on analysis of CDR, OPC, RBRVS relative values for VA services.<br>Medicare physician fee schedule for non-VA services.
Medication	Trial case-report form and baseline survey, updated with periodic survey of medication changes.	Average daily dose from treatment guidelines, manufacturers' list price	British National Formulary (British National Formulary, 2000)<br>Pharmacy costs based on specific costing studies in U.K. centers.	National Pharmacy Benefits Management Program; alternate based on Red Book average wholesale price.
Other inpatient care (e.g. nursing home care, rehabilitation and mental health care)	Trial case-report form and baseline survey, updated with periodic survey of medication changes.	St. Paul's Hospital Cost Model.	U.K. hospital reference costs (NHS Executive, 1999)<br>Specific costing studies in U.K. centers.	National cost estimate based on analysis of CDR, PTF, and Medicare relative values for VA services.<br>Medicare Reimbursement Rates for non-VA services.

Other outpatient care (e.g., community clinics)	Trial case-report form and baseline survey, updated with periodic survey of medication changes.	St. Paul's Hospital Cost Model.	National statistics (Netten A, Dennett J, Knight J. *Unit Costs of Health and Social Care*. Canterbury: PSSRU, University of Kent, 1999.<br><br>and specific costing studies in U.K. centers.	National cost estimate based on analysis of CDR, OPC, RBRVS relative values for VA services.<br>Medicare physician fee schedule for non-VA services.
Over the counter prescriptions	Baseline survey, updated with periodic survey of medication changes.	Canada retail price	U.K. retail prices	U.S. retail price
Patient transportation cost	Baseline survey	Statistics Canada schedule of average earning	Standard cost per unit distance based on national statistics (e.g. car travel from U.K. Automobile Association)	Rate allowed as tax deduction by the U.S. Internal Revenue Service
Patient productivity costs	Baseline survey	Market prices or rental rates	**U.K. average wage (Office for National Statistics, 2000)**	**Average cost per day from survey**
Formal caregiver costs	Baseline survey	Rates from Victorian Order of Nurses	**Survey of U.K. wage rates for appropriate staff**	**Average wage of U.S. formal caregivers**
Informal caregiver costs	Baseline survey	Average cost per day from the survey	**Use of wage rate for formal caregivers as a proxy**	**Average wage of U.S. informal caregivers**

CDR = cost distribution report; PTF = patient treatment file; OPC = outpatient care file; RBRVS = resource-based relative value scale.

U.S. health care cost methods

We have designed a cost-finding method that takes advantage of the available data and complies with guidelines for cost-effectiveness research. The U.S. Public Health Task Force panel on cost-effectiveness analysis recommends that both micro-cost and gross-cost methods be used to estimate health care costs.[7] The more labor-intensive micro-cost method is reserved for determining the cost of the care that is central to the analysis; gross costing is used when a less precise estimate can be used. This same hybrid method has been recommended to conduct cost-effectiveness research in the VA health care system.[8]

We will use two independent methods of finding VA health care costs. The primary set of VA cost estimates will be derived using national unit costs. Micro-cost methods will be used to find the cost of pharmaceuticals, and a gross-cost method to find the cost of other health care utilization. The micro-cost method will use data on every prescription received by trial participants obtained from the Pharmacy Benefits Management database. The cost of other care will be found by obtaining detailed utilization data from the centralized VA databases and estimating unit costs using gross-costing methods based on department level costs in the Cost Distribution Report (CDR), as described below.

The second set of estimates will use site-specific unit costs. The cost of VA health services will be extracted from the Decision Support System (DSS). DSS is a detailed cost allocation system that has been implemented throughout VA. Although DSS is a sophisticated tool that taps VA cost and utilization databases, it has not been previously used by researchers, nor has its cost estimates been independently validated. Because it is newly implemented, its accuracy is uncertain.[8] For this reason, we will use DSS as a secondary source of cost and utilization data.

Micro-Costing—Pharmacy Cost. We will obtain information on the use of prescription drugs from the VA National Pharmacy Benefits Management data. This is a new data source that now covers all VA health care systems and medical centers. We will request all data for trial participants from the National Pharmacy Benefits Management Program, in Hines, IL, including the name of the drug, dose, count of pills, the cost to the VA, and the NDC code. We will obtain the cost of each prescription from the national VA pharmacy database, and add an appropriate dispensing fee that reflects labor and supply costs for the source of the prescription, either the VA study sites or the VA centralized mail order pharmacy. We will develop an alternate cost of prescriptions based on the average wholesale price in the Medical Economics Red Book. This will allow us to prepare an estimate that reflects the societal cost for prescription drugs, as VA pays less for drugs than other U.S. health care payers.

Data on VA health care utilization. We will use centralized databases for information on the health care used by trial participants. From the database of VA hospital stays, we will obtain the Patient Treatment File (PTF), the date of discharge, days of stay in each ward (bed section), including the number of days in intensive care, and the ICD-9 diagnoses (up to 10 codes) assigned to the stay. Then we will obtain the outpatient care file (OPC), the date of the visit, the location of care (stop code), and CPT codes assigned to the visit, and the type of provider from the database of VA outpatient visits.

Gross-costing method of finding unit costs of VA health care utilization. The cost of VA health care encounters can be calculated by combining cost and utilization data from centralized VA databases with non-VA estimates of the relative cost of treating different diagnoses.[10] Such a method is needed because the VA does not routinely prepare

patient bills; consequently, it does not have the detailed charge data that researchers in the rest of the U.S. health care sector use to estimate costs.

Because VA does not have the detailed charge data to estimated costs, information from non-VA sources will be used to estimate the *relative* cost of each VA health care encounter. Relative costs estimated by non-VA sources provide a set of relative weights of resources used by VA health care encounters. The relative cost will be adjusted with data from the Cost Distribution Report to estimate the *actual* cost of each encounter. We will create a national average cost, based on costs reported by all facilities. To find the cost of acute hospital care, we will determine relative values for acute inpatient hospital stays by estimating the relationship between cost-adjusted charges, diagnosis, and length of stay in HCUP and Medicare discharge data. Long-term care costs will reflect the relative values from the assessments used to assign patients to Resource Utilization Groups. The cost of mental health, substance abuse, and rehabilitation care will be based on the average daily cost. The cost of outpatient visits will be based on the relative values that the Medicare resource-based relative value scale (RBRVS) reimbursement system assigns to the Current Procedures and Terminology (CPT) codes. We will determine the national average VA cost per relative value unit for each type of VA clinic, and use this value to estimate cost of outpatient visits.

Decision Support System (DSS). We will obtain information on the cost of all VA health care received by each trial participant from the national extract files from the Decision Support System (DSS). This system includes the cost of all inpatient and outpatient cares, including the cost of outpatient pharmacy. The national extract files do not provide detailed information on the specific cost of each type of pharmacy utilization; for

this reason, we will seek permission from each site to extract data on the outpatient prescriptions filled for each participant, as well as its cost.

Cost of non-VA care. We will determine the cost of care that participants incur outside of the VA using provider and payer databases, and participant interviews. Follow-up interviews will include questions about care received from non-VA providers. Participants will be asked to identify all non-VA hospital stays, long-term care stays, and the hospital where they received care. Participants will be asked for the number of outpatient visits they have made since the last follow-up interview.

We will extract the cost of care provided under contract to VA from the Fee Basis files. Individuals who are 65 years old or disabled are eligible for health care from Medicare. We will estimate the cost of Medicare services used by participants using claims data from the Health Care Financing Administration. We hope to take advantage of the veterans' Medicare finder file being created by the Health Care Financing Administration (HCFA) for the VA Information Resource Center (VIREC). This file will provide the HCFA identification number of study subjects and will allow us to identify participants' Medicare claims. If the HCFA-VIREC finder file is not available, we will obtained Medicare identifiers and Medicare claims data directly by providing HCFA with each subject's name, birth date, and social security number so that the agency can provide us with each subjects' HCFA identification number. We will then use this identification number to extract claims data from the Medicare Provider Analysis and Review (MEDPAR) and Medicare Part B Provider files. These files provide information about the services utilized, charges, and reimbursement. Hospital charges will be adjusted by the cost-to-charge ratio found in the hospital's Medicare cost report.

When Medicare cost data are not available, we will estimate the cost of non-VA health care using the mean cost of stays in which we have cost data. The cost of ambulatory care will be estimated by multiplying the number of units of service by the mean cost of comparable VA care. This estimate assumes that VA and non-VA hospitals have the same costs. A recent review found no evidence of difference between the cost of VA and non-VA care.[11] We will also look at data available from the HIV Cost and Services Utilization Study (HCSUS) to estimate costs for hospital health care services.[12,13]

Canadian Health Care Cost Methods

A resource utilization profile will be developed for each patient based on the minimal amount of prospective data collected from the trial. This will include hospitalization data, all drug therapy related costs, and all clinic visit and other physician visitation costs. Cost borne directly by the patient or their caregivers will also be estimated. Special attention will be given to the number of hospitalizations; reason for hospitalization; length of hospitalization; number of clinic visits; reason for visits; baseline medication; and change in medication use.

Medication Costs. Cost of study drugs, including background antiretroviral therapy, will be based on acquisition costs. Drugs costs will also include medication costs of treating all drug related adverse events. Information on prescription medications will include the name of all outpatient pharmaceuticals that the participant was receiving at the baseline visit. Changes in medication will be gathered at each subsequent study interview, including date that the participant ceased using a previously prescribed medication and the date that any new medications were begun. To find the cost of pharmaceuticals, the number of each

pharmaceutical was received will be multiplied by the average daily dose from treatment guidelines.[14]

Hospital care. The data on hospitalization/clinic visits will be combined with patient charts with similar diagnosis at St. Paul's Hospital, the largest AIDS tertiary care center in Canada. For this study, approximately 2000 HIV/AIDS admissions will be reviewed to obtain in-hospital resource use patterns for HIV/AIDS patients. Patient records will be matched according to length of stay and diagnosis. Daily per patient resource utilization will be derived and multiplied by length of stay to obtain a comprehensive utilization of services. Once a comprehensive per patient resource utilization profile is obtained, unit costs will be matched of each type of resource use to estimate total treatment costs. In-hospital unit costs will be obtained from the St. Paul's Hospital Cost Model (SPHCM). This model is a fully allocated cost model based on an economic interpretation of all hospital expenditures into overhead, support and patient care costs. All hospital overhead costs are allocated either to support-centers and patient care-centres. Support Centre costs which include admissions, admitting/discharge, record-keeping costs etc. are further "simultaneously allocated" to all patient-care centers. The model provides ward specific resource utilization per day, covering the use of hospital rooms and nursing care. Multiplied by length of stay, this provides a ward-specific length of stay cost, including personnel and overhead. Each investigational procedures, laboratory and diagnostic tests are recorded per patient. A specific number of Work Load Measurement Units (WMS) per procedure are available from Statistics Canada. Fully allocated costs per WMS are obtained from the SPHCM and combined with total WMS/procedure to obtain total costs for utilization of services. Medication use is recorded per patient across length of stay and

multiplied by unit costs to obtain total medication costs. Total costs per patient including hospitalization, medication, and cost per length of stay are aggregated to estimate total cost per patient. Since St. Paul's Hospital is a typical tertiary care teaching hospital, it is considered to be representative in cost structure to most other Canadian hospitals. The unit costs obtained from this hospital are therefore accepted as being typical Canadian in-hospital costs and have been published in a number of studies on cost-effectiveness of HIV/AIDS therapies.[15,16,17] Nonetheless, sensitivity analysis using unit costs from Calgary and Toronto hospital unit costs will also be performed.

Non Health Care System costs. These costs will be estimated from a variety of sources. Home care costs will be according to the Victorian Order of Nurses rate for home care nursing. The costs of travel will be according to cost per kilometer used in other recent studies. Statistics Canada average earning schedule will be used to calculate productivity losses for time-off work. Alternatively, the amount welfare payments received when attributable to HIV/AIDS will be used as proxy or patient reports of lost earnings (when available trial case report form). Finally, patient reports of the cost specialized equipment, special nutritional supplements, vitamins and other costs will be incorporated according to the market prices of these items.

United Kingdom Health Care Cost Methods

This section provides details of how the resource use data collected in OPTIMA will be valued in monetary terms for the U.K. analysis. Given that changes are likely to take place over the next few years in sources of cost data, the methods described here may change depending on the point of analysis undertaken. For some unit costs required for the

analysis, national statistics are available which are usually updated annually. As shown in Table B, these include fully-allocated per diem in-patient hospital costs and costs per out-patient visit, by specialty, for every UK hospital[18]; costs of specific diagnostic and therapeutic procedures for each NHS hospital[19]; undiscounted acquisition costs for all prescription medications British National Formulary[20]; and costs of community-based services[21].

However, it is recognized that some unit costs will be unavailable from these sources. For these resources, a specially-designed sub-study will be organized in the UK to estimate relevant unit costs in a sample of trial centers. For this exercise, a common costing protocol will be designed that will ensure, as far as possible, both that costs are estimated consistently in the trial centers in the sub-study and that these are consistent with costing methods used for this study in the US and Canada.

c. Measurement of Quality of Life

The goal of our cost-effectiveness analyses is to capture the effects of therapy on both length and quality of life. Because standard-ART and mega-ART may have differential effects on quality of life, and may conceivably have similar effects on length of life, careful assessment of quality of life is critical.

Central to the use of QALYs as a measure of benefit is the assignment of the quality weight for a given health state. As noted by the U.S. Panel on Cost-Effectiveness in Health and Medicine, incorporation of quality of life into such analyses requires preference-based measures of quality of life, rather than measures of functional status. Preference-based instruments are formal methods for measuring patients' health states in clinical trials and

assigning quality weights to the health states to which patients are allocated, where those weights are usually based on the preferences of a representative sample of the public.[22,23,24,25]

In addition to providing a quantified expression of how patients or the public value health outcomes, certain preference-based measures have an axiomatic basis that makes them useful in formal decision analyses and cost-effectiveness analyses.

In our primary analysis, we will estimate quality weights for QALYs using two preference-based health status measures: the HUI-III and EQ-5D. The HUI is a generic multi-attribute utility instrument.[26,27] Patients rate functional status on 6 subscales, which are then combined into a summary utility score based on an empirically derived mapping algorithm based on public preferences. Similarly, the EQ-5D is also a generic multidimensional measure of health-related quality of life.[28] It contains two parts: a health status profile and a visual analog scale to rate global health-related quality of life. Patients' health status is measured in five dimensions, defining 245 health states. A single score is available for each health state using public preferences. Using the self-rated visual analog scale scores, however, the EQ-5D can also give an indication of the respondent's personal value for that health state.

Thus, both HUI and EQ-5D are measures of quality weights for QALYs: the patient supplies information about their health state at intervals and these are 'valued' on a 0 (equivalent to death) and 1 (equivalent to good health) based on the preferences of a representative sample of the public. The advantage of HUI and EQ-5D is that they are easy and inexpensive to administer. Because the EQ-5D has limited use in Canada and U.S. and

HUI is not commonly used in the U.K., we will use both methods and report overall results using both instruments.

As a secondary analysis in a sample of US patients, we will also assess patient preferences for health states using the time-tradeoff and standard gamble which are standard approaches for global patient utilities for health states. To perform this assessment, we will use a computer-based instrument, such as U-titer, to assess patient utilities for health states. These assessments will be conducted at a sample of U.S. sites. This assessment differs from that of the HUI and EQ-5D in two important respects. First, these assessments are global measures of the utility of the health state, rather than multidimensional assessments. Second, we will assess patients' preferences with this instrument; the HUI and EQ-5D rely on preferences assessed from members of the public.

U-Titer and similar computer-based instruments have been used widely, including use in another VA cooperative trial. U-Titer has been used for utility elicitation in clinical domains such as ischemic heart disease, psoriasis, breast cancer prevention, coronary artery bypass graft surgery, low back pain, osteoporosis prevention, mild hypertension, benign prostatic hypertrophy, HIV infection, and atrial fibrillation. U-Titer program, or a similar computer-based instrument, modified for HIV and administered with a laptop computer, will be used to assess utilities of a random sample of U.S. participants. U-Titer is an interactive computer program designed to provide a standardized instrument for preference and utility assessment techniques. The use of a computerized method for utility assessment will enable the automatic generation of iterative questions that quickly enable subjects to find the appropriate tradeoff for specific health states. In addition, the program will determine an

appropriate time frame for the trade-off scenario on the basis of risk-adjusted life expectancies.

To assess quality of life, the time-tradeoff and standard gamble techniques use a series of iterative questions that ask the patient what they would be willing to trade to have a better health state. The time-tradeoff technique asks patients how much length of life they would trade to have a better quality of life. The standard gamble asks patients what risk of death they would take to have a better quality of life. For either technique, the computer-based interview asks the patients to make a series of choices about these tradeoffs. The choices presented by the computer-based interview depend on the answer to the previous question. The goal of the iterative questions is to find a choice (between better quality of life but shorter length of life) that the patient finds equally desirable. This point of indifference represents the patient's utility for the health state being assessed. For example, in a time tradeoff assessment, if a patient indicated that they were indifferent between living 10 years with their current health, and living 8 years with ideal health, the quality of life for their current health would be 8/10 or 0.8 on a scale of 0 to 1, where 0 represents death, and 1 represents ideal health. To allow patients to practice answering these questions and to understand how the computer interview works, the interview begins by asking the patients to rate the quality of life of two hypothetical health states: blindness in one or both eyes. Although the questions have been used and validated previously, we will pilot test and refine the questions if needed.

The results from these analyses will be used in the modeling part of the study, as appropriate. Changes in health-related quality of life over the time of the trial will be compared across the study arms using standard analysis approaches. A set of utility-type

weights for each patient in each time period will be calculated from the scores on the HUI or the EQ-5D instrument, and the computer-based instrument.

d. Analysis

Cost and health care utilization. We will examine whether the intervention has a significant effect on the annual health care costs incurred by participants. Average utilization patterns for each type of resource will be described for each country. Overall utilization patterns for each treatment arm in the trial will also be compared using multivariate analysis controlling for a range of covariates including country and baseline value for surrogate markers, such as CD4 count, viral load, HIV disease stage, and physiological status expressed as a Justice Score summary value.[29] Using similar methods, we will analyze total costs, including costs incurred by patients; we will also undertake separate analyses adopting the perspective of the health care payer alone.

The confidence region surrounding the cost-effectiveness ratio will be estimated using appropriate statistical methods, including bootstrap and Monte-Carlo analyses.[30] This stochastic analysis will generate cost-effectiveness acceptability curves. These curves represent a plot of the probability of one management strategy being more cost-effective than the other two at each possible critical cost-effectiveness threshold, that is, under all different assumptions about the threshold cost per QALY which defines cost-effectiveness.

Modeling. A Markov model or other state transition simulation approaches will be used for this study. The model is a mathematical simulation that evaluates the costs and benefits of a medical intervention in a hypothetical cohort of patients. General state-transition and Markov models are a standard approach for modeling diseases and

interventions that affect health or costs over long time horizons.[31,32,33] These models have been used extensively to assess screening and treatment for HIV. The model tracks a cohort of patients over time as the patients move from one health state to the next, according to transition probabilities that define the likelihood of moving from one health state to another during a given period. We will use a stochastic model, perform analyses from the societal perspective, and will discount costs and health outcomes according to current guidelines. It may be useful to perform analyses that are tailored to the costs and utilization patterns of each country, should we find that they differ.

Our decision model will compare the costs and benefits of the alternative strategies (standard-ART, mega-ART, antiretroviral drug free period, and both mega-ART and antiretroviral drug free period). Each health state incorporates natural history, treatment status, and immunologic parameters (viral load and CD4 count). Individuals start in the model with a specific treatment status and immunologic parameters. As time proceeds, individuals move among the states. Transitions occur along two dimensions; the disease state can progress (e.g., from asymptomatic infection to AIDS, with accompanying changes in immunologic parameters) and the treatment state can change (e.g., crossovers between therapies).

We model the effect of a treatment as a change in the probability of moving from one state to another. For example, the beneficial effect of treatment is modeled as a decreased transition probability from the state of asymptomatic infection to symptomatic infection (or from a lower to higher viral load). When the model is evaluated, this decreased transition probability leads to an increase in the calculated life expectancy for that person. That is, the effect of treatment can be modeled as a prolongation of the time spent before the

development of AIDS, or with AIDS, once it has developed. In summary, the model can be used to estimate the changes in length of life for a cohort of persons who are treated with alternative regimens.

To calculate quality-adjusted life years saved, we must account for changes in quality of life in the health states associated with HIV infection. To account for changes in quality of life, we apply quality adjustments to each of the relevant health states, including clinical health states and treatment regimen. The quality adjustment accounts for morbidity by "reducing" the length of life calculated for a health state that has a diminished quality of life. The reduction is accomplished by substituting the (fewer) years of healthy life that a patient finds equivalent to the length of life in the diminished health state. For example, if the quality adjustment for the asymptomatic HIV+ health state is 0.8, then a person who lives one year in that state is credited with 0.8 QALYs in the cost-effectiveness model. In this way, the calculation of QALYs enables us to express time spent in diminished health states in the equivalent amount of time spent with full health.

Each health state is also associated with costs, and the model sums the costs as the cohort transitions through various health states. The model tracks patients with alternative therapies throughout their lives, and thus calculates each of the quantities needed to calculate the incremental cost-effectiveness of the treatment.

Quality of life. Quality of life measured by HUI, EQ-5D, and the computer-based interview will be used to estimate differences, between the various management strategies evaluated in the trial, in quality-adjusted life years over the period of the trial and, through the decision model, over patients' lifetimes. Detailed analytic methods and considerations

are discussed in the section of Analysis of Secondary Outcomes of the Section G. Statistical Analysis Plan.

Recognizing the potential difference in preferences across three countries as well as the differential sensitivity of specific instruments given the circumstances of the trial, we will use three well-validated instruments. The extent to which the cost-effectiveness of the treatment strategies differs according to the alternative instruments will be assessed. We will compare quality of life measures and examine their correlation between the two instruments in all three countries. For the US patients, we will also compare the assessments performed with a computer-based interview with measures obtained from HUI and EQ-5D. If differences emerge, we will explicitly report separate results with alternative instruments, together with a discussion regarding their consistency with other outcomes (mainly clinical) collected in the study.

e. Data collection

We will collect economics data within each country to estimate both costs of each of the treatment strategies, both from a health service and a societal perspective. Due to the fact that the resource utilization data routinely available in individual countries differ, the study requires trial centres in all three countries to gather a common set of utilization and outcome data. As noted in Table B, all three countries will use the trial-case report form and baseline survey form to collect the utilization data. The common set utilization data include: hospitalizations, outpatient visits and services (including tests), prescription drug use, over-the-counter medication use, patient transportation time, patient productivity losses, and formal and informal caregiver time. The countries will also collect the costs for each of

these elements of utilization. The specific utilization and cost data to be collected within each country, and the sources of this data are summarized in Table B. Our approach aims to ensure that each country has comparable data.

As noted, quality of life is measured by three utility instruments. We will use the computer-based program to assess utilities in a randomly selected sample of 600 U.S. patients (Table C).

Table C. Utility Measuring Methods

	Indirect Measure		***Direct Measure***
	EuroQol (EQ-5D)	***Health Utilization Index HUI***	***U-Titer***
Canada	***X***	***X***	
U.K.	***X***	***X***	
U.S.	***X***	***X***	***X*** ***(Data will be collected from a random sample of 600 patients)***

X indicates the tool that will be used

f. Time frame

Economics data collection is integrated into the clinical data collection and follows the same schedule summarized in Table A.

g. Data management

Economics data will be managed in the same way as the clinic data. We adopt the same procedures and policies developed in the clinical study.

h. Quality control procedures

Economics study adopts the same strategies for quality control.

i. Organization and Administration

The economics team and study coordination committee

The economics team consists of Drs. Mark Sculpher, Kit Simpson (U.K.); Aslam Anis (Canada); Paul Barnett, Ahmed Bayoumi, Robert Nease, Douglas Owens, Gillian Sanders, and Wei Yu (U.S.).

j. Study administration

The OPTIMA economics project will be directed by a committee that will be co-chaired by three individuals, one from each participating country. The committee will meet periodically by conference call, and at least annually in person. It will review study data for accuracy and comparability between sites and countries. It will invite an outside consultant to review study methods, data, and publications. The committee will also review and approve all publications arising from study data.

k. Sub-Studies Policy

The economics study will adopts the sub-studies policy for the OPTIMA project in the Sub-Studies Policy section.

l. Publications

Publication from the economics study follows the same policy for the OPTIMA project.

References

1. Stinnett AA, Mullahy J. Net health benefits: a new framework for the analysis of uncertainty in cost-effectiveness analysis [see comments]. *Med Decis Making* 1998; 18:S68-80.

2. Briggs AH. A Bayesian approach to stochastic cost-effectiveness analysis. *Health Econ* 1999; 8:257-61.

3. Van Hout BA, Al MJ, Gordon GS, Rutten FF. Costs, effects and C/E-ratios alongside a clinical trial. *Health Econ* 1994; 3:309-19.

4. Drummond MF, Stoddart GL, Torrance GW. *Methods for the economic evaluation of health care programmes*. Oxford: Oxford Medical Publications, 1987.

5. Gold MR, Siegel JE, Russell LB, Weinstein MC. *Cost-effectiveness in health and medicine*. New York: Oxford University Press, 1996.

6. Willke RJ, Glick HA, Polsky D, Schulman K. Estimating country-specific cost-effectiveness from multinational clinical trials. *Health Economics* 1998;7: 481-493

7. Luce B, Manning W, Siegel J, Lipscomb J. Estimating costs in cost-effectiveness analysis. In: Gold, MR, Siegel, JE, Russell, LB, Weinstein, MC Eds. Cost-effectiveness in health and medicine. New York: Oxford University Press, 1996.

8. Swindle R, VanDeusen-Lukas C, Alexander-Meyer D, et al. Cost analysis in the Department of Veterans Affairs: Consensus and future directions. *Medical Care (in press)* 1999; 37:AS3-AS8.

9. Barnett PG, Rodgers JH. Use of the decision support system for VA cost-effectiveness research. *Medical Care* 1999; 37:AS63-AS70.

10. Barnett PG. Research without billing data. Econometric estimation of patient-specific costs. *Med Care* 1997; 35:553-63.

11. Hendricks AM, Remler DK, Prashker MJ. More or Less? Comparisons of VA and non-VA health care costs. *Medical Care* 1999; 37:AS54-AS62.

12. Joyce GF, Goldman DP, Leibowitz A, et al. Variation in inpatient resource use in the treatment of HIV: do the privately insured receive more care? [see comments]. *Med Care* 1999; 37:220-7.

13. Shapiro MF, Morton SC, McCaffrey DF, et al. Variations in the care of HIV-infected adults in the United States: results from the HIV Cost and Services Utilization Study [see comments]. *JAMA* 1999; 281:2305-15

14. BC Centre for Excellence in HIV/AIDS. Therapeutic Guidelines for the Treatment of HIV/AIDS and related conditions. Vancouver, BC.

15. Anis AH, Hogg RS, Yip B, et al. Average annual drug cost and its determinants in a population based cohort of HIV-positive adult men and women. *Pharmacoeconomics* 1998; 13:327-36.

16. Anis AH, Hogg RS, Wang XH, et al. Modeling the potential economic impact of viral load-driven triple drug combination antiretroviral therapy. *Pharmacoeconomics* 1998; 13:697-705.

17. Anis AH, Guh D, Hogg RS, et al. The cost effectiveness of antiretroviral regimes for the treatment of HIV/AIDS. *Pharmacoeconomics* 2000, in press.

18. The Chartered Institute of Public Finance and Accountancy (CIPFA), 1999.

19. NHS Executive, 1999.

20. British National Formulary, 2000.

21. Netten A, Dennett J, Knight J. *Unit Costs of Health and Social Care*. Canterbury: PSSRU, University of Kent, 1998

22. Weinstein MC, Fineberg HV. *Clinical decision analysis*. Philadelphia: W.B.Saunders Company, 1980.

23. Sox H, Blatt M, Higgins M, Marton K. *Medical Decision Making*. Boston: Butterworths, 1988.

24. Owens DK, Sox HC. Medical Decision Making: Probabilistic Medical Reasoning. In: Shortliffe, EH, Perreault, LE, Fagan, LM, Wiederhold, G Eds. Medical Informatics: Computer Applications in Health Care. Reading: Addison-Wesley, 1990:70-116.

25. Howard RA. Risk Preference. In: Howard, RA, Matheson, JE Eds. The Principles and Applications of Decision Analysis. Menlo Park: Strategic Decisions Group,1984:627-663.

26. Torrance GW, Furlong W, Feeny D, Boyle M. Multi-attribute preference functions. Health Utilities Index. *Pharmacoeconomics* 1995; 7:503-20.

27. Feeny D, Furlong W, Boyle M, Torrance GW. Multi-attribute health status classification systems. Health Utilities Index. *Pharmacoeconomics* 1995; 7:490-502.

28. Kind P. The EuroQoL instrument: an index of health-related quality of life. In: Spilker, B Ed. Quality of Life and Pharmacoeconomics in Clinical Trials. Philadelphia: Lippincott-Raven, 1996.

30. Justice AC, Feinstein AR, Wells CK. A new prognostic staging system for the acquired immunodeficiency syndrome [see comments]. *N Engl J Med* 1989; 320:1388-93.

31. O'Brien BJ, Drummond MF, Labelle RJ, Willan A. In search of power and significance: issues in the design and analysis of stochastic cost-effectiveness studies in health care. *Med Care* 1994; 32:150-63.

32. Beck JR, Pauker SG. The Markov process in medical prognosis. *Med Decis Making* 1983; 3:419-458.

33. Sonnenberg FA, Beck JR. Markov models in medical decision making: a practical guide. *Med Decis Making* 1993; 13:322-38.

34. Naimark D, Krahn MD, Naglie G, et al. Primer on medical decision analysis: Part 5--Working with Markov processes [see comments]. *Med Decis Making* 1997; 17:152-9

APPENDICES

1. Sample Informed Consent

FULL TITLE

A Tri-National (Canada, UK, USA) Randomized Controlled Trial To Determine The Optimal Management Of Patients With HIV Infection For Whom First And Second-Line Highly Active Antiretroviral Therapy Has Failed.

TNT-1: OPTIMA

INTRODUCTION

You are being invited to take part in a research study at the VA(*Facility Name*). Before you decide to participate, it is important for you to understand why the research is being done and what it will involve. Please take time to read the following information carefully and discuss it with friends, relatives and your doctor if you wish. Ask us if there is anything that is not clear or if you would like more information. Take time to decide whether or not you wish to participate.

PURPOSE

The purpose of this study is to determine the best way to treat HIV infection after treatment with several highly active combinations of HIV drugs has failed to have an effect on your HIV infection. The study is looking for people whose anti-HIV treatment has stopped working and who have a rising level of HIV in their blood. The study asks two questions. Firstly, does a period of time taking no anti-HIV drugs help the next treatment you take work better than not stopping anti-HIV drugs for a drug-free period? Secondly, will a new (salvage) combination treatment with many drugs lead to a longer improvement in health than a combination containing fewer drugs? The study will also address the safety of these two different strategies.

You are being asked to participate in this study because you are HIV positive, you have taken at least two initially effective combinations of anti-HIV medications, and your current treatment for HIV is no longer working (suppressing your virus). Also, your CD4 (T-Helper) cell count is less than 300 so that you are at risk for opportunistic infections and other HIV related conditions. Approximately 500 persons in the US, Canada, and the United Kingdom will be in the study.

DESCRIPTION OF THE STUDY

This study compares the use of 5 or more anti-HIV drugs (Mega-Antiretroviral Therapy or Mega-ART) to 4 or fewer drugs (Standard-Antiretroviral Therapy or Standard-ART). The study also compares stopping HIV treatment for three months to not stopping treatment. The current standard of care uses 3 drugs from 2 classes of medications. The medicines used within the study are all either licensed drugs or are those available through Expanded Access Programs. No new agents are being tested in this study although the way they are being used is experimental. It is a trial to define strategy for treatment and to determine which is the best approach to treatment after anti-retroviral treatment failure. You may enroll in studies of other investigational drugs and remain on this study as long as you continue to follow the rules outlined in this form.

PROCEDURES

If you decide you want to be in this study you will be asked to come into the clinic for a visit to determine if you are eligible to participate. At this visit you will be asked to read and sign this consent form. After signing, medical tests will be completed to help determine if you meet the requirements to be in the study. These are called “Screening” tests.

The screening tests you will be required to complete will include:
- HIV antibody test (if this has not been done at your hospital).
- Blood test to determine HIV RNA blood levels
- Blood test to measure your immune system (CD4 and CD8)
- Pregnancy test (for women only)
- Blood draw for possible viral drug resistance testing at a later date
- Blood draw for safety tests

Any medications you are currently taking will be recorded and you will be asked if you have taken your currently prescribed medications as directed.

For the screening, the amount of blood drawn will be 4 tablespoons (60 mL). All results of the blood tests will remain private. If you meet the study criteria and choose to participate, you will need to return for study enrollment (Baseline/Day 1).

The following will tests/procedures will be performed at the Baseline visit:
- The same laboratory tests drawn at screening
- Medical history evaluation, including a review of medications taken to treat HIV
- A physical examination
- Questionnaires about how you feel and about your current level of health.

You will then be assigned randomly, like flipping a coin, to one of four treatment groups. The treatment groups are as follows:

- Anti-retroviral drug free period of 3 months followed by Standard-ART

  or
- Anti-retroviral drug free period of 3 months followed by Mega-ART

  or
- Immediate start of Standard-ART (4 anti-retroviral drugs or less)

  or
- Immediate start of Mega-ART (5 or more anti-retroviral drugs)

There is an equal chance that you will be randomized to any one of the four groups.

This is an open study meaning that, once you are on treatment, both you and the study personnel will know what group you are assigned to. But, if you are randomized to have an anti-retroviral drug free period, neither you nor the study personnel will be told whether you are going to later be taking Mega-ART or Standard-ART until after the treatment interruption is completed.

Before you begin treatment in the study, your doctor will be given results of the resistance test taken at screening to help guide the selection of a treatment regimen. When it is time for you to begin treatment, your doctor will recommend what drugs, in either a Mega-ART or Standard-ART regimen, are most likely to benefit you.

Blood will be taken at each visit during the study to conduct safety tests, to see if the treatment option is working by measuring the amount of HIV in the blood (viral load), and to check your immune system (CD4 cells). Blood will also be drawn and stored for future tests such as changes in resistance of the virus to the drugs. An average of 4 tablespoons (60mL) of blood will be drawn at each study visit. You will be given a brief physical examination at each visit if you have noticed any symptoms or side effects. Women will also be given regular pregnancy tests as the drugs could harm an unborn child.

At each visit, you will be asked about any medications you might have taken and any illness that might have occurred since the last visit. Any changes in your medication and/or your health will be recorded and you will be asked direct questions about how you feel and be asked to complete a "quality of life" questionnaire.

You will be followed on the study between 1 year and 4 years, depending upon when you enrolled. If you are randomly assigned to an anti-retroviral drug free period, you will be seen 6 weeks after starting the anti-retroviral drug-free period for blood tests and a symptom directed examination. If your CD4 cell count has fallen by 50% or more since the start of the study, you may decide with your doctor to begin treatment before completing the 3 month anti-retroviral drug free period. You will be seen 2 and 6 weeks after starting Mega- or Standard-ART, then every 3 months afterwards. This should be similar to what you would normally expect in ordinary treatment. Your doctor may wish to see you more frequently for other reasons.

Each regular follow-up visit will take about 1 hour. The first and final visits will take about 1½ hours.

While you are participating in this study you should not use any medications (over-the-counter, prescription, or street drugs) without telling your study doctor. This is important as these drugs may react badly with the drugs you are taking.

DISCOMFORTS AND RISKS

For everyone entering this study there is no course of treatment that we know will halt or slow progression of HIV disease. You may therefore experience complications that result from the progression of HIV during the course of this study. However, your doctor and the study team will make every effort to identify, prevent or treat any complications that may arise.

During the trial your doctor will be following your progress very closely and will explain your treatment options to you if it becomes necessary to consider a change of therapy. There may be specific risks associated with each of the treatment arms to which you may be assigned. These are described below.

RISKS OF CHANGING FROM A FAILING REGIMEN

There may be risks associated with changing from your current regimen even though it is failing. Your current medical regimen may be providing some protection against more rapid progression of your HIV disease. Your study physician is recommending a change in your anti-HIV treatment in the hope that further progression can be slowed or halted, but it is not known if this will occur. All anti-HIV medications have been associated with side-effects and toxicities to varying degrees. Your doctor can review the details of these possible reactions when your treatment regimen is decided upon, but will also answer questions about these medications before you sign this form, if you wish. In addition, there is a risk that you will develop further resistance to the drugs in this study.

RISKS OF AN ANTI-VIRAL DRUG FREE PERIOD

An aim of this study is to determine whether a 3 month interruption in anti-HIV treatment will lead to an improved sense of well-being and to a better long-term response. We don't know the answer to these questions. The amount of HIV in your blood may increase after medications (including those that are failing) are withdrawn. The CD4 count may decrease rapidly when medications (including those that are failing) are withdrawn. Consequently, having a break in treatment may mean that your disease will progress more rapidly. You will have an extra monitoring visit 6 weeks after stopping treatment in order to minimize this risk if you are assigned to this arm.

RISKS OF MEGA-ART

There is no clear information regarding the tolerability of these complex regimens, the potential for toxicities, or their long-term effectiveness. In small studies, up to 40% of persons taking a Mega-ART regimen may achieve an undetectable HIV viral load on this treatment, at least for a while. However, it is anticipated that some persons may experience too many side effects to remain on the drugs. Your doctor can review the details of these possible reactions when you decide on your treatment regimen, and now, if you wish.

RISKS OF STANDARD-ART

The risks associated with a change from one Standard-ART regimen to another are similar to those that may occur with any change in regimen. It is not anticipated that a change in Standard-ART will lead to full suppression of the HIV virus that you are infected with. Your doctor will use the results of HIV resistance testing to select a regimen that should be more effective than the regimen you were on before the study. It may help slow the progression of HIV but this is another question that we don't have the answer to. The virus may develop resistance to drugs included in the regimen that you have not previously taken. You may experience drug-related side effects or toxicities from the medicines in the new regimen. Your doctor can review the details of these possible reactions when your treatment regimen is decided upon, and now, if you wish.

RISKS AND DISCOMFORTS OF STUDY PROCEDURES

Using a needle and syringe to remove blood from the vein is called "a blood draw". It may be necessary to insert a needle into your vein more than once if blood does not come out the first time. At each scheduled blood draw, a new needle will be used. During blood draws you may have pain and/or bruising at the place on your arm where blood is taken. Blood clots can form and infections may occur, but these are very rare. If you feel faint during or after a blood draw, you should lie down right away to avoid falling down, then you should notify one of the study staff.

Your doctor will be following your progress very closely and will explain your treatment options to you if it becomes necessary.

RISKS DURING PREGNANCY

It is possible that some of the treatments used in HIV disease when given to pregnant women may harm the unborn child. For female subjects, it is therefore very important to not become pregnant during this study. If you are a woman who is able to become pregnant, and choose to have sex during this study, you must agree to use an acceptable method of birth control throughout the study. Your doctor can tell you which brands of contraceptives are acceptable for you to use. Acceptable birth control methods include:

- Condom and spermicide
- Diaphragm and spermicide

Even if you use an acceptable birth control method, you could still become pregnant. You are aware that not having sex is the only certain way to prevent pregnancy. There is a slight chance that a pregnancy test could be wrong. If the pregnancy test is wrong, and you receive the study medication while pregnant, the study drug may harm an unborn baby.

ALTERNATIVE THERAPY

If you do not wish to participate in this study, the alternative therapy could potentially include all or any possible combinations of medications that are used in this study. You can receive this alternative therapy outside the study at your VA facility. Further, if you choose not to participate in this study, you will continue to receive the medical care which your doctor feels is most appropriate and your decision will not affect the level of medical care that you are eligible to receive.

WITHDRAWAL FROM THE STUDY

Your participation in this study is entirely voluntary. You may withdraw from this study at any time without penalty or loss of benefits and you will be treated with the best available options by your doctor. In addition, your participation in the study could be ended if continuation would not be safe for you or if one of the review committees overseeing the study recommends discontinuation of the study or your medical center's participation. In the event that this occurs, you will continue to receive the standard medical care at your medical facility.

BENEFITS

You may not personally benefit from taking part in this study. You may benefit if the medications prescribed successfully prevent progression of your underlying HIV infection but this may not occur. Your participation may lead to knowledge that will help others. All anti-retroviral medications used in this study will be provided free of charge to the study participants.

COSTS AND PAYMENTS

There will be no costs to you as a result of the study. In the event that an injury occurs resulting from your participation in this study, medical treatment will be available at your VA Medical Center at no cost to you.

RESEARCH RESULTS

1. The answers to the study questions that you provide will be kept in a locked office at *(name of Medical Center)* and at the VA Cooperative Studies Coordinating Office in West Haven, CT. Confidentiality of this study's records identifying you will be maintained within these offices.

2. If results of this study are reported in medical journals or at meetings, you will not be identified by name, by recognizable photograph, or by any other means without your specific consent. Your medical records will be maintained according to this medical center's requirements. However, there is a possibility that the Veterans' Affairs Headquarters for Research and Development or its designees may inspect the records relating to this study.

BLOOD SAMPLES

Some of the blood that is being drawn will be frozen for future tests of viral resistance and other studies. This blood will be stored without any personal identifiers at a centralized laboratory. You understand that by signing this consent form you are authorizing the use of this blood for these future analyses and studies.

SPECIAL INFORMATION

1. You are not required to take part in this study: your participation is entirely voluntary
2. Your decision to participate will not affect your benefits or eligibility for medical care
3. As a veteran-subject you will not be required to pay for any treatment received as a research subject that is being done solely for the purpose of this research study. However, your insurance carrier will be billed for all routine care and clinical procedures outside the protocol, if applicable. If you are in the 'discretionary work load' category, you are subject to making a copayment as indicated by a means test (defined in M-1,pt.1,ch.4, par.4.30). Your doctor should provide you with this information or refer you to the appropriate individual for any questions you may have. In accordance with Federal Law, as a veteran, you will receive medical care and treatment for injuries suffered as a result of participating in a VA research program. Financial compensation for such things as lost wages or disability due to research-related injury is not available.
4. You can refuse to participate now or withdraw from the study at any time after giving your consent. This will not interfere with the medical treatment you would be receiving if you were not enrolled in this study.
5. New findings developed during the course of the study and which relate to your willingness to continue participation will be provided to you
6. If you have questions about your rights as a study participant, you may contact the patient representative (*Name of contact and Local phone number*)
7. A copy of this consent form will be placed in your medical record and sent to the Veterans' Affairs Cooperative Studies Program Coordinating Center (VACSPCC), West Haven, CT
8. The VA Medical facility shall provide necessary medical treatment to you as a research subject injured as a result of participation in a research project approved by a VA Research and Development Committee and conducted under the supervision of one or more VA employees in accordance with Federal Regulations.
9. At the end of the study, the doctor treating you will continue to provide care and use the appropriate medications in your regimen. The costs of your medical care after the study is over will depend on the level of coverage to which you are entitled under VA regulations.
10. At the conclusion of the trial, you will be sent a letter thanking you for participation and describing the study findings.

RESEARCH SUBJECTS' RIGHTS: I have read or have had read to me all of the above. __________ has explained the study to me and answered all of my questions. I have been told of the risks or discomforts and possible benefits of the study. I have been told of other choices of treatment available to me.

I understand that I do not have to take part in this study, and my refusal to participate will involve no penalty or loss of rights to which I am entitled. I may withdraw from this study at any time without penalty or loss of VA or other benefits to which I am entitled.

The results of this study may be published, but my records will not be revealed unless required by law.

In case there are medical problems or questions, I have been told I can call Dr.___________ at__________during the day and Dr. ________at ____________ after hours. If any medical problems occur in connection with this study the VA will provide emergency care.

I understand that should I wish to discuss my participation in this study with any other doctor or layperson, I can contact.___NAME_____ ACOS-R&D Program by requesting an appointment at **[Phone #]** hospital extension #____, first floor in the research building, room #_____. Questions concerning conduct of a study or of subject research rights, should also be addressed to **[Not Investigator/or Staff]**. Medical problems during the course of the study should be addressed to the investigator at the phone listed above.

I understand my rights as a research subject, and I voluntarily consent to participate in this study. I understand what the study is about and how and why it is being done. I will receive a signed copy of this consent form.

____

Subject's Signature Date

____

Signature of Subject's Representative* Date Subject's Representative (print)

____

Signature of Witness Date Witness (print)

____

Signature of person obtaining consent** Date

____

Signature of Investigator Date

*Only required if subject not competent.
** Only required if not investigator

2. Presumptive and Definitive Criteria for AIDS events in OPTIMA

Based on the 1993 US Centers for Disease Control and Prevention definitions (MMWR 1992; 41 [No RR-17]: 1-19) and modified for this trial.

	PRESUMPTIVE CRITERIA	DEFINITIVE CRITERIA
**CONSTITUTIONAL DISEASE**		
HIV Wasting syndrome	Unexplained, involuntary weight loss >10% from baseline (week 0) PLUS persistent diarrhea with 2 or more liquid stools/day > 1 month OR chronic weakness OR persistent fever > 1 month. Should exclude other causes such as cancer, TB, MAI, cryptosporidiosis or other specific enteritis	None
**INFECTIONS**		
Aspergillosis, invasive pulmonary	A plus B: (A) CXR abnormality compatible with aspergillosis, (B) invasive mycelia consistent with Aspergillus on lung biopsy or positive culture of lung tissue or positive culture of sputum collected by any method	A plus B plus C: (A) CXR abnormality compatible with aspergillosis, (B) invasive mycelia consistent with Aspergillus on lung biopsy; (C) positive culture of lung tissue or positive culture of sputum collected by any method
Aspergillosis, other invasive	A plus B: (A) clinical evidence of invasive infection, (B) invasive mycelia consistent with Aspergillus on tissue biopsy or positive culture at a normally sterile site (e.g. blood) apart from the involved tissue	A plus B plus C: (A) compatible clinical course, (B) invasive mycelia consistent with Aspergillus on tissue biopsy or clinical evidence of infection, (C) positive culture from the affected tissue
Bartonellosis	A plus B: (A) Clinical evidence of bacillary angiomatosis or bacillary peliosis, (B) positive silver stain for bacilli from a skin lesion or an affected organ	A plus B: (A) Clinical or histologic evidence of bacillary angiomatosis or bacillary peliosis, (B) a positive culture or PCR for *B. quintana* or *B. henselae*
Candidiasis of bronchi, trachea or lungs	None	macroscopic appearance at bronchoscopy or autopsy, or histology or cytology/smear (not culture)
Esophageal candidiasis	Recent onset of retrosternal pain on swallowing PLUS a clinical diagnosis of oral candidiasis or by cytology smear (not culture), PLUS clinical response to treatment	macroscopic appearance at endoscopy or autopsy, or histology or cytology/smear (not culture)
Coccidiodomycosis, disseminated or extrapulmonary	None	histology or cytology, culture or antigen detection from affected tissue
Cryptococcosis, meningitis or extrapulmonary	None	histology or cytology/microscopy, culture or antigen detection from affected tissue
Cryptosporidiosis	None	persistent diarrhea > 1 month, histology or microscopy
CMV retinitis	Symptomatic or asymptomatic. Typical appearance on fundoscopy of discrete patches of retinal whitening, spreading along blood vessels, associated with vasculitis, hemorrhage and necrosis, confirmed by ophthalmologist.	none
CMV end-organ disease	None	compatible symptoms, plus histology or detection of antigen from affected tissue

	PRESUMPTIVE CRITERIA	DEFINITIVE CRITERIA
CMV radiculomyelitis	Leg weakness and decreased reflexes or syndrome consistent with cord lesion presenting subacutely over days to weeks. Myelogram shows no mass lesion. CSF shows >5 WBC with >50% polymorphs and no other pathogen or persistence of symptoms after appropriate treatment for other pathogens, OR CMV shown by PCR, antigen or culture	none
CMV meningoencephalitis	Rapid (days to 1-4 weeks) syndrome with progressive delirium, cognitive impairment +/- seizures and fever (often with other CMV disease elsewhere). CT/MRI may show periventricular abnormalities with or without contrast enhancement. CSF may be normal or show evidence of CMV	none
HSV mucocutaneous ulceration	None	persistent ulceration documented > 1 month, plus histology or culture or detection of antigen from affected tissue
HSV visceral disease, e.g. bronchitis, pneumonitis, oesophagitis	None	symptoms, plus histology or culture or detection of antigen from affected tissue
HZV, multidermatomal	A plus B: (A) ≥10 typical ulcerated lesions affecting at least 2 non-contiguous dermatomes, (B) response to an antiviral active against HZV unless resistance is demonstrated	A plus B: (A) ≥10 typical ulcerated lesions affecting at least 2 non-contiguous dermatomes, (B) culture or detection of antigen from affected tissue
Histoplasmosis, disseminated or extrapulmonary	None	symptoms, plus histology or culture or detection of antigen from affected tissues
Isosporiasis	None	persistent diarrhea > 1 month, histology or microscopy
Leishmaniasis, visceral	None	symptoms, plus histology
Microsporidiosis	None	persistent diarrhea > 1 month, histology or microscopy
MAC, and other atypical mycobacteriosis	Symptoms of fever, fatigue, anemia or diarrhea, plus AFBs seen in stool, blood, body fluid or tissue but not grown on culture, and no concurrent diagnosis of TB, except pulmonary	symptoms of fever, fatigue, anemia or diarrhea, culture from stool, blood, body fluid or tissue, except pulmonary
Tuberculosis, pulmonary	Symptoms of fever, dyspnoea, cough, weight loss or fatigue, plus AFBs seen in sputum or lavage or lung tissue but not grown in culture, plus responds to standard TB treatment	symptoms of fever, dyspnoea, cough, weight loss or fatigue, plus culture from sputum or lavage or lung tissue
Tuberculosis, extrapulmonary	Symptoms, plus AFBs seen from affected tissue or blood but not grown in culture, concurrent diagnosis of pulmonary TB or responds to standard TB treatment	symptoms, plus culture from blood or affected tissue
Nocardiosis	Clinical evidence of invasive infection plus microscopic evidence of bronchial weakly acid fast organisms from the affected tissue	Clinical evidence of invasive infection plus a positive culture from the affected tissue or blood
Penicillium marneffei, disseminated	Known presence in a *P. marnefffei* endemic area plus characteristic skin lesions plus response to antifungal therapy for penicilliosis	Culture from a non-pulmonary site
PCP	Recent symptoms, plus typical CXR appearance if on PCP prophylaxis or any CXR appearance if not on prophylaxis and CD4 <200, negative bronchoscopy if already treated for PCP for > 7 days or	microscopy or histology

	not done, no bacterial pathogens in sputum, and responds to PCP treatment	
Extrapulmonary *pneumocystis*	None	symptoms plus microscopy or histology
Recurrent bacterial pneumonia	Second pneumonic episode within 1 year, new CXR appearance, symptoms and signs, diagnosed by a doctor	second pneumonic episode with 1 year, new CXR appearance, detection of bacterial pathogen
	**PRESUMPTIVE CRITERIA**	**DEFINITIVE CRITERIA**
PML, Progressive multifocal leukoencephalopathy	Symptoms and brain scan consistent with PML, and no response to toxo treatment	histology
Rhodococcus equi disease	None	Clinical evidence of invasive infection plus microbiologic identification of the organism in the affected tissue or blood
Recurrent salmonella septicaemia	None	second distinct episode, culture
Cerebral toxoplasmosis	Symptoms of focal intracranial abnormality or decreased consciousness, and brain scan consistent with lesion(s) having mass effect or enhanced by contrast, and either positive toxoplasma serology or responds to treatment clinically and by scan	histology or microscopy
Other extrapulmonary toxoplasmosis	None	symptoms plus histology or microscopy
**NEOPLASMS**		
KS, Kaposi's sarcoma	Typical appearance without resolution. Clinicians who have seen few cases should not make presumptive diagnoses	Histology
Primary cerebral lymphoma	Symptoms consistent with lymphoma, at least one lesion with mass effect on brain scan, no response clinically and by scan to toxoplasma treatment	Histology
Lymphoma, B-cell, non-Hodgkin's l	none	histology
Lymphoma, Hodgkin's	None	Histology
Cervical carcinoma, invasive	none	histology, not carcinoma-in-situ
**NEUROLOGICAL**		
HIV encephalopathy	Cognitive or motor dysfunction interfering with usual activity, progressive over weeks or months in the absence of another condition to explain the findings, should have brain scan +/- CSF to exclude other causes. should be grade 2 or worse in at least 2 domains by NARS (see below) excluding abnormal domains at trial entry	none
**OTHER**		
Indeterminate intracerebral lesion(s)	Neurological illness with evidence for an intracerebral lesion(s) by brain scan where the differential diagnosis is either cerebral toxoplasmosis, PML, cerebral lymphoma or HIV encephalopathy	none

NAR	Cognitive-					

S Stages	Behavioral Domains					
	**Orientation**	**Memory**	**Motor**	**Behavior**	**Problem Solving**	**Activities of daily living**
**0.5**	**Fully oriented**	**Complains of memory problems**	**Fully ambulatory slightly slowed movements**	**Normal**	**Has slight mental slowing**	**Slight impairment in business dealings**
**1**	**Fully oriented, may have brief periods of "spaciness"**	**Mild memory problems**	**Balance, coordination and handwriting difficulties**	**More irritable, labile or apathetic, withdrawn**	**Difficulty planning and completing work**	**Can do simple tasks, may need daily prompting**
**2**	**Some disorientation**	**Memory moderately impaired, new learning impaired**	**Ambulatory but may require walking aid**	**Some impulsivity or agitated behavior**	**Severe impairment, poor social judgment, gets lost easily**	**Needs assistance with ADLs**
**3**	**Frequent disorientation**	**Severe memory loss, only fragments of memory remain**	**Ambulatory with assistance**	**May have organic psychosis**	**Judgment very poor**	**Cannot live independently**
**4**	**Confused and disoriented**	**Virtually no memory**	**Bedridden**	**Mute and unresponsive**	**No problem solving ability**	**Nearly vegetative**

3. NIAID Standardized Toxicity Grading System

Item	Grade 1 Toxicity	Grade 2 Toxicity	Grade 3 Toxicity	Grade 4 Toxicity
Hematology				
Hemoglobin (g/dL)	9.5 - 10.5	8.0 – 9.4	6.5 – 7.9	< 6.5
Absolute Neutrophil Count (x 10^9/L	1.0 – 1.5	0.750 – 0.999	0.500 – 0.749	<0.500
Platelets (x 10^9/L)	75 – 99	50 – 74.9	20 – 49.9	<20 or diffuse petechiae
Enzymes				
AST (SGOT)	1.25 – 2.5 x upper normal limit	2.6 x 5 x upper normal limit	5.1 – 10 x upper normal limit	> 10 x upper normal limit
Alkaline phosphatase	1.25 – 2.5 x upper normal limit	2.6 – 5 x upper normal limit	5.1 – 10 x upper normal limit	> 10 x upper normal limit
Amylase	1.1 – 1.5 x upper normal limit	1.6 – 2.0 x upper normal limit	2.1 – 5.0 x upper normal limit	> 10 x upper normal limit
Chemistries				
Hyponatremia (mmol/L)	130 – 135	123 – 129	116 – 122	< 116 or mental status changes or seizures
Hypernatremia (mmol/L)	146-150	151-157	158-165	> 165 or mental status changes or seizures
Hypokalemia (mmol/L)	3.0 – 3.4	2.5 – 2.9 or replacement Rx required	2.0 – 2.4 or intensive replacement Rx req. or hospitalization req.	< 2.0 or paresis or ileus or life-threatening arrhythmia
Hyperkalemia (mmol/L)	5.6 – 6.0	6.1 – 6.5	6.6 – 7.0	> 7.0 or life threatening arrhythmias
Hypoglycemia (mmol/L)	3.0 – 3.5	2.2 – 2.9	1.7 – 2.1	< 1.7 or mental status changes or coma
Hyperglycemia (mmol/L) (note if fasting)	6.4 – 8.9	9.0 – 13.9	14.0 - 28.0	> 28 or ketoacidosis
Hyperbilirubinemia (µmol/L)	24 – 33	34 – 55	56 – 110	> 110
Urea	1.25 – 2.5 x upper normal limit	2.6 – 5 x upper normal limit	5.1 – 10 x upper normal limit	> 10 x upper normal limit
Creatinine (µmol/L)	130 – 180	181 – 360	361 – 720	> 720 or dialysis required

Urinalysis				
Proteinuria	1+ or <0.3% or < 3 g/L or 200 mg – 1 g loss/day	2 – 3+ or 0.3 – 1.0% or 3 – 10 g/L or 1 – 2 g loss/day	4+ or >1.0% or > 10 g/L or 2 – 3.5 g loss/day	> 3.5 g loss/day or nephrotic syndrome
Hematuria	microscopic only	Gross, no clots	gross + clots	Requires transfusion or causes obstruction
Cardiac Dysfunction				
Cardiac Rhythm		Asymptomatic, transient signs, no Rx required	recurrent/persistent; no Rx required	requires treatment
Hypertension	transient inc < 20 mm; no Rx	Recurrent, chronic, > 20 mm, Rx required	requires acute therapy (outpatient)	requires hospitalization
Hypotension	transient orthostatic hypotension; no Rx	Symptoms correctable with oral fluid Rx	requires IV fluids; no hospitalization required	requires hospitalization
Pericarditis	minimal effusion	Mild/mod asymp. Effusion; no Rx	symptomatic effusion, pain, EKG changes	tamponade; pericardiocentesis or surgery required
Hemorrhage, blood loss	microscopic; occult	Mild, no transfusion	gross blood loss; 1 to 2 units transfused	massive blood loss, or ≥ 3 units transfused
Respiratory				
Cough – for aerosol studies	transient – no Rx	Treatment-associated cough; local Rx	uncontrolled	
Bronchospasm acute	transient; no Rx < 80% - > 70% FEV_1 (or peak flow)	Req Rx; normalizes with bronchodilator; or FEV_1 50% to 70% (or peak flow)	no normalization w/bronchodilator or FEV_1 25% - 50% (or peak flow); retraction	cyanosis or FEV_1 < 25 % (or peak flow) or intubated
Neuro/Neuromuscular				
Neuro-cerebellar	slight incoordination dysdiadochokinesis	Intention tremor, dysmetria, slurred speech; nystagmus	locomotor ataxia	incapacitated
Mood	mild anxiety or mild depression	Therapy required and moderate anxiety or moderate depression	needs assistance for severe anxiety or severe depression or severe mania	acute psychosis or incapacitated or hospitalization

Neuro control (ADL = Activities of Daily Living)	no Rx, ADL unaffected, mild difficulty concentrating or mild confusion or mild agitation	Some limitation of ADL requiring minimal therapy or moderate confusion or moderate agitation	therapy required, assistance for ADL and severe confusion or agitation	toxic psychosis or incapacitated or hospitalization
Muscle strength	subjective weakness; no objective	Mild objective signs, symptoms	objective weakness; function limited	paralysis
Gastrointestinal				
Stomatitis	Mild discomfort, no limits on activity	Some limits on eating/drinking	eating/talking very limited	requires IV fluids
Nausea	mild discomfort, maintains reasonable intake	Moderate discomfort; intake significantly decreased; some activity limited	severe discomfort; no significant intake; activities limited	minimal fluid intake
Vomiting	transient emesis	Occ/moderate vomiting	orthostatic hypotension or IV fluid required	hypotensive shock or hospitalization req for IV fluid
Constipation	mild	Moderate	severe	distension with vomiting
Diarrhea	transient or 3 –4 loose stools/day	5 – 7 loose stools/day or nocturnal loose stools	orthostatic hypotension or > 7 loose stools/day or IV fluid Rx req.	hypotensive shock or hospitalization req for IV fluids
Other Parameters				
Fever; oral, > 12hrs	37.7 – 38.5 C	38.6 – 39.5 C	39.6 – 40.5 C	> 40.5 C
Headache	mild, no rx	Transient, moderate Rx	severe, responds to initial narcotic therapy	intractable, req. repeated narcotic therapy
Fatigue	no decrease in activity	Normal activity decreased 25-50%	normal activity decreased > 50%; can't work	unable to care for self.
Allergic Reaction	pruritus w/o rash	Localized urticaria	generalized urticaria or angioedema	anaphylaxis
Local Reaction	tenderness or erythema	Induration < 10 cm or phlebitis or inflammation	induration > 10 cm or ulceration	necrosis

Mucocutaneous	erythema, pruritis	Diffuse, maculopapular rash, dry desquamation	vesiculation, moist desquamation, ulceration	exfoliative dermatitis, mucous membrane involvement, or erythema multiforme or suspected Stevens-Johnson or necrosis requiring surgery

4. ICH Harmonized Tripartite Guidelines for Serious Adverse Events Reporting

INTERNATIONAL CONFERENCE ON HARMONISATION OF TECHNICAL REQUIREMENTS FOR REGISTRATION OF PHARMACEUTICALS FOR HUMAN USE

ICH HARMONISED TRIPARTITE GUIDELINE CLINICAL SAFETY DATA MANAGEMENT: DEFINITIONS AND STANDARDS FOR EXPEDITED REPORTING

Recommended for Adoption at Step 4 of the ICH Process on 27 October 1994 by the ICH Steering Committee.

This Guideline has been developed by the appropriate ICH Expert Working Group and has been subject to consultation by the regulatory parties, in accordance with the ICH Process. At Step 4 of the Process the final draft is recommended for adoption to the regulatory bodies of the European Union, Japan and USA.

CLINICAL SAFETY DATA MANAGEMENT:
DEFINITIONS AND STANDARDS FOR EXPEDITED REPORTING

ICH Harmonised Tripartite Guideline

Having reached Step 4 of the ICH Process at the ICH Steering Committee meeting on 27 October 1994, this guideline is recommended for adoption to the three regulatory parties to ICH

I. INTRODUCTION

It is important to harmonise the way to gather and, if necessary, to take action on important clinical safety information arising during clinical development. Thus, agreed definitions and terminology, as well as procedures, will ensure uniform Good Clinical Practice standards in this area. The initiatives already undertaken for marketed medicines through the CIOMS-1 and CIOMS-2 Working Groups on expedited (alert) reports and periodic safety update reporting, respectively, are important precedents and models. However, there are special circumstances involving medicinal products under development, especially in the early stages and before any marketing experience is available. Conversely, it must be recognised that a medicinal product will be under various stages of development and/or marketing in different countries, and safety data from marketing experience will ordinarily be of interest to regulators in countries where the medicinal product is still under investigational-only (Phase 1, 2, or 3) status. For this reason, it is both practical and well-advised to regard pre-marketing and post-marketing clinical safety reporting concepts and practices as interdependent, while recognising that responsibility for clinical safety within regulatory bodies and companies may reside with different departments, depending on the status of the product (investigational vs. marketed).

There are two issues within the broad subject of clinical safety data management that are appropriate for harmonisation at this time:

(1) the development of standard definitions and terminology for key aspects of clinical safety reporting, and

(2) the appropriate mechanism for handling expedited (rapid) reporting, in the investigational (i.e., pre-approval) phase.

The provisions of this guideline should be used in conjunction with other ICH Good Clinical Practice guidelines.

II. DEFINITIONS AND TERMINOLOGY ASSOCIATED WITH CLINICAL SAFETY EXPERIENCE

A. Basic Terms

Definitions for the terms adverse event (or experience), adverse reaction, and unexpected adverse reaction have previously been agreed to by consensus of the more than 30 Collaborating Centres of the WHO International Drug Monitoring Centre (Uppsala, Sweden). [Edwards, I.R., et al, Harmonisation in Pharmacovigilance. *Drug Safety* 10(2): 93-102, 1994.] Although those definitions can pertain to situations involving clinical investigations, some minor modifications are necessary, especially to accommodate the pre-approval, development environment. The following definitions, with input from the WHO Collaborative Centre, have been agreed:

1. Adverse Event (or Adverse Experience)

Any untoward medical occurrence in a patient or clinical investigation subject administered a pharmaceutical product and which does not necessarily have to have a causal relationship with this treatment.

An adverse event (AE) can therefore be any unfavourable and unintended sign (including an abnormal laboratory finding, for example), symptom, or disease temporally associated with the use of a medicinal product, whether or not considered related to the medicinal product.

2. Adverse Drug Reaction (ADR)

In the *pre-approval clinical experience* with a new medicinal product or its new usages, particularly as the therapeutic dose(s) may not be established:

all noxious and unintended responses to a medicinal product related to any dose should be considered adverse drug reactions.

The phrase "responses to a medicinal products" means that a causal relationship between a medicinal product and an adverse event is at least a reasonable possibility, i.e., the relationship cannot be ruled out.

Regarding *marketed medicinal product*s, a well-accepted definition of an adverse drug reaction in the post-marketing setting is found in WHO Technical Report 498 [1972] and reads as follows:

A response to a drug which is noxious and unintended and which occurs at doses normally used in man for prophylaxis, diagnosis, or therapy of disease or for modification of physiological function.

The old term "side effect" has been used in various ways in the past, usually to describe negative (unfavourable) effects, but also positive (favourable) effects. It is recommended that this term no longer be used and particularly should not be regarded as synonymous with adverse event or adverse reaction.

3. Unexpected Adverse Drug Reaction

An adverse reaction, the nature or severity of which is not consistent with the applicable product information (e.g., Investigator's Brochure for an unapproved investigational medicinal product). (See section III.C.)

B. Serious Adverse Event or Adverse Drug Reaction

During clinical investigations, adverse events may occur which, if suspected to be medicinal product-related (adverse drug reactions), might be significant enough to lead to important changes in the way the medicinal product is developed (e.g., change in dose, population, needed monitoring, consent forms). This is particularly true for reactions which, in their most severe forms, threaten life or function. Such reactions should be reported promptly to regulators.

Therefore, special medical or administrative criteria are needed to define reactions that, either due to their nature ("serious") or due to the significant, unexpected information they provide, justify expedited reporting.

To ensure no confusion or misunderstanding of the difference between the terms "serious" and "severe," which are not synonymous, the following note of clarification is provided:

The term "severe" is often used to describe the intensity (severity) of a specific event (as in mild, moderate, or severe myocardial infarction); the event itself, however, may be of relatively minor medical significance (such as severe headache). This is not the same as "serious," which is based on patient/event outcome or action criteria usually associated with events that pose a threat to a patient's life or functioning. Seriousness (not severity) serves as a guide for defining regulatory reporting obligations.

After reviewing the various regulatory and other definitions in use or under discussion elsewhere, the following definition is believed to encompass the spirit and meaning of them all:

A serious adverse event (experience) or reaction is any untoward medical occurrence that at any dose:

** results in death,*

** is life-threatening,*

NOTE: The term "life-threatening" in the definition of "serious" refers to an event in which the patient was at risk of death at the time of the event; it does not refer to an event which hypothetically might have caused death if it were more severe.

** requires inpatient hospitalisation or prolongation of existing hospitalisation,*

** results in persistent or significant disability/incapacity, or*

** is a congenital anomaly/birth defect.*

Medical and scientific judgment should be exercised in deciding whether expedited reporting is appropriate in other situations, such as important medical events that may not be immediately life-threatening or result in death or hospitalisation but may jeopardise the patient or may require intervention to prevent one of the other outcomes listed in the definition above. *These should also usually be considered serious.*

Examples of such events are intensive treatment in an emergency room or at home for allergic bronchospasm; blood dyscrasias or convulsions that do not result in hospitalisation; or development of drug dependency or drug abuse.

C. Expectedness of an Adverse Drug Reaction

The purpose of expedited reporting is to make regulators, investigators, and other appropriate people aware of new, important information on serious reactions. Therefore, such reporting will generally involve events previously unobserved or undocumented, and a guideline is needed on how to define an event as "unexpected" or "expected" (expected/unexpected from the perspective of previously observed, not on the basis of what might be anticipated from the
pharmacological properties of a medicinal product). As stated in the definition (II.A.3.), an "unexpected" adverse reaction is one, the nature or severity of which is not consistent with information in the relevant source document(s). Until source documents are amended,

expedited reporting is required for additional occurrences of the reaction. The following documents or circumstances will be used to determine whether an adverse event/reaction is expected:
1. For a medicinal product not yet approved for marketing in a country, a company's Investigator's Brochure will serve as the source document in that country. (See section III.F. and ICH Guideline for the Investigator's Brochure.)
2. Reports which add significant information on specificity or severity of a known, already documented serious ADR constitute unexpected events. For example, an event more specific or more severe than described in the Investigator's Brochure would be considered "unexpected". Specific examples would be (a) acute renal failure as a labeled ADR with a subsequent new report of interstitial nephritis and (b) hepatitis with a first report of fulminant hepatitis.

III. STANDARDS FOR EXPEDITED REPORTING
A. What Should be Reported?

1. Single Cases of Serious, Unexpected ADRs
All adverse drug reactions (ADRs) that are both serious and unexpected are subject to expedited reporting. This applies to reports from spontaneous sources and from any type of clinical or epidemiological investigation, independent of design or purpose. It also applies to cases not reported directly to a sponsor or manufacturer (for example, those found in regulatory authority-generated ADR registries or in publications). The source of a report (investigation, spontaneous, other) should always be specified.
Expedited reporting of reactions which are serious but expected will ordinarily be inappropriate. Expedited reporting is also inappropriate for serious events from clinical investigations that are considered not related to study product, whether the event is expected or not. Similarly, non-serious adverse reactions, whether expected or not, will ordinarily not be subject to *expedited* reporting.
Information obtained by a sponsor or manufacturer on serious, unexpected reports from any source should be submitted on an expedited basis to appropriate regulatory authorities if the minimum criteria for expedited reporting can be met. See section III.B.
Causality assessment is required for clinical investigation cases. All cases judged by either the reporting health care professional or the sponsor as having a reasonable suspected causal relationship to the medicinal product qualify as ADRs. For purposes of reporting, adverse event reports associated with marketed drugs (spontaneous reports) usually imply causality. Many terms and scales are in use to describe the degree of causality (attributability) between a medicinal product and an event, such as certainly, definitely, probably, possibly or likely related or not related. Phrases such as "plausible relationship," "suspected causality," or "causal relationship cannot be ruled out" are also invoked to describe cause and effect. However, there is currently no standard international nomenclature. The expression "reasonable causal relationship" is meant to convey in general that there are facts (evidence) or arguments to suggest a causal relationship.

2. Other Observations
There are situations in addition to single case reports of "serious" adverse events or reactions that may necessitate rapid communication to regulatory authorities; appropriate medical and scientific judgment should be applied for each situation. In general, information that might materially influence the benefit-risk assessment of a medicinal product or that would be

sufficient to consider changes in medicinal product administration or in the overall conduct of a clinical
investigation represents such situations. Examples include:
a. For an "expected," serious ADR, an increase in the rate of occurrence which is judged to be clinically important.
b. A significant hazard to the patient population, such as lack of efficacy with a medicinal product used in treating life-threatening disease.
c. A major safety finding from a newly completed animal study (such as carcinogenicity).

B. Reporting Time Frames

1. Fatal or Life-Threatening Unexpected ADRs
Certain ADRs may be sufficiently alarming so as to require very rapid notification to regulators in countries where the medicinal product or indication, formulation, or population for the medicinal product are still not approved for marketing, because such reports may lead to consideration of suspension of, or other limitations to, a clinical investigations program. Fatal or life-threatening, unexpected ADRs occurring in *clinical investigations* qualify for very rapid
reporting. Regulatory agencies should be notified (e.g., by telephone, facsimile transmission, or in writing) as soon as possible but no later than 7 calendar days after first knowledge by the sponsor that a case qualifies, followed by as complete a report as possible within 8 additional calendar days. This report must include an assessment of the importance and implication of the findings, including relevant previous experience with the same or similar medicinal products.

2. All Other Serious, Unexpected ADRs
Serious, unexpected reactions (ADRs) that are not fatal or life-threatening must be filed as soon as possible but no later than 15 calendar days after first knowledge by the sponsor that the case meets the minimum criteria for expedited reporting.

3. Minimum criteria for reporting
Information for final description and evaluation of a case report may not be available within the required time frames for reporting outlined above. Nevertheless, for regulatory purposes, initial reports should be submitted within the prescribed time as long as the following minimum criteria are met: an identifiable patient; a suspect medicinal product; an identifiable reporting source; and an event or outcome that can be identified as serious and unexpected, and for which, in clinical investigation cases, there is a reasonable suspected causal relationship. Follow-up information should be actively sought and submitted as it becomes available.

C. How to Report
The CIOMS-I form has been a widely accepted standard for expedited adverse event reporting. However, no matter what the form or format used, it is important that certain basic information/data elements, when available, be included with any expedited report, whether in a tabular or narrative presentation. The listing in Attachment 1 addresses those data elements regarded as desirable; if all are not available at the time of expedited reporting, efforts should be made to obtain them. (See section III.B.) All reports must be

sent to those regulators or other official parties requiring them (as appropriate for the local situation) in countries where the drug is under development.

D. Managing Blinded Therapy Cases

When the sponsor and investigator are blinded to individual patient treatment (as in a double-blind study), the occurrence of a serious event requires a decision on whether to open (break) the code for the specific patient. If the investigator breaks the blind, then it is assumed the sponsor will also know the assigned treatment for that patient. Although it is advantageous to retain the blind for all patients prior to final study analysis, when a serious adverse reaction is judged reportable on an expedited basis, it is recommended that the blind be broken only for that specific patient by the sponsor even if the investigator has not broken the blind. It is also recommended that, when possible and appropriate, the blind be maintained for those persons, such as biometrics personnel, responsible for analysis and interpretation of results at the study's conclusion.

There are several disadvantages to maintaining the blind under the circumstances described which outweigh the advantages. By retaining the blind, placebo and comparator (usually a marketed product) cases are filed unnecessarily. When the blind is eventually opened, which may be many weeks or months after reporting to regulators, it must be ensured that company and regulatory data bases are revised. If the event is serious, new, and possibly related to the medicinal product, then if the Investigator's Brochure is updated, notifying relevant parties of the new information in a blinded fashion is inappropriate and possibly misleading. Moreover, breaking the blind for a single patient usually has little or no significant implications for the conduct of the clinical investigation or on the analysis of the final clinical investigation data. However, when a fatal or other "serious" outcome is the primary efficacy endpoint in a clinical investigation, the integrity of the clinical investigation may be compromised if the blind is broken. Under these and similar circumstances, it may be appropriate to reach agreement with regulatory authorities in advance concerning serious events that would be treated as disease-related and not subject to routine expedited reporting.

E. Miscellaneous Issues

1. Reactions Associated with Active Comparator or Placebo Treatment

It is the sponsor's responsibility to decide whether active comparator drug reactions should be reported to the other manufacturer and/or directly to appropriate regulatory agencies. Sponsors must report such events to either the manufacturer of the active control or to appropriate regulatory agencies. Events associated with placebo will usually not satisfy the criteria for an ADR and, therefore, for expedited reporting.

2. Products with More than one Presentation or Use

To avoid ambiguities and uncertainties, an ADR that qualifies for expedited reporting with one presentation of a product (e.g., a dosage form, formulation, delivery system) or product use (e.g., for an indication or population), should be reported or referenced to regulatory filings across other product presentations and uses. It is not uncommon that more than one dosage form, formulation, or delivery system (oral, IM, IV, topical, etc.) of the pharmacologically active compound(s) is under study or marketed; for these different presentations there may be some marked differences in the clinical safety profile. The same may apply for a given product used in different indications or populations (single dose vs. chronic administration, for example). Thus, "expectedness" may be product or product-use

specific, and separate Investigator's Brochures may be used accordingly. However, such documents are expected to cover ADR information that applies to all affected product presentations and uses. When relevant, separate discussions of pertinent product-specific or use-specific safety information will also be included. It is recommended that any adverse drug reactions that qualify for expedited reporting observed with one product dosage form or use be cross referenced to regulatory records for all other dosage forms and uses for that product. This may result in a certain amount of overreporting or unnecessary reporting in obvious situations (for example, a report of phlebitis on IV injection sent to authorities in a country where only an oral dosage form is studied or marketed). However, underreporting is completely avoided.

3. Post-study Events

Although such information is not routinely sought or collected by the sponsor, serious adverse events that occurred after the patient had completed a clinical study (including any protocol-required post-treatment follow-up) will possibly be reported by an investigator to the sponsor. Such cases should be regarded for expedited reporting purposes as though they were study reports. Therefore, a causality assessment and determination of expectedness are needed for a
decision on whether or not expedited reporting is required.

F. INFORMING INVESTIGATORS AND ETHICS COMMITTEES/INSTITUTIONAL REVIEW BOARDS OF NEW SAFETY INFORMATION

International standards regarding such communication are discussed within the ICH GCP Guidelines, including the addendum on "Guideline for the Investigator's Brochure." In general, the sponsor of a study should amend the Investigator's Brochure as needed, and in accord with any local regulatory requirements, so as to keep the description of safety information updated.

Attachment 1

KEY DATA ELEMENTS FOR INCLUSION IN EXPEDITED REPORTS OF SERIOUS ADVERSE DRUG REACTIONS

The following list of items has its foundation in several established precedents, including those of CIOMS-I, the WHO International Drug Monitoring Centre, and various regulatory authority forms and guidelines. Some items may not be relevant depending on the circumstances. The minimum information required for expedited reporting purposes is: an identifiable patient, the name of a suspect medicinal product, an identifiable reporting source, and an event or outcome that can be identified as serious and unexpected and for which, in clinical investigation cases, there is a reasonable suspected causal relationship. Attempts should be made to obtain follow-up information on as many other listed items pertinent to the case.

1. Patient Details

Initials
Other relevant identifier (clinical investigation number, for example)
Gender

Age and/or date of birth
Weight
Height

2. Suspected Medicinal Product(s)
Brand name as reported
International Non-Proprietary Name (INN)
Batch number
Indication(s) for which suspect medicinal product was prescribed or tested
Dosage form and strength
Daily dose and regimen (specify units - e.g., mg, ml, mg/kg)
Route of administration
Starting date and time of day
Stopping date and time, or duration of treatment

3. Other Treatment(s)
For concomitant medicinal products (including non-prescription/OTC medicinal products) and non-medicinal product therapies, provide the same information as for the suspected product.

4. Details of Suspected Adverse Drug Reaction(s)
Full description of reaction(s) including body site and severity, as well as the criterion (or criteria) for regarding the report as serious should be given. In addition to a description of the reported signs and symptoms, whenever possible, attempts should be made to establish a specific diagnosis for the reaction.
Start date (and time) of onset of reaction
Stop date (and time) or duration of reaction
Dechallenge and rechallenge information
Setting (e.g., hospital, out-patient clinic, home, nursing home)

Outcome: information on recovery and any sequelae; what specific tests and/or treatment may have been required and their results; for a fatal outcome, cause of death and a comment on its possible relationship to the suspected reaction should be provided. Any autopsy or other post-mortem findings (including a coroner's report) should also be provided when available. Other information: anything relevant to facilitate assessment of the case, such as medical history including allergy, drug or alcohol abuse; family history; findings from special investigations.

5. Details on Reporter of Event (Suspected ADR)
Name
Address
Telephone number
Profession (specialty)

6. Administrative and Sponsor/Company Details
Source of report: was it spontaneous, from a clinical investigation (provide details), from the literature (provide copy), other?
Date event report was first received by sponsor/manufacturer
Country in which event occurred

Type of report filed to authorities: initial or follow-up (first, second, etc.)
Name and address of sponsor/manufacturer/company
Name, address, telephone number, and FAX number of contact person in reporting company or institution
Identifying regulatory code or number for marketing authorisation dossier or clinical investigation process for the suspected product (for example IND or CTX number, NDA number)
Sponsor/manufacturer's identification number for the case (this number must be the same for the initial and follow-up reports on the same case).

7. SAMPLE SIZE/POWER CALCULATIONS/LEVEL OF SIGNIFICANCE

Disease progression rate in the Standard-ART arm

The rate of progression to a new AIDS defining event or death in the ritonavir arm of the Cameron study was 29/100 person-years corresponding to a cumulative event rate of 25% by one year.[1] Patients entering OPTIMA on standard therapy are expected to have much higher rates since none of the drugs will be of a new class that might lead to more durable remission from disease progression.

Data from the EuroSIDA cohort (A. Phillips, personal communication, June 2000) yield, in patients exposed to PI and NNRTI for more than 6 months and with plasma Viral Load (pVL) > 10000 copies/ml, event rates of 77 events in 201 person-years (0.38 per year) and 17 in 173 person-years (0.1 per year) corresponding to cumulative event rates of 32% and 10% by one year for CD4 count < 100 and between 100 and 200 cells/ml respectively. The cumulative event rates by one year were 43% and 23% for CD4 count < 50 and CD4 count < 200 respectively.

In October 2002, after an evaluation of recently released results from two randomized studies (GIGHAART and the MDR-HIV study)[a,b] and a review of the accumulated data in OPTIMA, it was concluded that the original sample size estimate was based on too conservative assumptions about the primary event (progression to a new AIDS defining event or death), treatment crossover and loss to follow-up rates. Sample size estimates are largely driven by the assumed primary event rate (the smaller the event rate, the larger is the required sample size), the expected treatment difference in the primary event rate, the treatment crossover rate and (to a lesser extent) the loss to follow-up rate.

Crossover between the two arms during follow-up

The rate of crossover from mega-ART to standard-ART (expected to be largely due to toxicity) is likely to be higher initially and to decrease during follow-up. In the Multi-Drug Rescue Therapy cohort, the proportion of patients reducing from 5 or more drugs to 4 or less was 23% by one year.[2] The proportion reducing to three or less would be smaller. Data from other cohorts showed a lower rate of crossover. Although 25% of 106 patients on mega-ART with a median follow-up of 15 months experienced severe laboratory abnormalities, only 6 (5%) discontinued treatment.[3]

The crossover rate from Mega-ART to Standard-ART in OPTIMA was originally assumed to be 20% in Year 1 with a 50% reduction every year thereafter. In the GIGHAART study, only 8% receiving giga-HAART reduced the number of drugs in their regimen to < 6 drugs. The observed crossover rate in OPTIMA from mega-ART to standard ART is less than 1% in Year 1. It is anticipated that such crossovers will occur early in the follow-up since the primary reason for reducing the number of drugs would be toxicity. The drop-in rate from Standard-ART to Mega-ART was initially assumed to be 5% in the first year, but no drop-ins in the first year have been observed in the study.

Loss to follow-up

The three Clinical Trial organizations have an established record of following the vast majority of patients in clinical trials. Linkages with national AIDS & death registries will assist by providing mechanisms for ancillary follow-up.

The loss to follow-up was initially assumed to be 10% by the end of the study. This

follow-up refers to inability to assess primary endpoints (death and/or AIDS events). All three co-coordinating centers have been involved in multi-center trials for a number of years

and the track record of tracking such endpoints has been excellent. Tracking is aided by the existence of databases where study endpoints can be detected and verified, meaning that loss to follow-up for events is expected to be smaller than originally assumed (10%). To maintain the conservative nature of our assumptions, a 5% loss to follow-up was incorporated into the revised sample size estimation.

All the sample size and power calculations have been done according to the methods proposed by Lakatos, allowing for staggered entry and assumes an exponential distribution of the event rate.[4,5,6] In calculating sample size, the following assumptions were made:

- Standard-ART Event rate at Year 1 is 23%; with a 25% increase annually thereafter until the end of the study
- Hazard reduction of 30% with full adherence (unadjusted for losses and crossovers)
- Loss to follow-up at 5.5 years will be 5%
- Drop-in (Standard to Mega) is 1% Year 1; increases 10% every year thereafter
- Drop-out (Mega to Standard) is 5% Year 1; decreases 50% every year thereafter
- Alpha-level 0.05
- 4.5 year accrual; 1 year minimum follow-up

The table below provides a summary of the original and the revised assumptions used in sample size estimation.

ORIGINAL ASSUMPTIONS (v1.1)	REVISED ASSUMPTIONS
- Standard-ART cumulative event rate at Year 1 is **13%** with a 25% annual increase thereafter until the end of the study<br>- Crossover (Mega to Standard) is **20%** in Year 1; decreases 50% every year thereafter<br>- Hazard reduction of 30% with full adherence (unadjusted for losses and crossovers; adjusted hazard reduction 22.7%)<br>- Loss to follow-up at **3.5** years is **10%**<br>- Drop-in (Standard to Mega) is **5%** Year 1; increases 10% every year thereafter<br>- Alpha-level of 0.05<br>- **2.5** year accrual; 1 year minimum follow-up<br>- Power of 80%<br><br>Sample size: **1700**<br>Number of events: **450**	- Standard-ART cumulative event rate at Year 1 is **23%** with a 25% annual increase thereafter until the end of the study<br>- Crossover (Mega to Standard) is **5%** in Year 1; decreases 50% every year thereafter<br>- Hazard reduction of 30% with full adherence (unadjusted for losses and crossovers; adjusted hazard reductions are shown in table 3)<br>- Loss to follow-up at **5.5** years is **5%**<br>- Drop-in (Standard to Mega) is **1%** in Year 1; increases 10% every year thereafter<br>- Alpha-level of 0.05<br>- **4.5** year accrual; 1 year minimum follow-up<br>- Power of 80%<br><br>Sample size: **503**<br>Number of events: **292**

Table 3 provides re-estimated sample sizes assuming a 24-month extension of accrual and a range of event rates (15%-28%). The table also gives the number of patients required to be enrolled each month from November 1, 2002 to the stated end of accrual to achieve the revised sample size (taking into account the 142 patients who are randomized as of November 1, 2002).

With the above assumptions and using a log rank test with intention-to-treat, a trial of 503 patients (with 292 expected events) will be sufficient to detect a difference between treatment strategy groups (power 0.80; type I error 0.05).

Table 3

Standard 1-yr	Power	Events	Standard	Mega Event	AHR	N	Accrual	Follow-	Rands/

Rate			Event Rate %	Rate %	%		to	up to	month
15%	80%	297	48.4	38.9	25.5	680	12/31/05	12/31/06	14
18%	80%	295	54.6	44.6	25.2	595	12/31/05	12/31/06	12
21%	80%	293	60.0	49.8	24.8	535	12/31/05	12/31/06	10
**23%**	**80%**	**292**	**63.2**	**52.9**	**24.7**	**503**	**12/31/05**	**12/31/06**	**9**
25%	80%	291	66.1	55.9	24.3	477	12/31/05	12/31/06	9
28%	80%	290	70.0	60.0	23.9	446	12/31/05	12/31/06	8

AHR = Adjusted Hazard Reduction

References:

1. Cameron DW et al. Randomized placebo-controlled trial of ritonavir in advanced HIV-1 disease. Lancet 1998; 351:543-549

2. Montaner J et al. Multi-drug rescue therapy (MDRT) in three cohorts of HIV-positive individuals. 3rd International Workshop on Salvage Therapy for HIV Infection, Chicago, IL, April 2000. Abstract 24. Antiviral Therapy 2000; 5 (suppl 2);21

3. Staszweski S et al. Virological and Immunological response to mega-HAART salvage therapy after failure of multiple antiretroviral regimens. Abstracts of the 4th International Congress on Drug Therapy for HIV Infection: May 19-21, 1999; Toronto, Ontario. Abstract p.94

4. Lakatos E. Sample Size Determination in Clinical Trials with time-dependent rates of losses and non-compliance. *Controlled Clin Trials* 1986;**7**:189-199

5. Lakatos E. Sample Size based on the log-rank statistic in complex clinical trials. *Biometrics* 1988; **44**:229-241.

6. Shih JH. Sample Size Calculation for Complex Clinical Trials with Survival Endpoints. Controlled Clin Trials 1995;**16**:395-407

8. Statistical Analysis Plan

Interim Monitoring and Analysis

Interim monitoring will focus on patient intake--overall and within country, adherence to the protocol, adverse events, baseline comparability of treatment groups, and completeness of data retrieval. Prototype sets of tables and figures for trial monitoring by (a) TMC and TSC, and (b) DSMB are provided in **Appendix 9**.

(a) TMC and TSC Monitoring

The number of patients enrolled (overall and by country) will be tabulated. If possible, the combined as well as country-specific proportion of enrolled/eligible patients will also be examined. Patients enrolled in OPTIMA by month and hospital within each country, along with the number of minor and major protocol violations will be tabulated. The number of patients randomized in each CD4 stratum will also be provided (IN Tables 1-4, Appendix 9)

Baseline characteristics will be tabulated: demographic information, medical history, HIV-stage antiretroviral therapy, opportunistic infections medication, risk factors, quality of life, virological markers, hematology, biochemistry, and physical examination information. This will be a descriptive analysis with no formal test or p-values for differences between any comparison groups. Antiretroviral therapy history, current anti-HIV medications, and baseline virological and immunological markers, will be tabulated by country (Base Tables 5-8, Appendix 9).

At follow-up visits data will be collected to examine the following variables: the composition of the first ART regimen on-study and the follow-up ART regimens, virological and immunological markers, as well as process results. Tables and plots

describing these variables will be generated for the trial overall and by country (Fables 9-11, Appendix 9).

Compliance to the allocated strategy will be provided in C Figures 3a-3c, Appendix 9.

Grade 3 and 4 Adverse Events and Serious Adverse Events will be tabulated by country and overall (AE Tables 12-13, Appendix 9).

Outcomes will be provided by country and overall in OUT Table 14, Appendix 9.

(b) DSMB Monitoring

At every DSMB meeting, the number of patients enrolled (overall, by country and by treatment strategy) will be tabulated. The number of minor and major protocol violations will be tabulated by country as well as the number of patients randomized in each CD4 stratum (DSMB Monitoring Tables, Section A, Tables 1-4, Appendix 9).

Baseline characteristics will be tabulated by treatment strategy: demographic information, medical history, HIV-stage antiretroviral therapy, opportunistic infections medication, risk factors, quality of life, virological markers, hematology, biochemistry, and physical examination information. This will be a descriptive analysis with no formal test or p-values for differences between any comparison groups. Antiretroviral therapy history, current anti-HIV medications, and baseline virological and immunological markers, will be tabulated by country (DSMB Monitoring Tables, Section A, Tables 4-7, Appendix 9).

At follow-up visits data will be collected to examine the following variables: the composition of the first ART regimen on-study and the follow-up ART regimens for each treatment strategy (DSMB Monitoring Tables, Section A, After Randomization/Follow-up, Tables 1a-1d, and Tables 4a-4d, Appendix 9).

Grade 3 and 4 Adverse Events and Serious Adverse Events will be tabulated by country and overall (DSMB Monitoring Tables, Section A, After Randomization/Follow-up, Tables 2a-3, Appendix 9).

Compliance will be assessed for each of the four treatment strategies (DSMB Monitoring Tables, Section A, After Randomization/Follow-up, Figures 1a-1e, Appendix 9)

Two full interim analyses will be undertaken when 110 and 225 primary events have accumulated. For the primary endpoint analysis the Haybittle-Peto method for monitoring will be used with a nominal p-value of 0.001 for each interim look and p-value of 0.05 for the final analysis.[1]

Outcomes will be provided by treatment strategy at the interim analysis (DSMB Monitoring Tables, Section B, Interim Analysis, Tables 1, Appendix 9). Changes in virological and immunological markers by treatment strategy will also be provided at the interim analysis. (DSMB Monitoring Tables, Section B, Interim Analysis, Figures 1-4 and Life- Tables and Figures, Appendix 9)

ii. Final Analysis

Evaluation of Participant Accounting and Eligibility Violations

Participant Disposition

The number of patients enrolled (overall, by country and by month, and by screening CD4 count stratum) will be tabulated and presented (Tables B1 and B4 /Figures B1). Study subjects who withdrew prior to study termination will be discussed in the clinical study report.

Eligibility Violations

Tables B2 and B3 will list all major and minor known eligibility violations.

The tables will provide a descriptive summary of each such violation. Major violations are defined as (a) errors in the process of randomization and (b) violations of inclusion/exclusion criteria, which were not reviewed and allowed/exempted.

Evaluation of Baseline Characteristics

In order to assess the adequacy of randomization, baseline characteristics will be tabulated by allocation (overall and by country) in terms of median, interquartile range, mean and standard deviation where appropriate, and distribution tabulations. Baseline characteristics include: demographic information (age, gender, race), HIV-stage, antiretroviral therapy, opportunistic infections medication, likely mode of infection, virological and immunological markers. This will be a descriptive analysis with no formal test or p-values for differences between the allocation groups. (Tables B5, B6, B7 and B8).

The classes of medications as well as the number of antiretrovirals that patients are discharged with at study entry will be described for the whole study population (Table F2).

Description of follow-up

The total follow-up time will be calculated in person-years and the number of study participants whose survival status is not known for more than 6 months will be described by allocation group and summarized by treatment strategy. (Table F1)

Compliance to Treatment strategy

The proportion of subjects on assigned treatment strategy will

be displayed over time (Figures F1(a)-(d)). Changes in treatment strategy will be recorded, as well as the reasons for these changes.

Evaluation of Primary and Secondary Endpoints

The analysis of the primary endpoint [time to (new or recurrent) AIDS-defining event or death] will be done according to intent-to-treat, that is according to original treatment assignment, regardless of adherence. (Tables O1-O3; Figures O1-O5)

The primary comparisons will be the main effects: Standard-ART vs Mega-ART and Anti-retroviral Drug-free Period (ARDFP) vs No Drug-Free Period (No ARDFP). **These comparisons will be made using the stratified log-rank test for time to event analysis.[1] There will be a total of 12 strata derived according to country, screening CD4 count and other allocated treatment strategy in patients randomized according to the original 2X2 design.** All p-values will be two-tailed and the Type I error of 0.05 will be used as the level of significance.

A test of interaction between the main effects will be undertaken using data from subjects who were enrolled using the 2X2 randomization scheme. Statistically significant qualitative interactions between the main effects (treatment strategies) are not anticipated (some moderate quantitative interactions are possible and will be assessed in a Cox analysis). In the event such interactions are statistically significant, the analysis of the main effects will be performed according to allocation status. For example, the comparison between Mega vs Standard-ART will be performed separately according to ARDFP status.

Standard failure time methods (Kaplan-Meier plots, log rank test and Cox regression techniques) will be employed to make comparisons of efficacy (primary

endpoints: death and AIDS-defining events) and toxicity (secondary endpoints: non HIV-related serious adverse events; Tables O5 and Figures O6) between the treatment strategy groups.[1] The Kaplan-Meier (KM) estimate will be used to display the estimated probability of freedom from events across time. Standard errors of this estimate will be computed using the modified Greenwood formula and will be used to construct confidence intervals around the KM estimates.

If the primary analyses of main effects are significant, secondary analyses will examine the pair wise treatment comparisons. In order to provide some control for multiplicity for these secondary analyses, the Hochberg procedure, a sequentially rejective variation of the Bonferroni procedure, will be used to determine the significance at a Type I error of 0.05 (two-sided).[2]

Additional analysis adjusting for baseline covariates will be undertaken using Cox analysis. These covariates are: CD4 count, viral load, previous history of antiretroviral treatment.

Evaluation of Other Outcomes

Other outcomes include: (a) at 12 months, absolute CD4 T-cell counts adjusted for baseline value and changes in log viral load and (b) incidence of grade 3 or 4 clinical or laboratory adverse event (Tables O4). These will be compared between the four allocated arms in the 2X2 randomization scheme.

The other important outcome is quality of life as measured by the MOS-HIV, HUI, EuroQOL (and U-titer in the VA). The rationale for an ARDFP is improved quality of life (QOL) with minimal impact on survival. Analyses comparing Standard-ART vs Mega-ART will examine the entire follow-up period; for

comparisons of ARDFP vs No ARDFP, analyses will also examine the initial 3 months when these differential strategies are to be applied. A more detailed analysis plan for health economics and quality of life will be prepared separately.

Health Economic Analysis

Resources such as hospital care, medication, outpatient visits, and long-term care will be collected for all patients in each strategy arm and compared using t-tests or Wilcoxon rank sum tests. These outcomes will be compared over subgroups using analysis of variance techniques or the Kruskall-Wallis test. The p-values in subgroups will be adjusted using approaches for multiple hypothesis testing. (for details see Health Economics Component, Section XVI)

Safety

The incidence of any adverse events will be calculated and compared among the treatment strategies using chi-square or Wilcoxon-type statistics, as appropriate.

Other analyses

Further analyses will include:

- Changes in CD4 cell counts over the follow-up period
- Changes in plasma HIV RNA (log10 HIV RNA) at follow-up visits and average change from nadir over the duration of follow-up will be assessed both graphically and by applying longitudinal methods where appropriate[16]
- Comparison of Viral Resistance at 12 months among treatment strategy groups (for patients with viral loads dictated by drug-resistance assays detection limits)
- Comparison of use of antiretrovirals by treatment strategy arm

REFERENCES

1. Kalbfleish JD and Prentice PL. The Statistical Analysis of Failure Time Data. John Wiley & Sons, New York, NY: 1980

2. Hochberg Y. A sharper Bonferroni procedure for multiple tests of significance. Biometrika 1988; **2**:93-113.

References:

1. Haybittle JL. Significance Testing in the Comparison of Survival Curves from Clinical Trials of Cancer Treatment. Eur J Cancer Clin Oncol 1986; 22:1279-1283

2. Tukey JW. Exploratory Data Analysis. Addison-Wesley, Reading MA: 1977

3. Chambers JW, Clevland WS, Kleiner B, Tukey PA. Graphical Methods for Data Analysis. Duxbury Press, New York, NY: 1983

4. Silvey SD. Statistical Inference. Halsted Press, John Wiley & Sons, New York, NY: 1975

5. Kalbfleish JD and Prentice PL. The Statistical Analysis of Failure Time Data. John Wiley & Sons, New York, NY: 1980

6. Cox DR and Oakes D. Analysis of Survival Data. Chapman and Hall, New York, NY: 1984

7. Hample FR, Rousseeuw PJ, Ronchetti EM, Stahel WA. Robust Statistics. John Wiley, New York, NY:1986

8. Hochberg Y. A sharper Bonferroni procedure for multiple tests of significance. Biometrika 1988; **2**:93-113.

9. Laird NM, and Ware JH. Random-effects models for longitudinal data. Biometrics 1982; 35:795-802.

10. Rubin D. Multiple Imputation after 18+ years. J Am Stat Assoc 1996; 91:473-489

11. Lavori PW, Dawson R and Shera. A multiple imputation strategy for clinical trials with truncation of patient data. Stat Med 1995; 14:1913-1925.

12. Curran D, Molenberghs G, Fayers PM, Machin D. Incomplete quality of life data in randomized trials: missing forms. Stat Med 1998; 17: 697-709.

13. Senn S, Stevens L, Chaturvedi N. Repeated measures in clinical trials: simple strategies for analysis using summary measures. Stat Med 2000; 19: 861-879.

14. McHorney CA, Ware JE, Raczek AE. The MOS 36-item Short-Form Health Survey (SF-36): II. Psychometric and clinical tests of validity in measuring physical and mental health construct. Med Care 1993; 31:247-263.

15. Benjamini Y and Hochberg Y. Multiple hypothesis testing and the false discovery rate. JRSSB 1995;57:289-300.

16. Diggle PJ, Liang KY, Zeger SL. Analysis of Longitudinal Data. Clarendon Press, Oxford, Great Britain: 1994

9. Statistical Reports/Shell Tables

a. Trial Monitoring

A. INTAKE

IN Table 1: Number of Patients enrolled by month and country

Month (starting in August when first patient is enrolled)

Country	1	2	3	……………	24	Total
UK						
Canada						
US-VA						

Total

INTable 1a: Number of Patients enrolled by month and hospital-UK

Define sites that are open for enrollment

			Month			
Hospital	1	2	3	……………	24	Total
1						
2						
.						
n						

Total						

INTable 1b: Number of Patients enrolled by month and hospital-Canada

Define sites that are open for enrollment

			Month			
Hospital	1	2	3	……………	24	Total
1						
2						
.						
n						

Total						

INTable 1c: Number of Patients enrolled by month and hospital-US-VA

Define sites that are open for enrollment

			Month			
Hospital	1	2	3	……………	24	Total
1						
2						
.						
n						

Total						

INTable 2: Exclusions from analysis by country

		Reason				
Country	1	2	3	……………	n	Total
UK						
Canada						
US-VA						

Total						

Where

1=

2=

3=

.

.
n=

INTable 3: Minor violations (Not excluded from analysis)

	Reason					
Country	1	2	3	……………	n	Total
UK						
Canada						
US-VA						
Total						

Where
1=
2=
3=
.
.
n=

INTable 4: Number Randomized In CD4 strata

	Country			
Stratum	UK	Canada	US-VA	Total
CD4 Stratum1				
CD4 Stratum2				
Total				

B. BASELINE/RANDOMIZATION

BASETable 5: Baseline Characteristics

**Demographic Variables**	UK	Canada	US-VA	Total
Age (years)				
Means (SD)				
Age Categories				
<20				
20-30				
30-40				
Gender (n,%)				
Female				
Male				
Race				

Risk factors/Mode of infection
- Blood Transfusion
- Other Blood Contact
- IV drug use
- Heterosexual
- MSM
- Other
- Missing/Unknown

AIDS Event

OI Medications for prophylaxis
- Anti-PCP/CMV
- Antibacterial
- Antifungal

BASETable 6: Antiretroviral Therapy History

	UK	Canada	US-VA	Total
Protease Inhibitors				
1				
2				
>2				
NNRTIs				
NRTIs				

BASETable 7: Current Antiretroviral Medication (Prior to Randomization)

	UK	Canada	US-VA	Total
**Number of ART medications**				
Two (2)				
1 PI + 1 NNRTI				
1 PI + 1NRTI				
1 NRTI + 1 NNRTI				
Other				
Three (3)				

1 PI + 2 NNRTI
1 NNRTI + 2 NRTI
3 NRTI
Other

Four (4)

2 PI + 2 NNRTI
1 PI + 1 NNRTI + 2 NRTI
3 NRTI + 1 PI
Other

BASETable 8: Baseline Virological and Immunological Markers

	UK	Canada	US-VA	Total
**Virological Markers**				
HIV RNA (mean log)				
<5				
5-50				
50-100				
>100				
% of patients with phenotypic resistance to:				
No ART drugs				

PI
0
1
2
≥ 2

NRTI
0
1
2
3
≥ 3

NNRTI

Immunological Markers

CD4 cells/mm^3

C. FOLLOW-UP

i. ART

FTable 9: First ART Regimen On-study

	UK	Canada	US-VA	Total
**1 Class of ART drugs**				
≤3 drugs				
4 drugs				
5 drugs				
6 drugs				
≥7 drugs				
**2 Classes of ART drugs**				
≤3 drugs				

4 drugs
5 drugs
6 drugs
≥7 drugs

≥3 Classes of ART drugs
≤3 drugs
4 drugs
5 drugs
6 drugs
≥7 drugs

FTable 10: Follow-up ART Regimen

	UK	Canada	US-VA	Total
**1 Class of ART drugs**				
≤3 drugs				
4 drugs				
5 drugs				
6 drugs				
≥7 drugs				
**2 Classes of ART drugs**				
≤3 drugs				
4 drugs				
5 drugs				
6 drugs				
≥7 drugs				
**≥3 Classes of ART drugs**				
≤3 drugs				
4 drugs				
5 drugs				
6 drugs				
≥7 drugs				

ii. Markers

FFigure 1: Log10 HIV RNA Follow-up : Overall and by Country

FFigure 2: Mean/Median CD4 count follow-up: Overall and by Country

iii. Performance

FTable 11: Process Results – By Country

	Country			
	UK	Canada	US-VA	Total
# enrolled				
# (%) visits in				

window

(%) visits
missed

Proportion of
forms missing

proportion of outstanding
forms with queries

FTable 11a **Process Results – By Hospital, UK**

	Site				
	1	2	. .	n	Total
# enrolled					
# (%) visits in window					
# (%) visits missed					
Proportion of forms missing					
proportion of outstanding forms with queries					

FTable 11b **Process Results – By Hospital, Canada**

	Site				
	1	2	. .	n	Total
# enrolled					
# (%) visits in window					
# (%) visits missed					
Proportion of forms missing					
proportion of outstanding forms with queries					

FTable 11c **Process Results – By Hospital, US-VA**

	Hospital 1 2 . . n	Total
# enrolled		
# (%) visits in window		
# (%) visits missed		
Proportion of forms missing		
proportion of outstanding forms with queries		

D. STRATEGY COMPLIANCE

CFigure 3a: Number of patients on strategy (compliance) and number of patients on Mega-ART by Length of follow-up

CFigure 3b: Number of patients on strategy (compliance) and number of patients on Standard-ART by Length of follow-up

CFigure 3c: Duration of ARDFP and Total Number of patients (Mega vs Standard-ART)

E. AE

AETable 12a: Grade 3 and 4 Adverse Events (led to change in anti-HIV treatment)

	UK	Canada	US-VA	Total
Grade 3				
List coding events				
Grade 4				
List coding events				

AETable 12b: Grade 3 and 4 Adverse Events (did not lead to change in anti-HIV treatment)

	UK	Canada	US-VA	Total
Grade 3				

List coding events

Grade 4

List coding events

AETable 13: Serious Adverse Events

	UK	Canada	US-VA	Total
**Fatal**				
**Life-threatening**				
**Hospitalization**				
At least one hospitalization				
**Diagnosis**				
Listing of Diagnoses				

F. OUTCOMES

OUTTable 14: Outcomes

	UK	Canada	US-VA	Total
Death				
HIV-related				
Drug-related				
New AIDS illness				
Recurrent AIDS illness				

----

b. DSMB Monitoring Tables

Section A: Tables and figures provided at every DSMB meeting

Table 1: Number of Patients enrolled by Treatment Strategy and country

	Treatment Strategy				
Country	ARDFP+ Standard	ARDFP+Mega	No ARDFP+ Standard	No ARDFP+ Mega	Total
UK					
Canada					
US-VA					
Total					

Table 2: Exclusions from analysis by country

Country	Reason 1	2	3	……………	n	Total
UK						
Canada						
US-VA						
Total						

Where

1=
2=
3=
.
n=

Table 3: Minor violations (Not excluded from analysis)

Country	Reason 1	2	3	……………	n	Total
UK						
Canada						
US-VA						
Total						

Where

1=
2=
3=
.
n=

Table 4: Number Randomized In CD4 strata

Stratum	Country UK	Canada	US-VA	Total
CD4 Stratum1				
CD4 Stratum2				
Total				

Table 5: Baseline Characteristics

	ARDFP+ Standard	ARDFP+Mega	No ARDFP+ Standard	No ARDFP+ Mega	Total
**Demographic Variables**					

Age (years)
Means (SD)
Age Categories
<20
20-30
30-40
Gender (n,%)
Female
Male
Race

Risk factors/Mode of infection
Blood Transfusion
Other Blood Contact
IV drug use
Heterosexual
MSM
Other
Missing/Unknown

AIDS Event

OI Medications for prophylaxis
Anti-PCP/CMV
Antibacterial
Antifungal

Table 6: Antiretroviral Therapy History

	ARDFP+ Standard	ARDFP+Mega	No ARDFP+ Standard	No ARDFP+ Mega	Total
PIs					
1					
2					
>2					
NNRTIs					
NRTIs					

Table 7: Current Antiretroviral Medication (Prior to Randomization)

	ARDFP+ Standard	ARDFP+Mega	No ARDFP+ Standard	No ARDFP+ Mega	Total
**Number of ART medications**					
Two (2)					
1 PI + 1 NNRTI					
1 PI + 1NRTI					
1 NRTI + 1 NNRTI					
Other					
Three (3)					
1 PI + 2 NNRTI					
1 NNRTI + 2 NRTI					
3 NRTI					
Other					
Four (4)					
2 PI + 2 NNRTI					
1 PI + 1 NNRTI + 2 NRTI					
3 NRTI + 1 PI					
Other					

Table 8: Baseline Virological and Immunological Markers

	ARDFP+ Standard	ARDFP+Mega	No ARDFP+ Standard	No ARDFP+ Mega	Total
**Virological Markers**					
HIV RNA (mean log)					
<5					
5-50					
50-100					
>100					
% of patients with phenotypic resistance to:					
No ART drugs					
PI					

0
1
2
≥ 2

NRTI
0
1
2
3
≥ 3

NNRTI

Immunological Markers

CD4 cells/mm^3

AFTER RANDOMIZATION/ FOLLOW-UP

Table 1a: First ART Regimen On-study

ARDFP+ Standard

1 Class of ART drugs
≤3 drugs
4 drugs
5 drugs
6 drugs
≥7 drugs

2 Classes of ART drugs
≤3 drugs
4 drugs
5 drugs
6 drugs

≥7 drugs

≥3 Classes of ART drugs
≤3 drugs
4 drugs
5 drugs
6 drugs
≥7 drugs

Table 1b: First ART Regimen On-study

ARDFP+ Mega

1 Class of ART drugs
≤3 drugs
4 drugs
5 drugs
6 drugs
≥7 drugs

2 Classes of ART drugs
≤3 drugs
4 drugs
5 drugs
6 drugs
≥7 drugs

≥3 Classes of ART drugs
≤3 drugs
4 drugs
5 drugs
6 drugs
≥7 drugs

Table 1c: First ART Regimen On-study

No ARDFP+ Standard

1 Class of ART drugs
≤3 drugs
4 drugs
5 drugs
6 drugs
≥7 drugs

2 Classes of ART drugs
≤3 drugs
4 drugs
5 drugs
6 drugs
≥7 drugs

≥3 Classes of ART drugs
- ≤3 drugs
- 4 drugs
- 5 drugs
- 6 drugs
- ≥7 drugs

Table 1d: First ART Regimen On-study

No ARDFP+ Mega

1 Class of ART drugs
- ≤3 drugs
- 4 drugs
- 5 drugs
- 6 drugs
- ≥7 drugs

2 Classes of ART drugs
- ≤3 drugs
- 4 drugs
- 5 drugs
- 6 drugs
- ≥7 drugs

≥3 Classes of ART drugs
- ≤3 drugs
- 4 drugs
- 5 drugs
- 6 drugs
- ≥7 drugs

Table 2a: Grade 3 and 4 Adverse Events (led to change in anti-HIV treatment)

ARDFP+ Standard ARDFP+Mega No ARDFP+ Standard No ARDFP+ Mega Total

Grade 3
- List coding events

Grade 4
- List coding events

Table 2b: Grade 3 and 4 Adverse Events (did not lead to change in anti-HIV treatment)

ARDFP+ Standard ARDFP+Mega No ARDFP+ Standard No ARDFP+ Mega Total

Grade 3
List coding events

Grade 4
List coding events

Table 3: Serious Adverse Events

	ARDFP+ Standard	ARDFP+Mega	No ARDFP+ Standard	No ARDFP+ Mega	Total
**Fatal**					
**Life-threatening**					
**Hospitalization**					
At least one hospitalization					
**Diagnosis**					
Listing of Diagnoses					

Table 4a: Follow-up ART Regimen

	ARDFP + Standard
**1 Class of ART drugs**	
≤3 drugs	
4 drugs	
5 drugs	
6 drugs	
≥7 drugs	
**2 Classes of ART drugs**	
≤3 drugs	
4 drugs	
5 drugs	
6 drugs	
≥7 drugs	
**≥3 Classes of ART drugs**	

≤3 drugs
4 drugs
5 drugs
6 drugs
≥7 drugs

Table 4b: **Follow-up ART Regimen**

ARDFP + Mega

1 Class of ART drugs
≤3 drugs
4 drugs
5 drugs
6 drugs
≥7 drugs

2 Classes of ART drugs
≤3 drugs
4 drugs
5 drugs
6 drugs
≥7 drugs

≥3 Classes of ART drugs
≤3 drugs
4 drugs
5 drugs
6 drugs
≥7 drugs

Table 4c: **Follow-up ART Regimen**

No ARDFP + Standard

1 Class of ART drugs
≤3 drugs
4 drugs
5 drugs
6 drugs
≥7 drugs

2 Classes of ART drugs
≤3 drugs
4 drugs
5 drugs
6 drugs
≥7 drugs

≥3 Classes of ART drugs

≤3 drugs
4 drugs
5 drugs
6 drugs
≥7 drugs

**Table 4d:**	**Follow-up ART Regimen**
	No ARDFP + Mega
**1 Class of ART drugs**	
≤3 drugs	
4 drugs	
5 drugs	
6 drugs	
≥7 drugs	
**2 Classes of ART drugs**	
≤3 drugs	
4 drugs	
5 drugs	
6 drugs	
≥7 drugs	
**≥3 Classes of ART drugs**	
≤3 drugs	
4 drugs	
5 drugs	
6 drugs	
≥7 drugs	

Figure 1a: Number of patients on strategy (compliance) and number of patients on ARDFP + Standard-ART by Length of follow-up

Figure 1b: Number of patients on strategy (compliance) and number of patients on ARDFP + Mega-ART by Length of follow-up

Figure 1c: Number of patients on strategy (compliance) and number of patients on No ARDFP + Standard-ART by Length of follow-up

Figure 1d: Number of patients on strategy (compliance) and number of patients on No ARDFP + Mega-ART by Length of follow-up

Figure 1e: Duration of ARDFP and Total Number of patients (Mega vs Standard-ART)

Section B: Tables and figures provided at Interim Analysis

Table 1: Outcomes

	ARDFP+ Standard	ARDFP+Mega	No ARDFP+ Standard	No ARDFP+ Mega	Total
Death					
HIV-related					
Drug-related					
New AIDS illness					
Recurrent AIDS illness					

Figure 1: Log10 HIV RNA Follow-up : Overall and by Treatment Strategy

Figure 2: Mean/Median CD4 count follow-up: Overall and Treatment Strategy

Figure 3: Virological Marker HIV RNA log10 (means. Standard errors); one plot for each strategy

Figure 4: CD4 cells/mm3 (mean/median); one plot for each strategy

Life Tables and Figures: By strategy Arm (overall and by country) (Non-stratified p-value)

Time to new AIDS event or Death
Time to new or recurrent AIDS illness or Death
Time to Death
Time to first Serious Adverse Event

10. Study Feasibility

The sample size of 504 will be accrued over a period of 4.5 years

<u>VA Patient Accrual</u>

<u>VA HIV population</u>

The VA system cares for approximately 19,000 HIV patients who have access to HIV-knowledgeable health care providers, to the full spectrum of approved antiretroviral medications, and to laboratories that perform virologic and immunological testing. Providers from **30** VA medical facilities had originally expressed interest in participating in the study and provided conservative estimates, indicating that the required number of patients should be easily accruable within the VA system.

During the planning process for OPTIMA surveys and questionnaires sent to potential participating sites had indicated that the target accrual rate could be achieved. Subsequently, assessment of the eligible pool of patients in the VA in light of the revised eligibility criteria has been carried out using site surveys and the ICR database. This assessment confirmed that the target accrual rate in the VA under the revised sample size of 504 is feasible.

In October 2002, ICR database queries were undertaken to assess the continued feasibility of enrollment in the OPTIMA trial. These queries were run for data from the currently-open VA participating sites. The general criteria used to determine this were:

1. History of receiving at least one prescription from the VA system for a drug in \each of the three main classes of antiretrovirals up through December 31, 2001.

2. Currently receiving any anti-retroviral treatment (at least one ART drug) in any 2 or more single-month periods in the quarter under review (e.g. if reviewing Jan-Mar 2002 quarter, a prescription filled in January and March would qualify)

3. At least one CD4 count ≤300 cells/mm^3 in a 6-month period prior to the target quarter.

4. At least one HIV viral load >5,000 copies/ml in the 6 months prior to the quarter under review.

The results from these queries are shown in the table below

	Jan-Mar 2002	Apr-Jun 2002
Patients in VA Care	7933	7883
Meet OPTIMA criteria	530 (6.7%)	548 (7.0%)
Patients in both quarters	338	338
Unique patients in each quarter	192	210

These results suggest that the number of OPTIMA eligible patients is slightly increasing (0.3% increase); the most important finding however is that the pool of unique and eligible patients has increased. It is evident from this analysis that the revised target sample size is feasible.

UK Patient Accrual

In light of poor recruitment in the UK for reasons outlined in other areas of this document, the UK currently expects to recruit one patient per month.

Canada Patient Accrual

The best estimate for the accrual rate in Canada over the next few years is based the accrual experience thus far. Over the last year, Canadian sites have averaged two patients per month. The number per month has been fairly consistent with a monthly maximum of 4 patients and a minimum of zero. Accrual could be augmented slightly by the recent addition of two large sites in Vancouver and Montreal.

OPTIMA Sites: UK, Canada and US-VA

The following is a table summarizing potential OPTIMA sites in the three countries:

Country	City	Site
**UK Open Sites**		
	Belfast	Royal Victoria Hospital
	Brighton	Brighton General Hospital
	Cambridge	Addenbrooke's Hospital
	Colchester	Essex County Hospital
	Edinburgh	Western General Hospital
	Leicester	Leicester Royal Infirmary
	London	Central Middlesex Hospital
	London	Chelsea and Westminster Hospital
	London	Royal Free Hospital
	London	St. Mary's Hospital
	London	St. Thomas' Hospital
	Oxford	Churchill Hospital
	Peterborough	Peterborough District Hospital
	Portsmouth	St. Mary's Hospital
	Sheffield	Royal Hallamshire Hospital
**Total open sites**	**15**	
**UK Pending Sites**		
	Birmingham	Heartlands Hospital
	Blackpool	Blackpool Victoria Hospital
	Dublin	St. Jame's Hospital
	Gloucestershire	Gloucestershire Royal Hospital
	Kent	Kent and Canterbury Hospital
	Liverpool	Royal Liverpool University Hospital
	London	Kings College Hospital
	London	Newham General Hospital

Country	City	Site
	London	St. George’s Hospital
	Newcastle	Newcastle General Hospital
**Total Pending Sites**	10	
**CANADA Open Sites**		
	Calgary	Southern Alberta HIV Clinic
	Edmonton	University of Alberta Hospital
	Fleurimont	Centre Hopitalier Universite de Sherbrooke
	Halifax	Victoria General Hospital
	Hamilton	McMaster Health Science Centre
	Kingston	Queen’s University
	London	St. Joseph’s Health Care Centre
	Montreal	Montreal Chest/Royal Victoria Hospital
	Montreal	CHUM – Campus Hotel-Dieu
	Montreal	Montreal Clinique L’Actuel
	Ottawa	University of Ottawa Health Services
	Ottawa	Ottawa General Hospital
	Saskatoon	Royal University Hospital
	Ste Foy	Centre Hopitalier de l’universite de Laval
	Sunnybrook	Sunnybrook Health Science Centre
	Toronto	Toronto General Hospital
	Toronto	St. Michael’s Hospital
	Vancouver	Downtown Infectious Diseases Clinic
	Vancouver	St. Paul’s Hospital (IDC)
	Victoria	Cool Aid Community Health Centre
	Winnipeg	University of Manitoba
**Total Open sites**	**21**	
**CANADA Pending Sites**		
	Vancouver	Spectrum (Viron)
	Montreal	McGill University Health Centre
**Total Pending Sites**	2	
**US**		
	Ann Arbor	VAMC 506
	Atlanta	VAMC 508
	Baltimore	VAMC 512
	Bay Pines	VAMC 516
	Boston	VAMC 523

Country	City	Site
	Bronx, NY	VAMC 526
	Chicago, Hines	VAMC 578
	Chicago /W-side	VAMC 537
	Cleveland, OH	VAMC 541
	Dallas	VAMC 549
	Durham	VAMC 558
	East Orange	NJ HCS 561
	Gainesville	VAMC 573
	Houston	VAMC 580
	Long Beach	VAMC 600
	Los Angeles	VAMC 691
	Miami	VAMC 546
	Palo Alto	VAMC 640
	Phoenix	VAMC 644
	Portland	VAMC 648
	San Antonio	VAMC 671
	San Diego	VAMC 664
	San Juan	VAMC 672
	Columbia, SC	VAMC 544
	West Haven	VAMC 689
**Total Open sites**	**25**	
**TOTAL Open Sites**	**61**	
**Total Pending Sites**	**12**	
**TOTAL SITES**	**73**	

Study Feasibility in light of eligibility criteria modifications (v1.1)

Eligibility criteria were implemented in order to reflect current clinical practice. After analysis of data from three cohorts (1 US and 2 European) revealed that the patient pool would increase by 40% using the revised criteria, an assessment of the impact of these modifications on accrual at the OPTIMA sites was undertaken.

In the US (VA), participating sites were asked to compare the total number of patients attending their clinics meeting the new vs. old CD4 and viral load criteria. This survey

suggested a 41% increase in the pool of patients who would be considered for participation in OPTIMA. A similar assessment in the UK done using data from 4 clinical centers (representative of the UK sites) and other cohort data suggested a 40% increase in the pool of patients. Analysis of cohort data from Canada revealed an increase of at least 25% in the numbers of patients who would be considered for the trial (this was obtained using CD4 <=250 cells/mm^3 as the cutoff level).
